# 14th Panhellenic Conference on Alzheimer’s Disease and the 6th Mediterranean Conference on Neurodegenerative Diseases, 13 February–16 February, Thessaloniki, Greece

**DOI:** 10.3390/neurosci6030057

**Published:** 2025-06-20

**Authors:** Magda Tsolaki

**Affiliations:** 1 Laboratory of Neurodegenerative Diseases, Center of Interdisciplinary Research and Innovation (CIRI-AUTH), Balcan Center, Buildings A & B, 57001 Thessaloniki, Greece; tsolakim1@gmail.com; 2 Greek Association of Alzheimer’s Disease and Related Disorders, 54643 Thessaloniki, Greece; 3 1st Department of Neurology, School of Medicine, Faculty of Health Sciences, Aristotle University of Thessaloniki, 54636 Thessaloniki, Greece

**Keywords:** dementia, alzheimer, parkinson, neurodegenerative diseases, new technologies, caregivers, non-pharmacological interventions, pharmacological interventions, home care, palliative care

## Abstract

At the 14th Panhellenic Conference on Alzheimer’s Disease and 6th Mediterranean Conference on neurodegenerative diseases, we experienced an exciting journey, following the patient through the stages of their neurodegenerative disease: onset, diagnosis, progression, and eventual outcome. Fighting alongside him are researchers, doctors, psychologists, biologists, chemists, pharmacists, nurses, trainers, physiotherapists, speech therapists, occupational therapists, electrical engineers, architects, and other scientists, even actors and musicians, who aim to prevent and cure the disease, limit its progression, and improve the quality of life of those affected by it. Among them, their caregivers stand out as the most dedicated companions. In a collection of abstracts that reflects the work of all of the above, we capture the results of our biennial scientific meeting, which, thanks to them, is constantly evolving in a promising way.

## 1. Introduction

We are all living in a time of fast scientific progress. The amount of ongoing, diverse research that is already being tested and making an impact is overwhelming. As medical professionals who work tirelessly and passionately with patients who have neurodegenerative disorders and their caregivers, it is our responsibility, as well as a necessity, to constantly provide feedback, sharing our knowledge and experience.

Organizing the Panhellenic Conference on Alzheimer’s Disease and the Mediterranean Conference on Neurodegenerative Diseases is always a challenge, but it has become our commitment as we continue to progress and hope for a cure. On 13–16 February 2025, the 14th Panhellenic Conference on Alzheimer’s Disease and the 6th Mediterranean Conference on Neurodegenerative Diseases took place. The conferences were hosted over four days, in four parallel halls, with about 400 multidisciplinary speakers and Presidents participating from all over the world. The conference was attended by more than 700 people. Our program covered a wide range of highly significant topics, including the safe use of anti-amyloid drugs, neuropsychological assessment and its future, the role of the caregiver in the quality of life of the family, new care units for end-stage dementia patients, and highlights from European programs and technologies alongside dementia care.

Health professionals, people with dementia, caregivers, students, and young scientists attended the conferences in order to be informed in a reliable and up-to-date framework about the latest developments, as well as to participate in workshops and presentations by young neuroscientists from all over the country on Alzheimer’s disease and other neurodegenerative diseases, both in the diagnostic and therapeutic field.

A notable guest at the conference was the Mayo Clinic–Rochester Professor of Neurology–Epidemiology, Walter Rocca.

The conference was under the auspices of the Greek Ministry of Health, Alzheimer Disease International, the Municipality of Thessaloniki, the Hellenic Association of Geriatrics and Gerontology, the Faculty of Health Sciences A.U.Th., the School of Medicine, the Greek Medical Association, the Panhellenic Pharmaceutical Association, the Greek Red Cross, the Aristotle University of Thessaloniki, the International Medical Olympicus Association, the Medical Association of Thessaloniki, the Region of Central Macedonia, and the Panhellenic Federation of Alzheimer’s Disease and Related Disorders, and received 26 points for Continuing Medical Education (CME-CPD credits) from the Greek National Medical Association (Ref: 6300/LEE24-00144).

To ensure excellent scientific support, 112 members of the International and Panhellenic Scientific Committee, who also reviewed the 307 abstracts that we received, collaborated with 26 members of the Organizing Committee.

We invite you to read a selection of these abstracts, which represent the fusion of sciences at the service of the patient. 

Yours sincerely,

Magda Tsolaki

President of the Organizing Committee

## 2. Conference Abstracts

### 2.1. Proteomic Analysis for the Investigation of Biomarkers in the Blood of Patients with Alzheimer’s Disease: A Systematic Review

**Kontos Christos ^1^, Andreadou Eleni ^2^, Tsolaki Magda ^3^ and Scarmeas Nikolaos ^4,5^**

^1^  Medicine School, Aristotle University of Thessaloniki, 54124, Greece
^2^  Department of Biomedical Sciences, School of Health Sciences, International Hellenic University, 57400, Sindos, Thessaloniki, Greece
^3^  Center for Interdisciplinary Research and Innovation, Laboratory of Neurodegenerative Diseases (LND), 57001, Thermi, Thessaloniki, Greece
^4^  1st Department of Neurology, Aiginition Hospital, Athens Medical School, National and Kapodistrian University, 11528 Athens, Greece
^5^  Department of Neurology, The Gertrude H. Sergievsky Center, Taub Institute for Research in Alzheimer’s Disease and the Aging Brain, Columbia University, New York, NY 10027, USA

**Abstract:** Biomarkers are quantifiable biological parameters that serve as potential indicators for prognosis, diagnosis, or response to the applied treatment of various pathologies. They indicate the underlying pathophysiological process and ideally should be detected at molecular onset and altered during disease progression. Proteomics is a representative part of the effort to discover new biomarkers and is defined as the systematic study of the protein complement of organisms known as proteome. The field of proteomics aims at either detecting individual proteins or the quantification of all proteins of a cell, tissue, or organism. Ιn medicine proteomics may involve either the determination of peptides in specific samples and the identification of proteins in human tissues, based on the recovery of similar proteins from databases and de novo prediction, or the understanding of physiology and pathology based on large-scale data sets. Even more advanced technologies and minimally invasive screening tests are available for these purposes, such as the two-dimensional electrophoresis, the chemiluminescence, and the widely used mass spectrometry. In this study, we will review the contribution of proteomics to the investigation of biomarkers exclusively in the blood of patients with the pathology of the most common neurodegenerative disorder, Alzheimer’s Disease.

**Keywords:** blood biomarkers; proteomics; proteome; Alzheimer’s disease

### 2.2. Sequence Learning in the Human Cortical and Subcortical Brain: A (Coordinate-Based) Meta-Analysis

**Styliani Kassiani Tsantzalou ^1,2^ and Frank Van Overwalle ^1^**

^1^  Faculty of Psychology and Center for Neuroscience, Vrije Universiteit Brussel, Pleinlaan 2, 1050, Brussels, Belgium
^2^  School of Medicine, University of Thessaloniki, Campus 54124, Thessaloniki, Greece

**Abstract: Background**: The present ongoing study aims to update the meta-analysis [1] conducted on sequence learning within the visuomotor domain, expanding it to incorporate non-motor domains such as cognitive, social, and affective processing. Using an Activation Likelihood Estimation (ALE) meta-analysis of functional magnetic resonance imaging (fMRI) data, we investigate the roles of cortical and subcortical structures in both motor and non-motor sequence learning. **Methods:** A comprehensive search of PubMed identified 1525 studies, narrowed to 1307 studies from 2005 onwards, reflecting the growing body of neuroimaging literature in social and affective domains. Inclusion criteria focused on functional neuroimaging studies involving healthy adult participants, with reported whole-brain or region-of-interest (ROI) analyses, utilizing standard reference coordinates (Talairach-Tournoux/MNI). We systematically extract data on participant demographics, experimental paradigms, and relevant contrasts, prioritizing conditions that compare learned sequences against random or novel sequences. **Results:** The meta-analysis will provide a detailed understanding of the neural correlates underpinning sequence learning across various domains. We expect our results to reveal distinct and overlapping cortical and subcortical contributions in sequence learning, highlighting conditions with the most learning opportunities or prediction errors. **Conclusions:** Our findings will offer insights into motor, non-motor, social, and nonsocial sequence processing mechanisms, contributing to a broader understanding of the neural basis of sequence learning.

**Keywords:** sequence learning; fMRI; Serial Reaction Time (SRT); implicit/explicit memory; motor memory

### 2.3. Greek Adaptation of the Saturn Cognitive Screening Test and Initial Validation in a Greek Older Adult Population

**Vasiliki Dimaki ^1^, Alexandra Galani ^1^, Chrysa Terzi ^1^, Francesco Giaquinto ^2^, Sara Assecondi ^3^, Paola Angelelli ^4^, Moses Gialaouzidis ^5^, Madga Tsolaki ^5^, Stelios Zygouris ^6^, Amaryllis Malegiannaki ^1^**

^1^  Department of Psychology, University of Western Macedonia, Florina, Greece
^2^  Department of Human and Social Sciences, University of Salento, Lecce, Italy
^3^  Center for Mind/Brain Sciences-CIMeC, University of Trento, Rovento (TN), Italy
^4^  Department of Experimental Medicine, University of Salento, Lecce, Italy
^5^  Greek Alzheimer Association, Thessaloniki, Greece
^6^  Department of Psychology, Democritus University of Thrace, Didymoteicho, Greece

**Abstract: Background**: The increased prevalence of cognitive disorders necessitates the creation of sensitive tools for the differentiation between age-related and pathological cognitive decline in older adults. The aim of the present study was to evaluate and adapt the Greek version of Self-Administered Task Uncovering Risk of Neurodegeneration (SATURN), an open-source, self-administered cognitive screening test. **Methods:** A total 159 older adults (age: M = 68.11, SD = 6.17 years; education: M = 13.72, SD = 3.74 years). Participants were administered the Greek versions of the Mini Mental State Examination (MMSE), the Montreal Cognitive Assessment (MoCA), the Short Anxiety Screening Scale (SAST), the Geriatric Depression Scale (GDS), and the SATURN. **Results:** Saturn score correlated positively with MMSE score (r = 0.278, *p* < 0.001) and MoCA score (r = 0.214, *p* = 0.007). No significant correlations were found between SATURN score and GDS and SAST scores. Thus, results indicate that SATURN has good convergent validity with gold standard cognitive screening tests for older adults and good divergent validity with scales assessing affective disorders. **Conclusions:** In conclusion, the results of the present study confirm that the SATURN tool is valid instrument for cognitive screening in Greek older adults.

**Keywords:** mild cognitive impairment computerized cognitive assessment

### 2.4. Self-Report of Cognitive Function in Older Adults: Investigating Its Relationship with Traditional and Computerized Measures of Cognition

**Chrysa Terzi ^1^, Vasiliki Dimaki ^1^, Alexandra Galani ^1^, Francesco Giaquinto ^2^, Sara Assecondi ^3^, Paola Angelelli ^4^, Moses Gialaouzidis ^5^, Madga Tsolaki ^5^, Stelios Zygouris ^6,7^, Amaryllis-Chryssi Malegiannaki ^1^**

^1^  Department of Psychology, University of Western Macedonia, Florina, Greece
^2^  Department of Human and Social Sciences, University of Salento, Lecce, Italy
^3^  Center for Mind/Brain Sciences-CIMeC, University of Trento, Rovereto (TN), Italy
^4^  Department of Experimental Medicine, University of Salento, Lecce, Italy
^5^  Greek Alzheimer Association, Thessaloniki, Greece
^6^  Department of Psychology, Democritus University of Thrace, Didymoteicho, Greece
^7^  Global Brain Health Institute, San Francisco, USA

**Abstract: Background:** Aging is often accompanied by memory impairment. The focus of the present study is older adults’ perception of memory through self-reports and its relationship with their objective memory performance as measured with cognitive assessment tests. **Methods:** A total of 172 older adults (135 healthy and 38 diagnosed with MCI) with a mean age of 69.09 years (SD = 6.32) participated in the study. They were administrated self-report questionnaires regarding their memory including the Cognitive Failures Questionnaire (CFQ) and the Attention Related Cognitive Errors Scale (ARCES) as well as the Self-Administered Task Uncovering Risk of Neurodegeneration (SATURN), the Mini Mental State Examination (MMSE), the Montreal Cognitive Assessment (MoCA), the Short Anxiety Screening Scale (SAST) and the Geriatric Depression Scale (GDS). **Results:** Group comparisons indicated that cognitive performance of older adults with MCI was significantly lower than the performance of healthy controls when assessed either through objective or self-report measures. Correlational analysis (on the full sample) indicated that cognitive problems self-report scores correlate negatively with objective cognitive performance and positively with anxiety and depression. **Conclusions:** The results of the present study suggest that self-reports provide useful information about the subjective experience of cognitive decline and its relationship with objective cognitive performance and mood and anxiety disorders.

**Keywords:** mild cognitive impairment; subjective cognitive impairment; computerized cognitive assessment; self-report

### 2.5. Assessing Various Cognitive Domains in Older Adults with Computerized Self-Administered and Conventional Examiner-Administered Cognitive Tests

**Alexandra Galani ^1^, Chrysa Terzi ^1^, Vasiliki Dimaki ^1^, Francesco Giaquinto ^2^, Sara Assecondi ^3^, Paola Angelelli ^4^, Ouzouni Fani ^5^, Madga Tsolaki ^5^, Stelios Zygouris ^1,6^, Amaryllis-Chryssi Malegiannaki ^1^**

^1^  Department of Psychology, University of Western Macedonia, Florina, Greece
^2^  Department of Human and Social Sciences, University of Salento, Lecce, Italy
^3^  Center for Mind/Brain Sciences–CIMeC, University of Trento, Rovereto (TN), Italy
^4^  Department of Experimental Medicine, University of Salento, Lecce, Italy
^5^  Greek Alzheimer Association, Thessaloniki, Greece
^6^  Department of Psychology, Democritus University of Thrace, Didymoteicho, Greece

**Abstract: Background**: Age-related or pathological cognitive decline is prevalent among older adults, thus increasing the demand for brief, well-validated cognitive assessment tests. The aim of the present study was to compare two methods for assessing the general cognitive functioning of Greek older adults: traditional examiner-administered cognitive assessment tools and the computerized, Self-Administered Task Uncovering Risk of Neurodegeneration (SATURN) cognitive assessment tool. **Methods:** 126 healthy older adults (age: M = 67.48, *SD* = 5.23 years; education: M = 14.37, *SD* = 3.36 years), recruited from day centers for dementia and from the general public, participated in the study. Participants were administered the SATURN test and examiner-administered cognitive tests comprising the Montreal Cognitive Assessment (MoCA), Mini Mental State Examination (MMSE), Rey Auditory Verbal Learning Test (RAVLT), Rey-Osterrieth Complex Figure Test (ROCFT), Trail Making Test (TMT) and Verbal Fluency Test. **Results:** There were significant correlations between SATURN domain scores for memory and executive function and domain scores or test scores for similar domains in traditional tests. No other SATURN domain scores correlated with traditional tests of the same domains. **Conclusions:** The absence of statistically significant correlations with performance in other cognitive domains could be interpreted based on the format of computerized cognitive tests which may lead to simultaneous activation of many cognitive functions.

**Keywords:** mild cognitive impairment; computerized cognitive assessment; cognitive screening; cognitive assessment

### 2.6. The Right Small Molecules and Biologics in the Era of Molecular Precision Medicine

**Artemissia-Phoebe Nifli ^1,2^**

^1^  University of Thessaly, Greece
^2^  Larissa Association of Alzheimer’s Disease and Related Disorders, Greece

**Abstract:** A great number of drug-based therapies are currently under clinical investigation. However, the need for diversity, equity and inclusion in health research remains, and these principles could extend to the agents themselves. Prevalence, epidemiological trends and risk factors of dementia overlap with those of non-communicable diseases. Therefore, the available standards of care, ranging from small molecules, such as rivastigmin or metformin, to biologics, such as dulaglutide, may be of use. Standard biomarkers, e.g., HbA1c, as well as dementia (core) biomarkers (A, T1, T2, fibrin/fibrinogen, homocysteine) may guide the process. Pharmacogenomics, e.g., CYP2D6 profiling, and drug levels monitoring could be helpful in preventing drug-related problems. Moving fast forward and past experimental active immunotherapy, combination therapies seem the most promising in the field of cognitive health. Considering the extremely high and challenging adverse to severe drug reactions in older people with dementia, it could be of value to revisit Western approaches and incorporate some of the principles of traditional pharmacopoeia and/or microdosing.

**Keywords:** dementia; pharmacotherapy; precision medicine

### 2.7. Primary Progressive Aphasia Guide

**Natalie Ive, Australian Aphasia Association**

What I wish people knew about living with primary progressive aphasia. (PPA) is a language-led dementia that affects individuals’ ability to communicate, express thoughts, and understand words. Still, people face barriers to communication and support due to a lack of understanding of the condition. I was diagnosed with PPA in 2021. Consequently, my team and I at the University of Tasmania co-developed a PPA guide to raise awareness and support. This initiative has made a significant impact, and I urge you to join us in championing this cause. Let’s work together to ensure that individuals living with PPA receive the understanding, support, and resources they deserve. Because of my challenges and frustrations with some people not understanding my condition, I met with Professor Jade Cartwright to discuss the need for a PPA strategy and a strategy to raise awareness for support services. Services and supports that too many people with PPA are missing out on! We organised a Zoom meeting, and there was a lot of robust conversation and great brainstorming ideas for creating the PPA strategy. Ultimately, we developed our amazing PPA guide, co-developed by Catherine Taylor, Natalie Ive, Deborah Hersh, and Jade Cartwright.

### 2.8. Interactions Between the Serotonergic System and Microglia in Preclinical Models of Familial Alzheimer’s Disease

**Athanasios Metaxas ^1^, Marco Anzalone ^2,3^ and Bente Finsen ^2,3^**

^1^  European University Cyprus, School of Sciences, Department of Life Sciences, Nicosia, Cyprus
^2^  Department of Neurobiology, Institute of Molecular Medicine, University of Southern Denmark, Odense, Denmark
^3^  Department of Clinical Research, University of Southern Denmark, BRIDGE-Brain Research-Inter-Disciplinary Guided Excellence, Odense, Denmark

**Abstract: Background**: The serotonergic system, a key regulator of mood and cognition, has garnered much attention in Alzheimer’s disease (AD) research, particularly in the context of modulating neuroinflammation. Microglia—the primary immune cells of the central nervous system—play a critical role in mediating neuroinflammatory responses in both sporadic and familial forms of AD, with the latter characterized by early onset and rapid disease progression. This presentation will explore how serotonergic signaling influences neuroinflammation and microglial phenotypes in preclinical mouse models of familial AD. **Methods:** We investigate the effects of serotonergic modulation on cytokine production and markers of neuroinflammation, using pharmacological agents, immunohistochemistry and molecular imaging techniques. **Results:** Our findings suggest that the serotonergic system has the potential to significantly modulate inflammatory profiles in AD mouse models, in a time-dependent manner. **Conclusions:** Understanding the interplay between serotonin and microglia may provide novel strategies for targeting neuroinflammation in AD and potentially inform the development of therapeutics for other neurodegenerative conditions.

**Keywords:** Alzheimer’s disease; mouse models; serotonin; neuroinflammation

**Funding:** This work was supported by the A.P. Møller og Hustru Chastine Mc-Kinney Møllers Fond (15-267), the University of Southern Denmark (SDU2020, CoPING AD) and the Danish Council for Independent Research (DFF).

### 2.9. Transnational European Project: Distance Education and Vocational Training on Dementia for University Students and Health Care Professionals (DEDUC, Erasmus+)

**Dimitris Bekiaridis-Moschou ^1^, Vasiliki Garopoulou ^2^, Christos Mouzakidis ^3^ and Magdalini Tsolaki ^4^**

^1^  Hellenic Association of Alzheimer’s Disease and Related Disorders (Alzheimer Hellas)
^2^  Medical school, Aristotle University of Thessaloniki, A.U.Th. Greece Panhellenic Institute of Neurodegenerative Diseases Thessaloniki, Greece Hellenic Association of Alzheimer’s Disease and Related Disorders (Alzheimer Hellas)
^3^  Hellenic Association of Alzheimer’s Disease and Related Disorders (Alzheimer Hellas)
^4^  Medical school, Aristotle University of Thessaloniki, Thessaloniki, Greece Department of Classics Neuropsychiatrist, Lab of Neurodegenerative Diseases, CIRI, A.U.Th.

**Abstract:** The present project proposes the development of distance learning systems for the practical component of the training of both university students and health professionals in the fields of Dementia Care, Mild Cognitive Impairment Treatment and Healthy Ageing. **Background:** The project focuses on developing a robust distance learning system, merging digital tools and innovative methods, where trainees can gain practical skills and theoretical knowledge, enhancing accessibility and effectiveness. In this way, health practitioners and university students can be informed on the knowledge and practices of professionals that work in Dementia Care Centers in several European Countries bringing the participating countries closer and improving the services in the field in general. **Methods**: In this project we’ll implement several activities: Analysis of platform requirements and development of a digital learning platform for teaching- learning purposes, development of a vocational training program for dementia care and its implementation, a digital simulation game as a supplementary learning tool, a transfer handbook and policy recommendations. **Results**: Dissemination of all project results. **Conclusions**: The added value of a transnational European dimension is introduced/added to such training by the distance education component.

**Keywords:** vocational training; distance learning; dementia care; European project

### 2.10. Tango and Syrtaki Exchanging Glances: Cross-Cultural Dance and Research Erasmus Project with French and Greek Joined Forces

**Anthoula C. Tsolaki ^1^, Themis Parastatidis ^1^, Anne Bramard-Blagny ^2^, Martin Laplace ^2^, Eleonore de Lamare ^3^ and Magda Tsolaki ^1^**

^1^  Greek Alzheimer’s Association and Related Disorders, Thessaloniki, Greece
^2^  ABB-Reportages, Dijon, France
^3^  EHPAD-PASA-Foyer Notre Dame Beaulieu, France

**Abstract: Background**: The benefits of dancing for elderly are undeniable. As an aerobic exercise as well as a social activity promotes the well-being of the participants by many ways. This impact has been previously reported by research findings including ours. **Methods:** Sixty-four elderly in France and Greece, participated in a non-pharmacological intervention, learning Tango and Greek Traditional Dances. The participants were patients with Dementia, Mild cognitive Impairment or Cognitively healthy individuals at Nursing homes (EHPAD) and Day care centers. The intervention was separated in two parts. The first dedicated to Tango (N = 45) and the second to the Greek Traditional dances (N = 19). Demographic data of all the participants were collected, and their cognitive diagnosis. The pre-post evaluation was conducted by the personnel of the hosting organization included: MMSE, GDS, Short Anxiety Screening Test, EQ-5L-5D and the Fullerton Senior Fitness Test. At the end of the12-week intervention for each Dance category the participants’ self-reported sense of well-being was evaluated using EVIBE scale, on a five-point scale. **Results:** French patients with Dementia (PwD), in EHPAD improved their Quality of life status according to the visual analogue scale from 59% to 68% (*p* = 0.016) after the Tango Intervention, and retained this feeling after the Greek Traditional Dances (70.63%). The Greek Elderly Group who participated in Tango, they were younger, more educated and mostly in the earlier stages of the disease (MCI). They improved significantly almost in every aspect of the EQ-5D-5L: Mobility (*p* = 0.048); Self-Care (*p* = 0.046); Activities (*p* = 0.046); Pain/Discomfort (*p* = 0.038); Anxiety/Depression (*p* = 0.202); Visual Analogue Scale (from ~75% to 95%, *p* = 0.004). The second Greek group, after the Greek Traditional Dancing Intervention, improved the EQVAS, at 86.6364%. The Fullerton Fitness Scale revealed a statistically significant improvement in the aerobic fitness assessment of “Two minutes steps” (*p* = 0.026). **Conclusions:** The most significant outcome of this pilot is the improvement of the quality of life of the elderly. According to the EVIBE and EQ-5D-5L scales, there were no significant difference due to the Dance style, suggesting no cultural differences.

**Keywords:** active ageing; well-being; elderly; neurodegenerative diseases; dance

**Funding:** The Project was under the ERASMUS+ Programme. Agreement Number—2023-2-FR01-KA210-ADU-000185294.

### 2.11. Mild Cognitive Impairment in People Undergoing Hemodialysis

**Nikolaos Gerosideris, Christina Ouzouni, Pinelopi Vlotinou and Ioanna Giannoula Katsouri**

Department of Occupational Therapy, University of West Attica

**Abstract: Background:** Individuals with chronic kidney disease (CKD) frequently experience mild cognitive impairment (MCI), with prevalence rates ranging from 13% to 87%. MCI in this population is associated with an increased risk of mortality, reduced quality of life, and decreased ability to participate in occupational domains such as Instrumental Activities of Daily Living (IADLs), work, leisure, and sleep. Moreover, impairments have been observed in tasks such as transportation, financial management, and medication adherence. **Methods:** The Montreal Cognitive Assessment (MoCA), which is standardized for the Greek population, was administered to assess the overall cognitive function of 60 hemodialysis patients aged 18 years and older. **Results/Conclusions:** The findings of the study indicate that hemodialysis patients (mean age: 80 years) exhibited mild cognitive impairment based on their total MoCA scores. These results contribute to the existing knowledge on cognitive impairment, in people undergoing hemodialysis, and its impact on daily occupational participation. The anticipated findings highlight the need for developing appropriate therapeutic interventions that holistically support people undergoing hemodialysis, promoting their independence in meaningful occupations.

**Keywords:** mild cognitive impairment; chronic kidney disease; hemodialysis; occupational therapy

**Funding:** The project for the conference was funded by University of West Attica.

### 2.12. Epileptic Syndromes in Preterm Neonates: The Impact on Subsequent Cognitive Ability

**Symeon Dimitrios Daskalou ^1^, Christina Ouzouni ^1^, Theodoros Sergentanis ^2^ and Ioanna-Giannoula Katsouri ^1^**

^1^  Department of Occupational Therapy, School of Health and Care Sciences, University of West Attica
^2^  Department of Public Health Policies, School of Public Health, University of West Attica

**Abstract: Background:** Epilepsy is a medical condition for preterm neonates with potentially long-term effects on their cognitive development. This review focuses on understanding the relationship between epileptic syndromes occurring in preterm neonates and their subsequent cognitive abilities. **Methods:** To collect relevant data, a comprehensive search of international literature was conducted, including clinical data analyses and follow-up studies from the scientific databases PubMed and ScienceDirect, covering the period from 2004 to 2024. **Results:** Results indicate that variables such as the frequency and nature of epileptic episodes in preterm neonates, as well as gestational age, the severity of the neonate’s condition, and comorbidities, are associated with long-term neurodevelopmental outcomes and cognitive functions in preschool and school-age children. **Conclusions:** In conclusion, although epilepsy in preterm neonates is linked to an increased risk of developmental issues, early diagnosis and intervention can significantly improve outcomes. Special emphasis is placed on intervention strategies and supportive care that can enhance the developmental trajectory of neonates with a history of epilepsy. This review contributes to a better understanding of the long-term effects of epileptic syndromes in preterm neonates and highlights the need for further clinical studies and research data to improve intervention strategies.

**Keywords:** epileptic syndromes; preterm neonates; cognitive ability

**Funding:** Τhe project for the conference was funded by the University of West Attica.

### 2.13. The Contribution of Coronary Heart Disease (CHD) in the Emergence of Mental Function Disorders

**George Bablekos ^1^, Christina Ouzouni ^1^, Pinelopi Vlotinou ^1^, Georgia Tsakni ^1^, Spyridon Galanakos ^2^, Spyridon-Nikolaos Mavropoulos ^3^ and Ioanna-Giannoula Katsouri ^1^**

^1^  Department of Occupational Therapy, School of Health Sciences and Care, University of West Attica, Athens, Greece
^2^  Primary Health Center, Amarousion, Athens, Greece
^3^  Medical School, University of Patras

**Abstract: Background**: To thoroughly investigate the occurrence of mental function disorders, for patients with preexisting coronary heart disease (CHD). **Methods**: To extract information the PubMed data base was used from 1 January 2000 to 31 August 2024. The search was classified in six different sub-searches by using eight key words such as: “Coronary Disease/complications [Majr], “Mental Disorders/etiology [Majr]”, “Survival Rate [Mesh]”, “Quality of Life [Mesh]”, “Memory”, “Dementia”, “Neurocognitive Disorders/etiology [Majr]”, “Mental Disorders/etiology [Majr]”, The Boolean operators AND & OR, were also used. A hundred forty-two (142) articles were emerged. **Results**: The grading of coronary artery disease is associated with substantial damages of the Alzheimer disease (AD). A linkage between angina’s pectoris severity and cognitive impairments’ disturbances, focusing on preexisting CHD, is established. Platelets’ hyperactivity contributes further to dementia’s emergence. Age, myocyte damage, inflammation, subclinical atherosclerosis, vascular function, physical activity, hepatic steatosis and history of cancer, enhance the possibility for dementia’s occurrence. The younger the age of onset of CHD the more likely is the risk for mental function disorders, including both AD and vascular dementia. **Conclusions**: The latter is of great importance to apply timely preventive therapeutic interventions, in order to control the risk for future cognitive disorders.

### 2.14. The Link Between Pain and Depression: Insights from Transcranial Direct Current Stimulation (tDCS)

**Fereshteh Sedaghat ^1,2^, Amin Rakhshani ^1,2^ and Stavros J. Baloyannis ^1^**

^1^  Aristotle University of Thessaloniki, School of Medicine, AHEPA hospital
^2^  Outpatient Memory Clinic, Mashhad

**Abstract: Background**: The relationship between chronic pain and depression has been a topic of research and debate for over 40 years. Studies utilizing Positron Emission Tomography (PET) and Single Photon Emission Computed Tomography (SPECT) have demonstrated reduced blood flow and metabolic activity in the brain, particularly within the frontal regions, in patients suffering from both chronic pain and depression. This finding suggests that there may be overlapping functional anatomy in the nociceptive and affective pathways. **Methods:** In our investigation, we sought to further explore this relationship using transcranial direct current stimulation (tDCS). Our results indicate that while chronic pain and depression may share common functional anatomy, especially in the frontal and prefrontal areas, there are likely distinct underlying mechanisms involved as well. **Results:** tDCS emerges as a promising therapeutic tool for modulating both pain syndromes and mood disorders. By leveraging this innovative technique, we hope to gain deeper insights into the intricacies of brain function and the interconnectedness of pain and depression.

**Keywords:** depression; chronic pain; tDCS; transcranial direct current

### 2.15. Plasma β Amyloid and p-Tau Levels in Patients with Dementias and MCI—A Study with SPECT Confirmation—Twenty Years Later!

**Fereshteh Sedaghat, Alexandra Papazisi, Amin Rakhshani, Vassiliki Tsavdaridou, Christina Aggouridaki and Stavros Baloyannis**

Aristotle University of Thessaloniki, School of Medicine, AHEPA hospital

**Abstract: Background**: Two decades ago, our study was pioneering in measuring plasma phospho-tau and β-amyloid 1-42 levels to find non-invasive procedure for differential diagnosis of dementias. At that time, the correlation between plasma β-amyloid 1-42 and p-tau in various dementias lacked exploration. **Methods:** Our investigation involved 74 individuals undergoing perfusion SPECT and DaTscan, including 29 AD, 3 Fronto-temporal Dementia, 6 Dementia with Lewy Bodies, 13 Vascular Cognitive Impairment, 7 MCI patients, and 16 controls. **Results:** Our results showed significant high Aβ42 levels in DLB patients (229.9 ± 125.9 pg/mL) and significant increases in MCI patients (57.9 ± 33.3 pg/mL) compared to controls (*p* ≤ 0.000). Additionally, p-tau was detectable in plasma of 14 patients across different dementia types, with DLB patients, exhibiting higher levels (119 ± 123 pg/mL) compared to AD. A significant positive correlation (r = +0.538, *p* ≤ 0.000) was also noted between plasma Aβ42 and p-tau. Our findings, although low in number of the patients, suggest that elevated plasma Aβ42 and p-tau may function as potential biomarkers for MCI and diagnostic indicators for DLB. Are we tackling enough wrong about β Amyloid? Prospective validation of multi-domain biomarkers beyond β Amyloid, in diverse populations of dementias is suggested.

**Keywords:** dementia; Alzheimer; beyond β Amyloid; beta-amyloid; p-tau

### 2.16. Beneficial Health Effects and Polyphenols of Greek Pomegranates

**Vasiliki S. Lagouri**

Laboratory of Chemistry-Biochemistry-Cosmetic Science, Department of Biomedical Sciences, University of West Attica, 12243 Egaleo, Greece

**Abstract: Background:** Pomegranates (*Punica granatum* L.), renowned for their antioxidant and anti-inflammatory properties, have garnered significant attention for their potential health benefits. This study aimed to investigate the antioxidant activity and bioactive compound profile of juice, peel, and seed extracts from pomegranate cultivars grown in mainland Greece (Central Macedonia and Thrace). **Methods:** Antioxidant capacity was assessed using DPPH and FRAP assays, while total phenols, flavonoids, hydrolysable tannins, and ellagic acid content were determined using UV and HPLC analysis. **Results:** Pomegranate peel extracts exhibited significantly higher antioxidant activity and total phenolic content compared to juice extracts. Moreover, peel extracts displayed significantly higher levels of flavonoids, hydrolysable tannins, and ellagic acid. These findings highlight the substantial antioxidant potential of pomegranate peels, often discarded as waste during juice processing. **Conclusions:** These results suggest that pomegranate peels could serve as a valuable source of natural antioxidants and bioactive compounds for various applications in the food, pharmaceutical, and nutraceutical industries. It could be used as a future therapeutic agent towards several vascular and neurodegenerative disorders such as hypertension, coronary heart disease and Alzheimer.

**Keywords:** phytochemicals; pomegranate; polyphenols; waste products; antioxidant activity

### 2.17. Development of LMWH Derivatives with Lower Anticoagulant Activity: A Potential Heparan Sulfate Regulator in COVID-19

**Fereshteh Sedaghat ^1,2^**

^1^  Aristotle University of Thessaloniki, School of Medicine, AHEPA Hospital
^2^  Outpatient Memory Clinic, Mashhad

**Abstract: Background**: The ongoing COVID-19 pandemic has highlighted a significant increase in neuropsychological issues among patients, necessitating further investigation into underlying mechanisms. One critical area of exploration involves the proteoglycan heparan sulfate (HS), found in both the extracellular matrix and on cell surfaces. HS plays a pivotal role in various biological processes at the molecular level, particularly in regulating neuroinflammation, synaptic development, and modulating immune responses. Research indicates that heparanase, an enzyme responsible for HS degradation, is elevated in COVID-19 patients, contributing to complications across multiple organ systems, including the brain. **Methods:** In our study, low molecular weight heparin (LMWH) was administered to three groups: (A) ten volunteers as preventive measures, (B) fifty-one COVID-19 patients across different disease stages, and (C) eighteen patients experiencing severe side effects. **Results:** Results showed that LMWH significantly improved outcomes in group A, leading to milder disease, while group B experienced faster symptom relief. In group C, substantial improvements in side effects were noted. These findings suggest that early administration of LMWH could regulate heparan sulfate dysregulation, mitigating COVID-19 symptoms and aiding recovery and prevention.

**Keywords:** COVID-19; Low molecular weight heparin; LMWH; treatment; prevention; heparan-sulfate

### 2.18. The Elephant That Is Still Kept in the Dark: COVID-19 and Vaccinations

**Fereshteh Sedaghat ^1,2^**

^1^  Aristotle University of Thessaloniki, School of Medicine, AHEPA hospital
^2^  Outpatient Memory Clinic, Mashhad

**Abstract: Background**: We stand at the crossroads of science, policy, and public trust. The global response to COVID-19 has profoundly transformed our approach to public health, but it has also revealed vulnerabilities in how we understand and communicate complex information. Here, we will try to explore key aspects of this challenging terrain: the dynamics of COVID-19 and vaccinations, and the barriers to open dialogue. COVID-19 and long VAX continue to linger in our lives, both through its direct impacts and the lasting effects it leaves its wake. **Results:** Socrates’ dialectical method teaches us that truth emerges from thoughtful questioning and dialogue. In the context of COVID-19, this method can help dissect complex issues, promoting deeper understanding rather than blind adherence to polarized opinions. Should we have treated this complex syndrome more as a toxic exposure rather than merely a viral infection? By breaking free from our traditional frameworks, we may better understand and address the ongoing challenges posed by COVID-19.

**Keywords:** COVID-19; intoxication; sulfur mustard; Socratic dialectal method; prevention; covid vaccinations

### 2.19. The Combined Use of Electroencephalography and Cerebrospinal Fluid Analysis in the Diagnosis and Prognosis of Alzheimer’s Disease: A Scoping Review

**Charikleia Karastamati**

Department of Brain and Behavioral Sciences, University of Pavia, 27100 Pavia, Italy

**Abstract: Background:** Alzheimer’s disease is a progressive neurodegenerative disorder that affects memory and other cognitive functions, making early diagnosis critical for intervention. The literature suggests an association between electroencephalography (EEG) and specific biomarkers of cerebrospinal fluid (CSF), reinforcing the need for further research. **Methods:** This review was conducted by analyzing 12 articles that examined this association, focusing on biomarkers such as β-amyloid (Aβ-42), total tau (t-tau), and phosphorylated tau (p-tau) proteins. **Results:** According to the findings, reduced levels of Aβ-42 are associated with increased power at slow frequencies (delta and theta) and reduced synchrony at faster frequencies (alpha and beta). In contrast, increased levels of t-tau and p-tau proteins are associated with reduced power and synchrony at the faster frequencies, especially in patients with mild cognitive impairment and Alzheimer’s disease. **Conclusions**: EEG is a non-invasive approach that provides valuable information for understanding the disease. Integrating EEG with CSF biomarker analysis seems promising for clinical practice.

**Keywords:** Alzheimer’s disease; EEG; cerebrospinal fluid; biomarkers

### 2.20. Predicting Dementia Onset and Progression from Longitudinal Neuroimaging Data

**Athanasia Kalogirou ^1^, Ioannis Ntzoufras ^1^ and John Kornak ^2^**

^1^  Statistics Department, Athens University of Economics and Business
^2^  Department of Epidemiology and Biostatistics, University of California, San Francisco

**Abstract: Background:** Dementia is a devastating form of disease with high heterogeneity. This variation is due to different underlying disease types as well as sub-types and disease presentations, leading to variation in patients’ experience and prognoses. The heterogeneity in dementia symptoms is further influenced by factors such as an individual’s overall health, as well as the brain regions most affected. Even within the same type of dementia, there is inter-individual variability in the rate of disease progression and symptoms. Understanding this heterogeneity is crucial for accurate diagnosis and disease course prediction. In particular, as potential new treatments are appearing on the market with many more in development, predicting cognitive impairment and decline is vital for optimizing patient selection in clinical trials and planning personalized treatment strategies. **Methods**: Our research used brain imaging structural measures data (regional volumes) from dementia patients (the ALLFTD study) and at-risk individuals. It explored Bayesian Variable Selection methods for predicting the presence and severity of cognitive impairment. Machine Learning methods were also explored for comparison and reproducibility. **Results-Conclusions**: The frontal and temporal lobes were identified as key regions for degeneration onset. The most promising models combined predictive ability (AUC 91%) with clinical interpretability, which is essential for medical practice adoption.

**Keywords:** Bayesian Variable Selection; Longitudinal Neuroimaging Data; Dementia Prediction
**Funding:** ALLFTD Consortium.

### 2.21. Pharmacochemical In Silico Study of Chalcones MAO Inhibitors as Multifunctional Molecules for the Treatment of Depression and Neurodegenerations

**Emmanouela Basgiouraki and Dimitra Hadjipavlou-Litina**

Department of Pharmaceutical Chemistry, School of Pharmacy, Aristotle University of Thessaloniki

**Abstract:** Depression and neurodegenerative diseases are pathological conditions that affect a large percentage of population, and their origin is multifactorial, including genetic predisposition, oxidative stress, environmental factors, etc. However, monoamine oxidases (MAOs), which are responsible for the metabolic oxidation of the neurotransmitters serotonin, dopamine, norepinephrine, etc., resulting in the deregulation of these levels, the production of further active oxidizing molecules, mitochondrial damage and ultimately neuronal death and neurodegeneration, play an important role in their pathophysiology. Therefore, it is considered necessary to study and develop new therapeutic molecules that act as MAO inhibitors, with a pleiotropic profile that leads to the inhibition of other enzymes or factors involved in depression and neurodegenerative diseases, with optimal action and fewer side effects, as cyclooxygenase and lipoxygenase (involved in inflammation), β secretase (involved in the production of amyloid β) and acetylcholinesterase (which breaks down the neurotransmitter acetylcholine). This study focuses on the investigation of the physicochemical and pharmacokinetic properties, the in vivo metabolic pathway, toxicity, and pharmacological similarity, using in silico protocols, of small chemical compounds with inhibitory action on MAOs, such as chalcones, which have demonstrated a broad spectrum of pharmacological effects. Due to their pleiotropic actions, they can be used for the treatment of neurodegenerative diseases.

**Keywords:** pharmacochemistry in silico models MAOs neurodegenerative diseases

### 2.22. Effects of Physical Exercise Environment and Protocol Intensity FITT for Patients with Neurodegenerative Diseases

**Vasiliki Garopoulou ^1^, Christos Mouzakidis ^2^, Evangelos Portelanos ^3^, Odysseas Kapousizis ^4^ and Magdalini Tsolaki ^5^**

^1^  Medical school, Aristotle University of Thessaloniki, A.U.Th. Greece Panhellenic Institute of Neurodegenerative Diseases Thessaloniki, Greece Hellenic Association of Alzheimer’s Disease and Related Disorders (Alzheimer Hellas)
^2^  Hellenic Association of Alzheimer’s Disease and Related Disorders (Alzheimer Hellas)
^3^  “Neuroscience and Neurodegeneration” M.Sc. Medical School A.U.Th. Greece
^4^  Panhellenic Institute of Neurodegenerative Diseases Thessaloniki School of Chemistry, A.U.Th
^5^  Medical school, Aristotle University of Thessaloniki, Thessaloniki, Greece Department of Classics Neuropsychiatrist, Lab of Neurodegenerative Diseases, CIRI, A.U.Th.

**Abstract:** The aging process is associated with a progressive loss of physiological and psychological functions that lead to inevitable disabilities that contribute to functional dependency and consequently in loss of quality of life (QoL). In this sense, changes in cognitive abilities are very common, contributing to the increase in the incidence of Neurodegenerative Diseases (ND). **Background**: ND can have a significant impact on patients cognitive, functional, and psychological status, as well as their quality of life. Exercise has been shown to have numerous benefits for patients with neurological diseases, (Alzheimer’s disease, Parkinson’s disease, Multiple Sclerosis, Dementia, Mild Cognitive Impairment (MCI), Polyneuropathies, etc.) including improved physical function, cognition, and quality of life. Furthermore, it is known that all health-related components of physical fitness seem to be compromised in these individuals. Specifically, significant reductions in cardiorespiratory and muscular endurance, muscular strength, and flexibility and in body composition have been observed. In addition, there is evidence regarding the benefits of physical activity on the functionality, cognition, psychology, and quality of life in older adults-patients with ND. The purpose of this study was to investigate the effects of a therapeutic exercise intervention using a therapeutic protocol that combines aerobic and anaerobic exercise with resistance training on functional outcomes, cognitive performance, QoL, among older adult individuals with ND. **Methods:** The total of participants (n = 27) with ND who were aged 59.15 and 64.21 years are all affected by mild or moderate disability included in this experimental study. Controlled experimental interventions are developed by forming a group of people with moderate dementia (n = 3), Alzheimer’s disease (n = 3), Parkinson disease (n = 5), Multiple Sclerosis (n = 14), and other types of neurological disorders (n = 2). A multimodal therapeutic exercise protocol intensity FITT consisted of twice weekly sessions (60 min each) over 20 weeks. The measurement tools: BMI, BSA, MMSE, Senior Fitness Test, ABC, T25-FW, BBS, FSS, EDSS, EQ-5D, SF-12, HADS-7, MSQOL-54 were evaluated before and after the intervention. The functional outcomes and cognitive performance measures were assessed before the beginning of the intervention and at the end of the intervention. To avoid learning effects, the participants were not well-known with the tests that were selected. We believe that the preliminary findings could encourage future investigations to elucidate the precise exercise effects in older adults. The limitations of this study include short intervention time and small sample size of patients with each disease. Furthermore, studies with a robust sample can help verify other issues within this theme including the influence of gender. **Results:** Our study demonstrated that an 8-week multimodal physical activity program is an important strategy to attenuate and/or ameliorate the decline of functional status (such as functional capacity, mobility, balance) and QoL of older adults. In addition, this intervention was able to promote cognitive improvement, which seems to be partially related with a therapeutic exercise protocol. **Conclusions:** We believe that the preliminary findings could encourage future investigations to elucidate the precise exercise effects in older adults.

**Keywords:** exercise; neurodegenerative diseases; quality of life; FITT

### 2.23. Healthy and Active Lifestyles Through the Cold-Water Swimming Link to Depression and Dementia

**Alexandros Oikonomou, Psychologist, Msc** in Mental Health-Medical School of Athens, President of the M.B./ENALMH Network

**Abstract: Background:** Explore a solution for both dementia and depression that’s social, invigorating, and accessible. Our project explores the potential of cold-water swimming, combined with breathwork and mindfulness, to tackle these growing health concerns. This innovative approach not only promotes inclusion by engaging isolated adults in a refreshing outdoor activity, but also champions a healthy lifestyle. Research suggests cold-water exposure may improve mood, alleviate depression, and even slow dementia progression. Also believe swimming is a valuable tool for promoting healthy lifestyles, reducing stress, and building self-esteem, challenge, inspire, and empower the participants to develop leadership skills and personal capacity; enhance their employability and entrepreneurship; and pursue a healthy lifestyle. Link to Depression and Dementia: Although research is still in its early stages, there is evidence that exposure to cold may have positive effects on people with depression and dementia.

**Keywords:** mindfulness; physical activity; cold water; dementia

**Funding:** COL.D.D. Project (colddproject.com), PROJECT NUMB 101133938/E.U.

### 2.24. Sound Minds: AI Speech Analysis for Cognitive Domain Assessment

**Fotiana Zouvani and Saturnino Luz**

University of Edinburgh

**Abstract: Background**: This study investigates the relationship between speech features and cognitive performance across different domains, particularly focusing on individuals with Mild Cognitive Impairment (MCI). This research explores how specific acoustic and linguistic speech features correlate with cognitive abilities, aiming to enhance early detection methods for cognitive decline. Connected speech of 82 participants (51 MCI and 31 neurotypical) from the Delaware corpus, and corresponding MoCA scores broken down by tasks reflecting different cognitive domains were analysed. **Methods:** Machine learning models were trained to predict MoCA scores and individual task performance based on extracted speech features. A logistic regression model trained on acoustic features achieved 98% sensitivity and 97.2% specificity in distinguishing MCI from unimpaired cognition. A random forest regressor trained on acoustic features had a mean absolute error of 1.188. **Results:** Post-hoc results indicate that acoustic features, such as pitch and loudness variations, were particularly effective in distinguishing patients with MCI from healthy controls and predicting performance in attention, memory, and executive function tasks. **Conclusions:** These findings contribute to the growing body of research supporting speech analysis as a non-invasive, cost-effective tool for cognitive health assessment, with potential applications in early detection and monitoring of cognitive disorders.

**Keywords:** AI; MCI; speech; cognitive decline; speech analysis; acoustics; linguistics

### 2.25. Using 3D-Printed Tools as Cognitive Enhancement Strategies in Dementia

**George Merdzhanov**

Social Store Initiative, Bulgaria, Alzheimer’s Bulgaria Association

**Abstract: Background:** Cognitive enhancement strategies involve engaging activities that stimulate thinking, concentration, and memory, serving as preventative measures against dementia. As technological advancements drive interdisciplinary approaches to combat age-related cognitive decline, integrating cognitive training with cutting-edge technologies becomes increasingly relevant. **Methods:** This study presents a qualitative assessment of an innovative program that integrates 3D printing technology to create cognitive tools, games, and training activities tailored for individuals affected by dementia. The project was implemented within the social enterprise ‘Social Store’ and included a structured guideline for caregivers on utilizing 3D-printed tools. The program aimed to enhance key cognitive domains such as memory, language, reasoning, creativity, and social engagement. In a follow-up phase, user experiences and the program’s impact on cognitive functioning and quality of life will be evaluated. **Results:** Preliminary findings suggest that the ‘Social Store’ initiative in the context of the development of cognitive activities has a positive effect on memory, emotional regulation, and overall quality of life for individuals with dementia and their caregivers. Feedback from participants highlighted the engaging and stimulating nature of the cognitive games, particularly in maintaining fine motor skills and social connectivity. Residents reported increased motivation and enjoyment from activities such as mosaics and the tree-leaf attachment game. While initial hesitation was observed, continuous participation led to growing confidence and active involvement. The ability to adapt these games to different cognitive levels made them accessible and effective across varying stages of dementia. Some minor challenges were identified, such as the need for game modifications to prevent frustration among participants. **Conclusions:** The integration of 3D printing technology in cognitive training programs presents a novel and scalable approach to dementia prevention. Future studies will explore the long-term cognitive and social benefits of these interventions, as well as potential improvements based on user feedback.

**Keywords:** cognitive enhancement; 3D printing; dementia prevention; cognitive decline

### 2.26. Support of Elderly People with Cognitive Impairment by Companion Robots

**George Siavalas and Vassilis Kaburlasos**

Department of Informatics, Computer and Telecommunications Engineering, School of Engineering, International Hellenic University (IHU), Greece

**Abstract: Background**: The demographic problem of Greece is characterized by a decrease in the general population and, at the same time, a significant increase in the percentage of the elderly (i.e., above 65 years) as well as a decrease in the percentage of children/teenagers. The demographic problem raises the critical issue of supporting the elderly because soon there will not be enough young people to care for all the elderly. One solution is to import human labor from abroad. As an alternative, short-term/medium-term solution, Companion Robots (COROs) are proposed here—COROs are understood as tools supporting the work of human-caregivers, who, although irreplaceable, are few to respond to all calls at all hours. This paper addresses the technological maturity for developing COROs to support the elderly. A CORO is necessary to have a physical body so as to be able to exchange physical objects with an elderly person e.g., to give him/her a glass of water etc. COROs that already exist on the market and have the physical body required to meet an elderly support role will be presented. Suffices a CORO be equipped with effective Artificial Intelligence (AI) algorithms. **Methods:** Despite the rapid advances of recent years, current AI seems to be based exclusively on statistical models of numerical data processing, whereas a human can, in addition, handle symbols, which current AI cannot. A scientific overview outlines the prevailing, Pythagorean notion that “numbers is the ultimate reality”. This work proposes overcoming the aforementioned problems by rigorously “computing with semantics” in a mathematical lattice data domain, where semantics is represented by a partial order relation. **Results:** Preliminary computational experiments demonstrate the application of an Intelligent Agent Model, or IAM for short, to be mounted on a CORO, for abductive reasoning. The proposed IAM algorithm enables a conversational intervention involving a CORO and an elderly. In particular, the CORO displays non-annotated images on a tablet and, when an elderly provides a false positive/negative response then the CORO both returns the right answer and, furthermore, it substantiates it. **Conclusions:** Preliminary results have been encouraging. Potential extensions of the proposed methods are discussed.

**Keywords:** companion robots; economic development; Greece’s demographic problem

**Funding:** This work was supported by the project “Artificial Intelligence and Applications” (IHU project code: 81960).

### 2.27. Facial Emotion Recognition Using Machine Learning Aiming at Improving the Life of Elderly Suffering from Dementia

**Christos Bakos ^1^ and Magdalini Tsolaki ^2^**

^1^  Anatolia College High School, Thessaloniki Greece
^2^  Professor of Aristotle University of Thessaloniki (AUTH), Thessaloniki, Greece

**Abstract: Background:** Music recommendation systems, which incorporate human-composed songs, were developed to enhance the quality of life for the elderly. However, these systems are very difficult to be applied to elderly individuals suffering from dementia. **Methods:** To address this issue, a closed-loop Person-to-Machine (P2M) system without the instructor’s help is proposed. This is achieved using a combination of Affecting Computing and Algorithmic Music and specifically by adjusting the algorithmic music parameters according to the facial responses of the dementia patients. The adjusted music parameters are namely: tempo, scale and instrument. By understanding the patient’s emotional state, the proposed system can then respond appropriately by changing the algorithmic music being played to influence the patient’s emotional state positively. This is achieved by reinforcement learning, where the system learns its interactions with the environment to maximize a cumulative reward. **Conclusions:** To the best of our knowledge, the proposed system is innovative and its advantage over the conventional systems is its ability to be applied in cases where the development of human-made personalized music lists, based on questionnaires and personal real-time interaction with patients, proved ineffective.

**Keywords:** machine learning; affecting computing; algorithmic music; dementia

### 2.28. Effects of Immersive VR Reminiscence Therapy on Cognition, Quality of Life, and Depression in Elderly with Cognitive Impairment and Dementia

**Raquel Simões de Almeida ^1^, Tiago Coelho ^1^, Álvaro Ribeiro ^2^, Pedro Dias ^2^, Maria Menezes ^2^ and Paula Portugal ^1^**

^1^  Psychosocial Rehabilitation Laboratory, Center for Rehabilitation Research, E2S, Polytechnic of Porto
^2^  E2S, Polytechnic of Porto

**Abstract: Background**: This study aimed to evaluate the effects of an immersive VR-based reminiscence therapy program on cognition, quality of life, and depression in elderly individuals with cognitive impairment and dementia, compared to a non-immersive reminiscence therapy program. **Methods:** A total of 19 participants were randomly assigned to three groups: an experimental group (immersive VR reminiscence therapy), an active control group (reminiscence therapy using 360° videos on a computer monitor), and a passive control group without intervention. Each participant completed 12 biweekly sessions. Outcomes were assessed using the Montreal Cognitive Assessment, the Quality of Life Scale—Alzheimer’s Disease, and the 15-item Geriatric Depression Scale. **Results:** Participants had a mean age of 80.6 years, predominantly female (84.2%) and married (42.1%). No significant differences were found between the groups in sociodemographic variables, except for age (*p* = 0.02). In terms of cognitive function, quality of life, and depression, no significant improvements were observed across any of the groups (*p* > 0.05), with a slight decline in some cases. **Conclusions:** Despite the evidence supporting reminiscence therapy’s benefits, this study did not observe significant gains in cognition, quality of life, or depression. The immersive VR intervention did not provide additional advantages. Further research is warranted.

**Keywords:** virtual reality; reminiscence therapy; cognitive impairment; dementia

### 2.29. Impulsivity, Inhibitory Control and Emotion Regulation in Older Adults

**Amaryllis-Chryssi Malegiannaki ^1^, Panagiota Rouzou ^1^ and Magdalini Tsolaki ^2,3^**

^1^  Department of Psychology, University of Western Macedonia
^2^  Greek Alzheimer Association and Related Disorders
^3^  Medical School, Aristotle University of Thessaloniki

**Abstract: Background**: Frontal lobe functions, including executive control and emotional regulation, are essential across the lifespan but may decline with age. This study investigated the impact of aging and educational level on cognitive and behavioral impulsivity, as well as emotion regulation in older adults, and explored the relationships between these functions. **Methods:** Fifty-four cognitively healthy older women (MMSE ≥ 27), divided into two age groups (60–70 and 71–90 years), were recruited from Day Centers in Thessaloniki. Participants completed the Barratt Impulsiveness Scale (BIS-11) to assess impulsivity, the Trail Making Test Part B (TMT-B) to evaluate inhibitory control, and the Difficulties in Emotion Regulation Scale (DERS). **Results:** The findings revealed no significant effects of age or education on inhibitory control, impulsivity, or emotion regulation. These results may be attributed to the participants’ high educational levels and engagement in cognitive enhancement activities. Impulsivity emerged as a stable trait, unaffected by age, while performance on the inhibitory control task was negatively correlated with motor impulsivity. Moreover, subscales of impulsivity were associated with difficulties in emotion regulation. **Conclusions:** These results emphasize the stability of certain cognitive and emotional traits in healthy older adults and provide a foundation for future research on multifactorial models of these relationships.

**Keywords:** ageing; impulsivity; executive control; emotion regulation

### 2.30. Τhe Education of Healthcare Professionals Through Telemedicine

**Magda Tsolaki**

^1^  Neurologist/Psychiatrist & Theologist, GAARD, Palliative Care Unit “Panagia Glykofilousa” Thessaloniki, Greece
^2^  1st Department of Neurology, Aristotle University of Thessaloniki
^3^  Alzheimer Hellas, Thessaloniki, Makedonia, Greece
^4^  Laboratory of Neurodegenerative Diseases, Center for Interdisciplinary Research and Innovation (CIRI-AUTh), Balkan Center, Aristotle University of Thessaloniki, Thessaloniki, Makedonia, Greece

**Abstract:** The first educational program face to face in Greece started in 2000 when Alzheimer Hellas had the suitable to room in “Chariseio” Old Age Nursing Home to start training. Patients, caregivers but also healthcare professionals (HCP) had the opportunity to attend this program. Later on, we had the opportunity to have a funding from ADI and Stavros Niarchos Foundation to train 40 HCPs from different cities all over Greece but also it was face to face. The first time we had a training program virtually for HCP but also for caregivers was in 2010 when we received a funding for the program ASPAD from the program “ARISTEIA” of the Ministry of Education. Later on, we started a collaboration between Alzheimer Athens and Alzheimer Hellas to train HCPs in different Centers of Health under the Umbrella of the Greek Inter-Municipal Network of Healthy Cities which was finished. Finally in 2023 Alzheimer Athens and Alzheimer Hellas started a collaboration every second Monday of the month and their HCPs present the programs they are doing at their day centers for patients and caregivers. During this period from 2000 until now we organized 1st National Conference on Alzheimer’s disease and related disorders, 2000, 2nd National Conference on Alzheimer’s disease and related disorders, 2002 3rd National Conference on Alzheimer’s disease and related disorders and Alzheimer Europe Conference, 2003, 9th National Conference on Alzheimer’s disease and related disorders and 1st Mediterranean Conference on Neurorodegenerative Diseases, 2009, 10th National Conference on Alzheimer’s disease and related disorders and ADI Conference, 2010 and finally, 14th National Conference on Alzheimer’s Disease and Related disorders and 6th Mediterranean Conference on Neurorodegenerative Diseases, 2025. Since 2021 our conferences were either only virtual or mixed. The programs of all these Conferences and training programs are on the site of Greek Federation of Alzheimer’s Disease: www.alzheimer-federation.gr (accessed on 10 February 2025).

**Keywords:** training and educational programs; Greek Alzheimer Federation

### 2.31. Technology Experience and Mobile Device Proficiency Change According to Cognitive Status in Older Adults with Subjective Cognitive Decline and Mild Cognitive Impairment

**Alexander Moreno ^1,2,3^, Thanos Chatzikostopoulos ^4,5^, Ioannis Spantidakis ^6^, Mégan Dubois ^1,2^, Célia Rigoulat ^2,7^, Eugénie Côté ^1,2^, Adriana Dieumen ^1,2^, Pénélope Pelletier ^1,2^ and CIMA-Q ^8^**

^1^  Department of Psychology, Université de Montréal, Montreal, Quebec, Canada
^2^  Centre de recherche de l’Institut universitaire de gériatrie de Montréal (CRIUGM)–Innovation, Technology, and Cognition (INTECOG) Laboratory, CIUSSS du Centre-Sud-de-l’Île-de-Montréal, Montreal, Canada
^3^  Notre-Dame Hospital, Centre intégré universitaire de santé et de services sociaux du Centre-Sud-de-l’Île-de-Montréal (CCSMTL), Montreal, Quebec, Canada
^4^  Aristotle University of Thessaloniki, Department of Psychology, Faculty of Philosophy, Thessaloniki, Greece
^5^  Greek Association of Alzheimer’s Disease and Related Disorders, Thessaloniki, Greece
^6^  Department of Psychology, Scientific College of Greece, Athens, Greece
^7^  Écoles Universitaires de Recherche (EUR) Healthy, Université Côte d’Azur, Nice, France
^8^  Consortium for the early identification of Alzheimer’s Disease–Quebec, Canada

**Abstract: Background:** The use of technologies including smartphones can help older adults to continue to live independently. However, little is known about technology experience and mobile device proficiency in individuals at risk for dementia. This study aimed to explore the technological experience and proficiency with mobile devices in a group of older adults from the CIMA-Q study (Consortium for the early identification of Alzheimer’s Disease–Quebec, Canada). **Methods:** A total of 232 older adults were recruited from the CIMA-Q study. The diagnosis was confirmed by a memory clinic or expert physicians. Cognitive functioning was measured with the Montreal Cognitive Assessment (MoCA), while other measures included the Technology Experience Profile and the Mobile Device Proficiency Questionnaire–Short Form. **Results:** MoCA scores are positively associated with technological experience and mobile device proficiency. Older adults with subjective or objective cognitive impairment report increasing difficulties with technology. Besides, they report increasing difficulties with mobile device basics and different smartphone functions. **Conclusions:** As older adults move through the continuum from normal cognition to subjective cognitive decline or mild cognitive impairment, they report lower levels of mobile device proficiency and technological experience. These results are important for the development of gerontechnologies adapted to the cognitive level of potential users.

**Keywords:** gerontechnology; AgeTech; technology experience; mobile devices; dementia; cognition; older Adults

**Funding:** This research was initially supported by FRQS-Pfizer, FRQ Cohorte, RQRV, CCNA, Fondation Courtois (Neuromod project) and Fondation Famille Lemaire. With financial assistance provided by the Natural Sciences and Engineering Research Council of Canada (NSERC), and Gouvernement du Québec (Ministère de l’Économie, de l’Innovation et de l’Énergie) in Quebec, Canada. Dr. Moreno is supported by an AGE-WELL-EPIC-AT Fellowship and the Réseau Québécois de Recherche sur le Vieillissement (RQRV), a Research Network financed by Fonds de recherche du Québec.

### 2.32. Improving End-of-Life Care with Virtual Reality: Co-Construction with Palliative Care Stakeholders and Developers in the Canadian Context

**Pénélope Pelletier ^1,2^, Eugénie Côté ^1,2^, Célia Rigoulat ^2,4^, Adriana Dieumen ^1,2^, Mégan Dubois ^1,2^ and Alexander Moreno ^1,2,4^**

^1^  Department of Psychology, Université de Montréal, Montreal, Quebec, Canada
^2^  Centre de recherche de l’Institut Universitaire de Gériatrie de Montreal (CRIUGM)–Innovation, Technology, and Cognition (INTECOG) Laboratory, CIUSSS du Centre-Sud-de-l’Île-de-Montréal, Montreal, Canada
^3^  Écoles Universitaires de Recherche (EUR) Healthy, Université Côte d’Azur, Nice, France
^4^  Notre-Dame Hospital, Centre intégré Universitaire de Santé et de Services Sociaux du Centre-Sud-de-l’Île-de-Montréal (CCSMTL), Montreal, Quebec, Canada

**Abstract: Background:** The positive effects of virtual reality (VR) in palliative care, and its usability, feasibility, and acceptability have been demonstrated. However, its use in palliative care remains largely unexplored in the province of Quebec, Canada. We aimed to conduct two feedback sessions with individuals working in the context of palliative care to cocreate and improve the VR content intended for end-of-life care. **Methods:** A single 10-min session created by technicians and a movie maker from the Montreal-based Start-Up « Nipper Media » (“Come with me”) during which participants explore relaxing environments with a VR headset. Two groups of individuals working in palliative care (Cohort 1: n = 12; Cohort 2: n = 20) responded to a questionnaire created by the research team to capture their experience and suggestions to improve the VR content (e.g., comfort, sound quality, cybersickness symptoms, and duration of the experience). **Results:** Two iterations led to improve the content with participants reporting less dizziness/headache (16.7% versus 10%) and increased comfort levels (58.3% versus 70%). **Conclusions:** The co-construction of VR content intended for end-of-life care with palliative care staff and developers is possible. Improvements in the quality of the content will make it more suitable for end users.

**Keywords:** virtual reality; palliative care; end-of-life; older adult; Canada; Quebec

**Funding:** With financial assistance provided by the Natural Sciences and Engineering Research Council of Canada (NSERC), and Gouvernement du Québec (Ministère de l’Économie, de l’Innovation et de l’Énergie) in Quebec, Canada. Dr. Moreno is supported by an AGE-WELL-EPIC-AT Fellowship and the Réseau Québécois de Recherche sur le Vieillissement (RQRV), a Research Network financed by Fonds de recherche du Québec.

### 2.33. How Has Virtual Reality Been Used in Palliative Care? Preliminary Evidence from a Systematic Review of the Literature

**Eugénie Côté ^1,2^,** **Iveta Fajnerová ^4^** **, Célia Rigoulat ^2,4^,** **Salima Belhouari ^1,2^** **, Adriana Dieumen ^1,2^, Pénélope Pelletier ^1,2^, Mégan Dubois ^1,2^,** **Zubková Anna ^5^** **, O’Connor Patrick ^2^ and Alexander Moreno ^1,2,4^**

^1^  Department of Psychology, Université de Montréal, Montreal, Quebec, Canada
^2^  Centre de recherche de l’Institut Universitaire de Gériatrie de Montréal (CRIUGM)–Innovation, Technology, and Cognition (INTECOG) Laboratory, CIUSSS du Centre-Sud-de-l’Île-de-Montréal, Montreal, Canada
^3^  Center for VR Research in Mental Health and Neuroscience, National Institute of mental Health, Klecany, Czech Republic
^4^  Écoles Universitaires de Recherche (EUR) Healthy, Université Côte d’Azur, Nice, France
^5^  Department of Psychiatry, Charles University, Prague, Czech Republic
^6^  Notre-Dame Hospital, Centre Intégré Universitaire de Santé et de Services Sociaux du Centre-Sud-de-l’Île-de-Montréal (CCSMTL), Montreal, Quebec, Canada

**Abstract: Background:** Virtual reality (VR) has shown its clinical potential to improve cognitive and psychological functioning in individuals with dementia. VR studies have been conducted with few adverse side effects, but more information is needed in the context of its use in palliative care. This study aimed to systematically review the scientific literature about VR use in palliative care, with an emphasis on older adults with dementia. **Methods:** The search was conducted in five databases (Embase, Web of Science, MEDLINE, CINAHL, and PsycINFO) (PROSPERO registration number CRD42024522413). We included (a) studies conducted in children, adults, or older adults, (b) any life-limiting disease requiring palliative care, (c) with family caregivers, individuals or staff in palliative care, healthcare professionals, (d) semi-immersive or immersive VR, (e) training or therapeutic purposes, (f) qualitative, quantitative, or mixed studies, and (g) conducted at home, in the laboratory, or a hospital setting. **Results:** Of the 1005 studies identified, 40 were retained for extraction following screening with COVIDENCE software. Five percent corresponded to pediatric VR interventions, 22.5% to adults, 55% to adults/seniors, 5% to seniors, and 12.5% were not specified. They mainly come from European countries (37.5%) or the USA (30%). **Conclusions:** The preliminary analysis of the information suggests a lack of studies using VR as an intervention for palliative care in older adults with dementia. There is an opportunity for the development of VR content for palliative care in individuals with neurodegenerative disorders.

**Keywords:** palliative care; virtual reality; end-of-life; technology; systematic review

**Funding:** With financial assistance provided by the Natural Sciences and Engineering Research Council of Canada (NSERC), and Gouvernement du Québec (Ministère de l’Économie, de l’Innovation et de l’Énergie) in Quebec, Canada. Dr. Moreno is supported by an AGE-WELL-EPIC-AT Fellowship and the Réseau Québécois de Recherche sur le Vieillissement (RQRV), a Research Network financed by Fonds de recherche du Québec.

### 2.34. Characteristics of a Socially Assistive Robot for Older Adults: Inputs from Healthcare Staff and Users

**Alexander Moreno ^1,2,3^, Reshma Rajendran ^4,5^, Nigel Harris ^4^, Carlos Cifuentes ^4^, Célia Rigoulat ^2,6^ and Marcela Múnera ^4^**

^1^  Department of Psychology, Université de Montréal, Montreal, Quebec, Canada
^2^  Centre de recherche de l’Institut universitaire de gériatrie de Montréal (CRIUGM)-Innovation, Technology, and Cognition (INTECOG) Laboratory, CIUSSS du Centre-Sud-de-l’Île-de-Montréal, Montreal, Canada
^3^  Notre-Dame Hospital, Centre intégré universitaire de santé et de services sociaux du Centre-Sud-de-l’Île-de-Montréal (CCSMTL), Montreal, Quebec, Canada
^4^  Bristol Robotics Laboratory, University of the West of England, Bristol, UK, University of Bristol, Bristol, UK
^5^  The Bath Institute for the Augmented Human, University of Bath, Bath, UK
^6^  Écoles Universitaires de Recherche (EUR) Healthy, Université Côte d’Azur, Nice, France

**Abstract: Background:** Global aging poses global economic challenges to institutions, professionals, and family caregivers. Age-related illnesses necessitate sustained resources as family dynamics change. Innovative solutions such as Social Assistive Robots (SARs) can enhance older adults’ well-being. This study aims to explore perceptions and needs regarding SARs among older adults, caregivers, and healthcare professionals, considering varying levels of technology experience. We focus on how different user profiles influence openness to SARs, ultimately guiding the design of a robot that is accessible, intuitive, and responsive to user preferences. Notably, this research was conducted without a robot prototype, enabling participants to express their expectations and concerns freely, without the influence of interacting with an existing model. **Methods:** This study integrates user-centered design principles into human-robot interaction frameworks, aligning with the population’s preferences. We used a custom-made questionnaire with standardized scales, including the Technology Experience Profile, Mobile Device Proficiency Questionnaire, Negative Attitudes towards Robots Scale, and the Technology-Specific Expectation Scale. We also conducted semi-structured interviews with older adults, caregivers, and healthcare professionals in care homes, incorporating a video showcasing different types of SARS to gather insights. **Results:** A Mann-Whitney U Test revealed that compared to participants with low levels of technology experience, perceptions of SARs were significantly more favourable in individuals with higher levels of technology experience (U = 73.5, *p* < 0.001, Cohen’s *d* = 1.15). Qualitative insights from participants highlight the importance of technology-focused solutions for diverse user profiles. **Conclusions:** Acknowledging and addressing the gap in technology experience is pivotal for optimising the design and acceptance of SARs, ensuring alignment with older adults’ preferences, and significantly enhancing their overall well-being.

**Keywords:** social assistive robots (SARs); user-centred design (UCD); human-robot interaction (HRI); elderly care

### 2.35. Financial Capacity, Biological Factors and Neurocognitive Disorders: From Bench to Bedside

**Vaitsa Giannouli**

School of Medicine, Aristotle University of Thessaloniki, Greece

**Abstract: Background**: Financial capacity assessment in older adults suffering from different types of neurocognitive disorders is a topic of debate, given the economic, medical, legal, and ethical implications for the older persons, their families and our modern societies. **Methods:** This review examines the role of biological factors (e.g., APOE genes, cerebrospinal fluid biochemical markers, brain volumes etc.) on financial capacity performance. **Results:** Results highlight the importance of vascular factors (e.g., elevated blood pressure) and volumes of brain regions (in particular the left angular gyrus and the amygdala). On the other hand, biological factors such as the Apoe e4 gene, the existence of phosphorylated tau (pTau), a protein that plays a critical role in the development of Alzheimer’s disease, as well as beta amyloid protein peptides (Aβ42), the main component of amyloid plaques do not influence the performance of older individuals in relevant financial tasks such as the Legal Capacity for Property Law Transactions Assessment Scale (LCPLTAS). **Conclusions:** The goal of this review is to propose the inclusion of the abovementioned important biological variables in future assessment protocols of financial capacity.

**Keywords:** neurocognitive disorders; financial capacity; assessment; biological risk factors

### 2.36. A Randomized Clinical Trial of Phenolic Compounds from Olives (Olea europaea L.) to Verify Protection Against Progression to Dementia and Alzheimer’s in Mild Cognitive Impairment Patients

**Magda Tsolaki ^1,2,3^, Antonio Mele ^4^, Silvia Mele ^6^, Eleni E. Tzekaki ^3,5,†^, Georgios Katsipis ^3,5,†^, Anastasia Pantazaki ^3,5^ and Prokopios Magiatis ^7^**

^1^  1st Department of Neurology, Aristotle University of Thessaloniki
^2^  Alzheimer Hellas, Thessaloniki, Makedonia, Greece
^3^  Laboratory of Neurodegenerative Diseases, Center for Interdisciplinary Research and Innovation (CIRI-AUTh), Balkan Center, Aristotle University of Thessaloniki, Thessaloniki, Makedonia, Greece
^4^  Levius Vita Foods srl, Montecatini Terme, Italy
^5^  Laboratory of Biochemistry, Department of Chemistry, Aristotle University of Thessaloniki, 54124 Thessaloniki, Greece
^6^  School of Specialization in Hygiene and Preventive Medicine, University of Florence, Italy
^7^  Department of Pharmacognosy and Natural Products Chemistry, Faculty of Pharmacy, National and Kapodistrian University of Athens, Panepistimiopolis Zografou, Athens, Greece
^†^  Equally Contributing Authors.

**Abstract:** The results of the MICOIL Pilot Study opened the door to the possibility that High Phenolic (HP) EVOO could act as a protective compound to prevent MCI patients from progressing to AD. In their conclusions, the authors stated that it is necessary to perform future randomized studies with larger sample sizes, more biomarkers and longitudinal follow-up cognitive assessments to obtain stronger evidence on the role of EVOO’s polyphenols on cognition and especially for *APOE ε*4 carriers to see if the results of the MICOIL Pilot Study remain or improve and prevent the MCI patients with *APOE* ε4 from progressing to AD after five or more years. The authors pointed out also some limitations they found in carrying on the study, of which the most important was the relatively small sample size due to uncertainty about the availability of a sufficient volume of EVOO with very high polyphenol content. The aim of this clinical trial, in continuation of the MICOIL Pilot Study, is to study what was considered unfinished to get answers to the above-listed open issues and to find a way to overcome the limitations about the availability of a sufficient volume of (HP)EVOO. We have designed a Randomized Clinical Trial to verify this possibility using not EVOO but the natural water fraction of the same olives that have normally a concentration of polyphenols up to 20 times higher than EVOO. Before the intervention Aβ1-40 and Aβ1-42 species, total tau, and ptau in the CSF of participants will be determined, only for precise diagnosis. While before and after the intervention the well-established AD biomarker 217p-tau in the plasma of participants will be determined. The phenolic compound under investigation is a proprietary product named VITA P; it is the natural aqueous juice of olives concentrated simply by removing water via evaporation. The aqueous juice is obtained from olives (*Olea europaea* L.) of the Coratina variety from olive trees located in the Northern part of the Puglia Region, Italy. The concentration of polyphenols in VITA P is more than 200 times higher than in good-quality EVOOs.

The advantages of using the concentrated aqueous juice VITA P over the use of EVOO are:

- Substantially unlimited availability of the product and a very high shelf life (stability) compared to EVOO
- For the participants the product is much easier to be handled as it will be provided in dropper bottles containing 30–35 mL of product and equipped with a pipette with rubber bulb able to aspirate about 1 mL of liquid. The product can be immediately dissolved in water or another preferred juice by the participant,
- 10 drops of VITA P contain about 24 mg polyphenols, a quantity higher than total polyphenols in 50 mL HP-EH-EVOO used in the MICOIL pilot Study,
- 10 drops of VITA P have close to zero calories compared to about 450 Kcal of 50 mL HP-EH-EVOO

Study design: Participants will be randomised in four groups, each group of 50 participants.

The duration of the study will be 18 months.

The four groups include patients with MCI and each participant will be randomised and allocated to one of the four groups:

- Group 1 will receive VITA P about 40 mg polyphenols/day (16 drops/day) together with MeDi instructions.
- Group 2 will receive VITA P about 20 mg polyphenols/day (8 drops/day) together with MeDi instructions.
- Group 3 will receive placebo together with MeDi instructions.
- Group 4 will receive only MeDi instructions.

**Keywords:** RCT; phenolic compounds; mild cognitive impairment; prevention

### 2.37. Investigating the Potential of Yttrium Oxide Nanoparticles and Zinc Oxide Nanoparticles for Parkinson’s Disease Treatment

**Kim San Tang ^1^, Wesley Zhi Chung See ^2^, Hidayat Ullah Khan ^1^ and Rakesh Naidu ^2^**

^1^  School of Pharmacy, Monash University Malaysia, Jalan Lagoon Selatan, 47500 Bandar Sunway, Selangor, Malaysia
^2^  Jeffrey Cheah School of Medicine and Health Sciences, Monash University Malaysia, Jalan Lagoon Selatan, 47500 Bandar Sunway, Selangor, Malaysia

**Abstract: Background**: Parkinson’s disease (PD) is a debilitating neurodegenerative disorder characterized by impaired movement. Exposure to the herbicide paraquat has been linked to an increased risk of developing PD, with oxidative stress recognized as a key contributor to its pathogenesis. This study investigates the protective effects of zinc oxide nanoparticles (ZnO-NPs) and yttrium oxide nanoparticles (Y_2_O_3_-NPs) against paraquat-induced cell death in SH-SY5Y cells. **Methods:** Cells were treated with varying concentrations of ZnO-NPs (0.1–1.0 μg/mL) or Y_2_O_3_-NPs (1–10 μg/mL) for 72 h, with paraquat (300 μM) introduced after 24 h. Cell viability was assessed using the MTT assay, while oxidative stress markers, including reactive oxygen species (ROS), malondialdehyde (MDA), and superoxide dismutase (SOD) activity, were measured. Apoptosis was evaluated through a caspase-3/7 assay. **Results:** Results indicated that Y_2_O_3_-NPs did not protect against paraquat-induced cell death, while ZnO-NP treatment significantly enhanced cell viability. Although ZnO-NPs did not reduce ROS, MDA, or SOD activity, they significantly suppressed caspase-3/7 activity. **Conclusions:** ZnO-NPs exhibit anti-apoptotic potential, warranting further investigation into their neuroprotective mechanisms in PD.

**Keywords:** nanoparticles; paraquat; Parkinson’s disease; yttrium oxide; zinc oxide

**Funding:** The authors thank the Ministry of Higher Education, Malaysia, for supporting this study (Project code: FRGS/1/2020/SKK0/MUSM/03/5).

### 2.38. Satisfaction of Beneficiaries in Open Elderly Care Structures in the Community

**E.M. Foukaki ^1^ and S. Koukouli ^2^**

^1^  Social worker, MSc, PhD (c), Physical and Medical Rehabilitation Center of Rethymnon, Academic Scholar, Member of the Laboratory of Interdisciplinary Approaches for the Enhancement of Quality of Life (QoL lab), Department of Social Work, Hellenic Mediterranean University (HMU)
^2^  Associate Professor of Social Policy, Department of Social Work, Laboratory of Interdisciplinary Approaches for the Enhancement of Quality of Life (QoL lab), Institute of Agri-Food and Life Sciences, Hellenic Mediterranean University (HMU)

**Abstract: Background**: The concept of satisfaction among elderly beneficiaries is not relatively new. The interest of healthcare professionals is increasingly focused on issues concerning the satisfaction of service users with the provided healthcare services in an effort to address the growing needs of elderly individuals, either through the improvement of existing services or through the development and design of new community care services, by utilizing this data. In recent years, the public services sector has been actively researching user satisfaction, acknowledging its crucial role in formulating effective policies. Nevertheless, the field of public health significantly lags behind in research related to the satisfaction of service recipients. **Methods:** Through this work, the results of a study conducted as part of a doctoral dissertation will be presented. The purpose of the research was to assess the level of satisfaction of elderly beneficiaries in the services of K.A.P.H., K.H.F.H., and V.S.S. in the 23 municipalities of the Region of Crete. The sample was selected through stratified sampling, and data collection was carried out using a self-administered questionnaire that was constructed for the needs of the research. **Results:** A total of 406 elderly individuals participated in the study, of which 350 were active members of the Senior Citizens’ Centers, 65 were beneficiaries of the Home Care Services, and 285 were beneficiaries of the Social Welfare Services. **Conclusions:** Research in the field of beneficiary satisfaction with the provision of services in open care structures for the elderly presents several limitations and requires a very good understanding of the research subject. On the other hand, it can provide data necessary for the evaluation and, consequently, the improvement of the services provided.

**Keywords:** beneficiary satisfaction; elderly; open structures; KIFI; KAPI; VSS

### 2.39. Perceived Social Support and Burden Among Family Caregivers for People with Dementia

**Nikoletta Ntontorou**

Department of Psychology, University of Ioannina, 45110 Ioannina, Epirus, Greece

**Abstract: Background**: The burden on caregivers of people with dementia has adverse effects in most aspects of their lives, including health. International literature has mainly highlighted demographic and dementia-related factors as predictors of the reported burden. Considering the role of social support in managing stressful situations, this research aims to investigate the relationship between the burden of family caregivers with dementia and perceived social support. **Methods:** The study has a cross-sectional correlational design with quantitative data. The sample consists of family caregivers of people with dementia, and the research tools are: (a) the Zarit Burden Scale, (b) Multidimensional Scale of Perceived Social Support and (c) Questionnaire on demographic characteristics of caregivers and people receiving care, information on dementia and care. **Results:** The results will be discussed in the context of the current literature, aiming for a deeper understanding of the role of social support in the burden of family caregivers with dementia, as well as its contribution to the design of interventions to reduce the burden and improve the care. **Conclusions:** This study highlights the significant role of social support in reducing the burden on family caregivers of people with dementia, emphasizing the need for targeted interventions to improve well-being.

**Keywords:** social support; burden; family caregiver; dementia

**Funding:** This research received no funding.

### 2.40. Validation of a Tablet-Administered Culturally Oriented Screening Test for Mild and Major Neurocognitive Disorders

**A. Solias ^1^, V. Karagkounis ^2^, Th. Kallinikaki ^3^, Ch. Tsairidis ^4^ and M. Tsolaki ^5^**

^1^  Social Worker PhD candidate, Democritus University of Thrace
^2^  Professor. Dept of Social Work Democritus University of Thrace
^3^  Emeritus Professor. Dept of Social Work Democritus University of Thrace
^4^  Associate Professor. Dept of Social Work Democritus University of Thrace
^5^  Emeritus Professor of Neurology Aristotle University of Thessaloniki

**Abstract: Introduction:** Early detection is crucial for diagnosis and meta-diagnosis care, providing the opportunity to exploit the benefits of available drugs and non-pharmacological interventions. **Purpose:** The validation of a screening test based on a traditional legendary story. **Methods:** Cross-sectional study. The metric ability of the electronic version of the Hagia Sophia test (eHAST) is examined in a sample of 708 people 55+ years old, backed by Alzheimer’s Hellas Day Centers. All participants were detailed neuropsychologically assessed and clinically diagnosed. **Results:** Receiver operating characteristics curve (ROC) estimation of the eHAST’s screening value with clinical diagnosis as a gold standard, yielded a sensitivity of 0.955, specificity of 1.0 and area under the curve (AUC) of 1.0 for the discrimination of older adults with cognitive concerns (OACC) vs patients with dementia. Sensitivity 0.855, specificity 0.935 and AUC 0.958 for the discrimination of MCI vs patients with dementia. Finally, sensitivity 0.804, specificity 0.544 and AUC 0.733 for the discrimination OACC vs. MCI. The discriminant ability (cross-validated) of eHAST respectively for the aforementioned categories is 95.9%, 91.4% and 71.5%. **Conclusions:** eHAST has a robust ability to detect dementia and MCI. The development of alternative validated screening tests is a challenging task facing the expected outburst of dementia in middle- and low-income populations.

**Keywords:** screening; dementia; MCI

### 2.41. Triggering of Alzheimer’s Disease by Microorganisms

**T. Moysidou and E. Andreadou**

Department of Biomedical Sciences, School of Health Sciences, International Hellenic University, Sindos, Thessaloniki, Greece

**Abstract:** Alzheimer’s Disease (AD) affects the daily lives of millions of people worldwide. Regarding its pathogenesis, although the prevailing theories focus on the β-amyloid peptide and tau protein, scientists increasingly highlight the involvement of microorganisms in triggering the disease. Current literature emphasizes the role of bacteria and their metabolites, both through dysbiosis of the gut and oral microbiome and through the invasion of microorganisms into the central nervous system via various pathways. This invasion occurs through the olfactory system, the oral cavity, the gut-brain axis, the skin, and the disruption of brain barriers. Emerging evidence also supports the involvement of viruses and fungi. Infections that increase the risk of developing AD constitute a modifiable risk factor that can be addressed. The identification of appropriate bacterial biomarkers, which are either structural or functional components of bacteria, may provide an innovative approach for the early detection and monitoring of disease progression enhancing the potential for more effective management in the future. This study aims to highlight the possible mechanisms through which microorganisms trigger neurodegenerative diseases while also focusing on the biomarkers associated with these pathogens.

**Keywords:** Alzheimer’s disease; microbiome; dysbiosis; bacterial biomarkers

### 2.42. Taste and Olfactory Dysfunctions on Parkinson’s and Alzheimer’s Diseases

**Ioannis Bourakis ^1,2^, Thomas Tegos ^2^, Anastasia Konsta ^3^, Marina Makri ^2,4^ and Magdalini Tsolaki ^2,4^**

^1^  Department of Occupational Therapy, Faculty of Health Sciences, University of Western Macedonia, Ptolemaida, Greece
^2^  Department of Neurology, School of Medicine, Faculty of Health Sciences, Aristotle University of Thessaloniki, Thessaloniki, Greece
^3^  Department of Psychiatry, School of Medicine, Faculty of Health Sciences, Aristotle University of Thessaloniki, Thessaloniki, Greece
^4^  Greek Association of Alzheimer’s Disease and Related Disorders, Thessaloniki, Greece

**Abstract: Background:** Taste and olfactory dysfunction can be useful tools for the early diagnosis of Alzheimer’s (AD) and Parkinson’s (PD) diseases. **Objective:** The present study aims to investigate the apparent correlation between olfactory and gustatory impairment and the clinical signs of AD and PD, through a systematic review of the current literature. **Methods:** A systematic review was conducted in the Medline (PubMed) and Cochrane Central Register of Controlled Trials (Central) databases from January 2020 to February 2023. Longitudinal clinical trials conducted in people with either AD or PD as well as simultaneously examined for olfactory and/or gustatory impairment. **Results:** Among 7760 articles identified, ten longitudinal research studies met the clearly defined criteria and were submitted to extract specific information. **Conclusions:** The sense of smell and taste appeared to decrease to a maximum extent several years before the onset of symptoms of AD and PD. It is of utmost importance that more experimental research is needed to record the thresholds of smell and taste in both healthy elderly and people with AD and PD.

**Keywords:** hyposmia; anosmia; ageusia; hypogeusia; Parkinson’s disease; Alzheimer’s disease

### 2.43. Multiple Sclerosis and Legal Capacity

**Panagiota Voskou ^1,2^**

^1^  University of Athens
^2^  Neurologist, MSc, PhD, Post-doc Cand.

**Abstract: Background**: Cognitive deficits may occur in about 60% of patients with multiple sclerosis (MS), while executive functions, working memory and new-learning are commonly impaired. Nevertheless, there is limited research regarding decisional capacity in MS. **Methods**: A literature review in the PubMed database has been made. **Results**: Overall financial capacity (FC) and more complex financial domains, i.e., managing bank accounts, are significantly impaired in progressive MS, with mental flexibility and working memory mostly involved. Short-term verbal memory and basic arithmetic ability are major neurocognitive predictors of FC in progressive MS. Ability to understand treatment and clinical research is commonly impaired in MS, i.e., misunderstanding treatment details due to diminished new-learning or executive functions or difficulty in comprehending treatment risks and benefits. Recognition cueing can be helpful for robust informed consent from MS patients. Depressive distress is also common in MS, and this corresponds with poor reasoning and, subsequently, decisional incapacity. **Conclusions**: More studies should be conducted about the capacity for informed consent and financial tasks in MS, as well as which neurocognitive functions are related to specific components of medical decision-making. The primary goal should be the protection of rights and autonomy of patients with MS. Depression and its neurocognitive correlates in MS should be also examined in future research.

**Keywords:** multiple sclerosis; legal capacity; decision-making

### 2.44. Exploring for Correlations Between Sociodemographic Characteristics with Anxiety and Depressive Symptomatology in Elders

**A. Solias ^1^, Ch. Tsairidis ^2^, N. Degleris ^3^ and M. Tsolaki ^4^**

^1^  Social Worker Municipality of Ilion, PhDc, Democritus University of Thrace
^2^  Associate Professor. Dept of Social Work Democritus University of Thrace
^3^  Psychiatrist, Head NeuroScience-Dialectical Behavioral Therapy Mental Health Prevention Unit University of Piraeus
^4^  Emeritus Professor of Neurology Aristotle University of Thessaloniki

**Abstract:** Anxiety and depressive disorders are common in elders as independent clinical entities as well as in their mixed form. Purpose: to investigate associations between anxiety and/or depressive symptomatology with:

- Sociodemographic factors, stressful life events in childhood, adolescence and adulthood
- The phenomenology as it is reflected in the items of the scales (GDSsf & BAI)
- Also, the frequency of anxiety and depressive symptomatology.

**Methods:** Cross-sectional study in Ilion Municipality (Greece). A total of 501 participants were 60+ years old. **Results**: 6.8% of the participants have moderate to severe and 19.8% have mild to moderate depressive symptomatology. 5.9% have severe 15.4% moderate and 25.3% mild anxiety symptomatology. For the male participants, the x^2^ product reveals a statistically significant correlation (*p* < 0.05) for the variables age, family status, spouse, stressful events in adulthood, leisure time activities (LTA) and physical exercise. Living alone and the number of LTAs are correlated with anxiety symptomatology. In female participants fewer variables are correlated with depressive symptomatology in comparison to men but the variables correlated with anxiety symptomatology are more. **Conclusions**: Sociodemographic characteristics and stressful life events play crucial roles, sometimes in vulnerability, fostering and sometimes in resilience, preventing the manifestation of anxiety symptomatology and depressive symptomatology in the elders.

**Keywords:** depressive symptomatology; anxiety; elders; stressful life events

### 2.45. Football as a Possible Risk Factor for Alzheimer’s Disease

**Maria Styliani Chrysochoou, Anthoula Tsolaki, Elina Karathanasi, Anna Koutoupa, Konstantinos Lysitsas and Magda Tsolaki**

Alzheimer Hellas

**Abstract: Background**: The relationship between dementia and football has been the subject of increasing research attention in recent years. Studies have shown that repeated hits to the head are associated with early neuroinflammation, a factor that may lead to neurodegeneration, reduced white matter integrity and worse performance on cognitive tests. Dementia symptoms in football players may include memory loss, concentration difficulties, confusion and behavioural changes. In addition, anatomical and physiological changes, such as brain damage associated with chronic traumatic encephalopathy (CTE) have been observed. Furthermore, increased mortality due to neurodegenerative diseases has been reported in football players compared to the general population and the frequency of prescription of dementia related medication is higher in this population group. **Methods:** This study presents the results of a comparison between 14 patients—7 former football players with AD and 7 AD patients with no prior involvement in football—regarding the progression of dementia. **Results:** The data collected from the neuropsychological assessment, MRI and biomarker analysis identify the absence of statistically significant differences in cognitive function, functionality in daily activities and depression. **Conclusions:** Football and non-football AD patients attending dementia day care centres exhibit similar cognitive and psychological conditions, as well as an equivalent degree of neurodegeneration.

**Keywords:** football; dementia; Alzheimer’s disease; chronic traumatic encephalopathy

### 2.46. Discrepancies in Subjective Perceptions of Hydrocephalus Management and Self-Reported Outcomes

**Leonidas Trakolis ^1,4^, Julian Zipfel ^1,2,3^, Filip Zoltan ^2,3^, Cristina Kohlmann-Dell’Acqua ^1,2,3^ and Susan Noell ^1^**

^1^  Department of Neurotechnology and Neurosurgery, University Hospital Tuebingen, 72076 Tübingen, Germany
^2^  Centre for Clinical Studies, University Hospital Tuebingen, 72076 Tübingen, Germany
^3^  Neuropsychiatric Study Centre, University Hospital Tuebingen, 72076 Tübingen, Germany
^4^  Department of Neurosurgery, St. Luke Hospital, 552 36 Thessaloniki, Greece

**Abstract: Background**: The diagnosis of hydrocephalus has increased rapidly over the years, leading more patients to some type of treatment and improving their quality of life. However, many of them may have to live with some chronic disability and may require multiple surgeries. In fact, patients’ satisfaction with their quality of life may decrease over the years. **Methods:** In this retrospective study, our experienced team created self-report questionnaires and distributed them to all patients visiting the hydrocephalus outpatient clinics at the University Clinic in Tübingen, Germany, between 1 January 2020, and 31 March 2023. Patients completed these forms voluntarily, some of them more than once. **Conclusions:** Merely half of the patients with hydrocephalus were able to correctly indicate the treatment they had received. The type of shunt valve did not affect the rate of self-reported symptoms. The symptoms and subjective benefits did not differ in the different types of provided therapy. Poor patient knowledge could correlate with poor self-reported quality of life. Healthcare professionals should always be considerate of patients and use simple language without difficult terminology when speaking with them.

**Keywords:** hydrocephalus; quality of life; brain valve; therapy

### 2.47. Repeated Lumbar Drainage Puncture in Idiopathic Normal Pressure Hydrocephalus—Are There Patients Who Benefit?

**M. Tsolaki, A. Koutoupa, I. Tarnanas, Ch. Pierrakos and A. Tsolaki**

Alzheimer Hellas

**Rationale:** Cerebrospinal fluid (CSF) drainage can improve neurological symptoms and quality of life in patients with idiopathic Normal Pressure Hydrocephalus (iNPH). However, the patients’ responses vary. We hypothesize that clinical characteristics of the patients and response after the drainage lumbar puncture can predict the efficiency of the CSF drainage. Material and methods. We reviewed the follow up of 53 outpatients exhibiting cognitive impairment and incontinence or gait abnormalities and diagnosis of iNPH based on clinical and radiological criteria followed in a Memory Clinic who underwent at least two lumbar drainage punctures to alleviate symptoms. Lumbar punctures were repeated if the previous puncture improved patients’ symptoms. In case of failure of drainage puncture to improve symptoms, no further intervention was pursued in this regard. *Primary endpoint*: The primary endpoint was the duration of effective lumbar drainage puncture strategy over four years. *Secondary endpoints*: number of repeated punctures, intracranial pressure change. Analysis: Dementia type, age, presence of amyloid, tau, neurodegeneration, and improvement of symptoms following the initial lumbar puncture was evaluated as predictors of lumbar puncture efficiency. In the first step, Least Absolute Shrinkage and Selection Operator (LASSO) regression was employed to generate a predictive score, using as dependent variable the efficiency of the first puncture defined as either no need for a second puncture within one year or, if required, a second puncture which succeeded to alleviate patient’s symptoms. A ROC analysis identified the optimal cut-off value for the predictive score, dividing the population into two groups. Kaplan-Meier survival analysis was performed over a 4-year period to compare the duration of lumbar puncture efficiency between these groups. The relationship between the cutoff value and successful drainage puncture strategy at 4 years was further examined with the Cox proportional hazard analysis. Results: LASSO analysis identified AD or MCI with amyloid positive (A+)-according to the abnormal ratio Aβ1-42/Aβ1-40 in CSF- and age over 75 years as major negative predictive indicators. Conversely, Phospho-tau protein presence (T+), incontinence improvement, memory improvement and speech improvement following first lumbar drainage puncture were found to be positive predictive factors. The combination of these factors resulted in a dichotomy among patients, with those identified as high score undergoing a longer duration of effective drainage punctures than those with low score (1.5 [1.0–2.5] years vs. 0.6 [0.5–1.3] years; long-rank test *p* < 0.01) and exhibiting a reduced risk of drainage puncture failure over the subsequent 4 years (HR 0.38, 95%CI: 0.21–0.73; *p* < 0.01). A clinically relevant decision tree was feasible based on these binary factors. Conclusions: The combination of clinical factors before the first drainage punctures and the subsequent improvement of symptoms can be used as predictors for the potential benefits of drainage in patients with iNPH. Future studies should evaluate this approach for patients with iNPH and future decision and selection for shunt placement.

**Keywords:** normal pressure hydrocephalus; repeated lumbar drainage puncture; prediction of improvement

### 2.48. Assessing the Linguistic Deficits Combined with MRI Brain Volumetry Across the Alzheimer’s Disease Spectrum

**Maria Kaltsa ^1,2^, Anthoula Tsolaki ^1,2,3^, Ioulietta Lazarou ^4^, Ilias Mittas ^1^, Mairi Papageorgiou ^1^, Despina Papadopoulou ^1^ and Magda Tsolaki ^1,2,3^**

^1^  Aristotle University of Thessaloniki, Greece
^2^  Laboratory of Neurodegenerative Diseases, Center for Interdisciplinary Research and Innovation (CIRI-AUTH), Balkan Center, Aristotle University of Thessaloniki
^3^  Greek Alzheimer’s Association and Related Disorders (GAARD)/Alzheimer Hellas, Greece
^4^  Information Technologies Institute, Centre for Research and Technology, Hellas

**Abstract: Background**: The assessment of language deficits can be valuable in the early clinical diagnosis of neurodegenerative disorders, including Alzheimer’s Disease (AD). In the present study we are assessing the contribution of volumetric analysis of Brain Magnetic Resonance Imaging (MRI) along with lexical and grammatical indices in the early diagnosis of dementia due to AD. **Methods:** We have collected data from cognitively intact elder speakers (HC) and speakers with Subjective Cognitive Impairment (SCI), Mild Cognitive Impairment (MCI) and AD at the early stages (N: 109). A 3D structural MRI was acquired from each participant and the following regions of interest were examined: total brain volume, hemisphere volume, hippocampus, amygdala, thalamus, cerebellum, superior temporal gyrus, inferior frontal gyrus, the posterior cingulate gyrus and the precuneus. **Results:** The volumes were measured bilaterally and atlas-based volumetry was implemented to assess brain volume. In reference to the assessment of language skills, we employed spoken language data and indices tapping on lexicon and syntax. The analysis revealed the use of pronouns and the frequency of internal state terms discriminate MCI and AD groups from HC and SCI ones, while syntactic complexity reflected in the frequency of subordinate clauses and language errors discriminate all groups (*p* < 0.05). Correlation analysis of the linguistic variables and brain structure volumes revealed a close relationship between the two types of biomarkers with hippocampus, amygdala, thalamus, cerebellum areas in both right and left hemispheres correlating to lexical and syntactic markers (*p* < 0.001 apart from cerebellum areas ~ lexical complexity index *p* < 0.05). **Conclusions:** Language assessment was shown to be a novel promising tool, a sensitive biomarker that can detect early signs of degeneration due to AD dementia.

**Keywords:** language assessment; MRI; Alzheimer’s disease (AD); Subjective Cognitive Impairment (SCI); Mild Cognitive Impairment (MCI); healthy aging; Greek

**Funding:** Project DemLENS ‘Linguistic Perspectives on Dementia: an investigation of lexical, syntactic and content complexity in the narratives of Greek speaking patients with Mild Cognitive Impairment and Alzheimer’s Disease’ https://demlens.enl.auth.gr/ (accessed on 10 April 2025) is supported by the Hellenic Foundation for Research and Innovation (H.F.R.I.) under the “2nd Call for H.F.R.I. Research Projects to support Post-Doctoral Researchers” (Award No 163 | Project No 98109 | PI: Maria Kaltsa.

### 2.49. Management of Mild Cognitive Impairment

**Magda Tsolaki**

^1^  Neurologist/Psychiatrist & Theologist, GAARD, Palliative Care Unit “Panagia Glykofilousa” Thessaloniki, Greece
^2^  1st Department of Neurology, Aristotle University of Thessaloniki
^3^  Alzheimer Hellas, Thessaloniki, Makedonia, Greece
^4^  Laboratory of Neurodegenerative Diseases, Center for Interdisciplinary Research and Innovation (CIRI-AUTh), Balkan Center, Aristotle University of Thessaloniki, Thessaloniki, Makedonia, Greece

**Abstract:** The diagnosis of Mild Cognitive Impairment (MCI) due to Alzheimer’s Disease (AD) has many barriers: The most common barriers are patient-related, physician-related and resource-related barriers. If we succeed in overcoming all these barriers, we can design the care of the patients and the caregivers using 1. Non-pharmacological interventions, 2. Interventions for better quality of life, 3. Social support for caregivers, 4. Delay of institutionalization, 5. Recruitment in clinical studies with new medications or other interventions. In Greece we follow the international guidelines for the diagnosis of MCI due to AD doing neuropsychological, neurological and laboratory examination. We do not use yet amyloid or Tau PET, but we have the opportunity to do analysis of CSF proteins at a low cost. After the diagnosis we use natural products such as Crocus Sativus (125 mg/day), Extra Virgin Olive Oil (three spoons/per day), Oil from the seed of Pomegranate (5 drops/day), mountain tea, CBD (10 drops/day if there are behavioral problems) and homotaurine (VIVIMIND 50MG 1X1) if the patient is carrier of at least one APOE ε4. Also, we use Aniracetam 1500 mg/day, because we published a paper some years before and we found that it can delay the progression of cognitive symptoms. Recent study suggests that there is evidence that Aniracetam prevents the accumulation of Amyloid-β plaques in AD increasing BDNF, and modifying glutamate receptors.

**Keywords:** Mild Cognitive Impairment; diagnosis and management; natural products; pharmaceutical and non-pharmaceutical interventions

### 2.50. New Prospects in the Inhibition of Monoamine Oxidase-B (MAO-B) Utilizing Propargylamine Derivatives for the Treatment of Alzheimer’s Disease

**Filippos Panteleimon Chatzipieris ^1,2^, Georgios C. Vougioukalakis ^1,2^ and Thomas Mavromoustakos ^1,2^**

^1^  National and Kapodistrian University of Athens, 15771 Athens, Greece
^2^  Laboratory of Organic Chemistry, Department of Chemistry

**Abstract: Background:** Monoamine oxidase (MAO)-B plays a pivotal role in neurodegeneration. Specifically, the MAO enzyme catalyzes the oxidative deamination of a variety of monoamines. This reaction leads to the formation of aldehydes, together with H_2_O_2_ and ammonia. Hydrogen peroxide can generate additional reactive oxygen species (ROS), this way leading to neurotoxicity. When MAO is activated, it induces the amyloid-beta (Aβ) deposition via abnormal cleavage of the amyloid precursor protein (APP) and contributes to the generation of neurofibrillary tangles and cognitive impairment due to neuronal loss. **Methods:** Computational studies, docking, molecular dynamics, in silico, toxicity, pharmacokinetics and pharmacodynamics were conducted for propargylamine molecules bearing the triple bond in a non-terminal region of the structure. **Results:** The inhibition of this enzyme can manifest a positive impact in Alzheimer’s and Parkinson’s diseases. The main hMAO-B inhibitors encompass a terminal triple bond in their structure (propargylamine scaffold), which provides their potency. Recently, a new class of inhibitors has emerged, bearing the carbon-carbon triple bond not at the end of the chain. These molecules possess significant inhibitory activity against hMAO-B both in vitro and in silico. **Conclusions:** In our research we shall perform computational studies between these molecules and the hMAO-B enzyme and design hybrid compounds, which can inhibit both hMAO-B and acetylcholinesterase (AchE) or beta-secretase 1 (BACE1), thus tackling Alzheimer’s disease more effectively.

**Keywords:** monoamine oxidase (MAO)-B; Alzheimer’s disease; propargylamine derivatives; multi-target directed ligands (MTDLs)

### 2.51. Stress and Diagnosis of Dementia

**Maria Skotida**

Psychologist & Psychotherapist, MSc Student in Stress and Health Promotion, National and Kapodistrian University of Athens, Department of Medicine, MSc in Neurosciences and Neurodegenerative Diseases, Aristotelian University of Thessaloniki, MSc in Epidemiology and Health Promotion, University of West Attica

**Abstract: Background:** Chronic stress has emerged as a significant factor affecting neurological health. Persistent cortisol hypersecretion is directly associated with hippocampal shrinkage and a decline in cognitive function. This relationship raises concern due to its link with the onset and progression of dementia. Early diagnosis, effective stress management, and the adoption of preventive strategies are essential to reduce this risk. **Methods:** A selective review of the literature was conducted focusing on: The neurobiological impact of chronic stress on the brain. The association between elevated cortisol levels and cognitive decline. Interventions such as physical exercise, meditation, and social support. **Results:** The findings indicate that prolonged exposure to stress damages the hippocampus, leading to impaired memory and an increased risk of developing dementia. Additionally, stress management strategies—particularly physical exercise and psychosocial support—are shown to reduce cortisol levels and enhance cognitive function. **Conclusions:** Effective management of chronic stress is crucial for preventing dementia. Incorporating a combination of strategies, such as maintaining a healthy diet, engaging in regular physical activity, and fostering strong social networks, is a key tool in preserving neurological health. These interventions may lower the incidence of dementia and benefit public health overall.

**Keywords:** chronic stress; dementia; hippocampal damage; cognitive decline; stress management

### 2.52. The Diagnostic and Therapeutic Approach to Patients Within the Framework of the Operation of the Dementia Counseling Center

**Xenophon Fitsioris**

Doctor-Neurologist, Dementia Counseling Center Municipality of Kalamaria

**Abstract:** Alzheimer’s disease is spreading at epidemic rates worldwide. Although there is currently no etiological treatment, early diagnosis and symptomatic treatment are of great importance, as it is known that the disease begins approximately 25 years before the initial manifestation of symptoms. With the existing data, there is a possibility of slowing down the progression of the disease, treating the symptoms and improving the quality of life of patients and caregivers. The existence of structures such as Dementia Counseling Centers is of great importance for informing citizens and for the better and more effective treatment of the disease, which is not only a medical but also a social problem. At the Dementia Counseling Center of the Municipality of Kalamaria, over 600 people have been examined so far, a number that reflects the community’s interest in this serious disease. The center’s staff evaluates and examines patients, administers diagnostic tests and provides appropriate therapeutic treatment. We believe that the more people know, are informed and come into contact with similar structures, the more the expectation will increase for a better and more effective treatment of this serious medical and social problem for the benefit of patients and society in general.

**Keywords:** diagnostic; therapeutic approach; patients

### 2.53. Ethical Dilemmas in the Care of Patients with Dementia

**Dimitrios Theofanidis**

Department of Nursing, International Hellenic University, Greece.

**Abstract:** The increasing number of people with dementia will intensify the ethical issues that arise in clinical practice and research, in matters related to genetic testing and drug use. The ethical issues that health care professionals face in managing patients vary according to the stage of and progress of the disease. During the initial stages of the disease, the issue of pre-treatment planning is critical, particularly identifying a surrogate decision-maker and informing patients and carers about the challenges of care and treatment options, as the cognitive functions are expected to diminish in the later stages of the disease. In the intermediate stage of the condition, issues of caregiver stress and progressive loss of independence arise as well. In the final stage, the most important dilemmas are concerned the organization of home care, including the implementation of palliative care plans.

**Keywords:** ethical dilemmas; patients; dementia

### 2.54. Social Interventions and Personalized Patient Care

**Eleni Kakali**

Social Worker, Dementia Counseling Center, Municipality of Kalamaria.

**Abstract:** The social worker has undertaken the task of supporting citizens who face memory difficulties. This process includes multiple stages, aiming at good assessment and specialized care for each beneficiary. Initially, interested parties complete a consent form, which ensures the protection of their personal data. The social worker conducts a detailed interview to record the individual’s social history, aiming to obtain a comprehensive picture of their situation and needs. Her intervention also includes information on benefit policies and programs that may benefit. Depending on their situation and needs, the social worker may suggest referrals to other structures and services, during enhance their psychological and cognitive well-being. The registration process includes the assessment of the socio-economic factors that influence the beneficiary’s life. Understanding these aspects is crucial and aims both to meet their needs and to promote a healthy balance between abilities and goals. She aims to improve the quality of life of the individuals, through holistic and targeted interventions that promote their physical, social and mental health. This process is not only supportive but also empowering, promoting their autonomy.

**Keywords:** social; interventions; social worker

### 2.55. The Role of the Nurse and His Contribution to the Proper Treatment of Patients and Caregivers

**Paraskevi Papageorgiou**

Nurse at the Dementia Counseling Center of the Municipality of Kalamaria.

**Abstract:** With respect for the patient’s personality, maintaining their privacy, the first contact is made with the nurse and the family environment. During the medical history taking, the nurse, through observation, records the onset and course of the symptoms. A detailed record is also made of concomitant diseases, laboratory tests, medication received by the patient, as well as the views of the family environment. The nurse archives and updates the records after each neurological assessment. Provides clear instructions for proper medication administration. Counseling on nursing care issues in advanced stages of the disease. Also informing relatives regarding the patient’s ability to live independently and their ability to drive. The nurse has the ability, through technology, to contribute to the interconnection, sending of electronic records to secondary, tertiary healthcare. Implements, in collaboration with the other members of the structure, mental empowerment programs (mental and physical LLM-CARE). Creates personalized care plans. With the aim of promoting health, participates in speeches and prevention and community information actions.

**Keywords:** in consciousness; communication; teamwork; devotion

### 2.56. Legal Problems and Their Treatment

**Sotirios Fitsioris** **, Lawyer**

**Abstract:** The ethical and legal dilemmas that arise in a dementia consultation are numerous and complex. One of the main issues concerns the patients’ capacity to consent. Often, patients with dementia may not be able to understand the consequences of their actions, which poses challenges in providing information and ensuring the transaction. The mental deficits experienced by a person with dementia may lead to legal support for providing additional assistance, or deprivation of their legal rights and obligations. In addition, there is a need to protect the privacy and personal data of patients, and healthcare professionals must ensure that this information is not used inappropriately. Another issue is family dynamics, as relatives are often involved in decision-making, causing conflicts of interest. Finally, access to services and allocation are important issues, as patients’ needs may exceed available services. These dilemmas require good communication and collaboration between health professionals, patients, and families.

**Keywords:** legal and ethical issues; patients

### 2.57. The Role of Physiotherapy in the Prevention and Cure of Patients with Alzheimer

**Alexandra Hristara-Papadopoulou, Ourania Papadopoulou and Anna Halkia**

Department of Physiotherapy, School of Health Sciences, International Hellenic University, Thessaloniki, Sindos

**Abstract:** Alzheimer’s disease is a disease that has taken on great proportions in our days. It is one of the greatest challenges of the 21st century worldwide. Symptoms begin slowly, with the most prominent being memory loss, and in the early stages can usually be interpreted as signs attributable to normal aging. As the course progresses, more and more symptoms of a cognitive, neuropsychological, executive, and functional nature appear. Pharmaceutical and non-pharmacological interventions partially control symptoms and slow the progression of the disease. Early physiotherapy intervention and appropriate treatment program planning are increasingly necessary, because they aim to maintain the patient’s independence for as long as possible, improve quality of life, and slow the course of the disease. Constant mental alertness and physical exercise are recommended as a method of prevention. An important part of physiotherapy intervention is the assessment to assess the patient’s condition, stage of the disease, posture, balance and gait, which conditions depend primarily on muscle strength and synergy. The physiotherapy program includes relaxation techniques, therapeutic exercises to activate circulation and metabolism and strengthen the muscular system, breathing exercises combined with active movements of the upper limbs, unilateral and bilateral with movements of the arms and torso), teaching and application of diaphragmatic breathing, as well as therapeutic massage techniques (classical-lymphatic massage-reflexology). The therapeutic program can be combined with other forms of therapy such as memory therapy, dance therapy, music therapy, play therapy, mirror therapy, self-therapy and the technique of auricular neuromodulation. **Methods**: For the literature search, a search was conducted in PUBmed, PEDro, HEAL-Link exclusively in English, with the following keywords: Alzheimer’s disease, Physiotherapy, Prevention, “therapeutic exercise”, Alternative forms of treatment. The following review included clinical trials. Of the 63 studies found, only 11 clinical criteria: 2000–2025, Alzheimer’s patients aged >50 years and underwent physiotherapy intervention. **Results**: The role of physiotherapy proved to be very important in both the prevention and management of the disease. The benefits concerned the patient’s physical and psychological condition, motor and cognitive function. The physiotherapy intervention strengthened the core muscles to improve posture and gait. The respiratory muscles helped improve respiratory function. There was also an increase in verbal ability, psychological mood, decreased anxiety, and improved daily activities. Finally, the absorption of nutrients into the blood improved, which over time led to an improvement in BMI. **Conclusions:** In conclusion, the role of physiotherapy is very important both in the management of the disease, as well as in the course and prevention of possible harmful complications. The benefits it offers concern the patient’s physical condition, as well as of course the psychological state.

**Keywords:** Alzheimer’s disease; physiotherapy; prevention; therapeutic exercise; alternative forms of treatment

### 2.58. The role of Cannabinoids in Dementia

**Magda Tsolaki**

^1^  Neurologist/Psychiatrist & Theologist, GAARD, Palliative Care Unit “Panagia Glykofilousa” Thessaloniki, Greece
^2^  1st Department of Neurology, Aristotle University of Thessaloniki
^3^  Alzheimer Hellas, Thessaloniki, Makedonia, Greece
^4^  Laboratory of Neurodegenerative Diseases, Center for Interdisciplinary Research and Innovation (CIRI-AUTh), Balkan Center, Aristotle University of Thessaloniki, Thessaloniki, Makedonia, Greece

**Abstract:** Neurodegenerative diseases and dementia are a global challenge for the aging population with the increasing incidence of the most common degenerative disease which is Alzheimer’s disease (AD). No drugs have yet been found that treat AD. The effects of *Cannabis sativa* L. on brain function have been known for thousands of years. >550 chemicals and >100 plant cannabinoids or phyto-cannabinoids have been isolated from Cannabis sativa, including D9-tetra-hydro-cannabinol (THC) and cannabidiol (CBD). Cannabidiol (CBD) and tetrahydro-cannabinol (THC) are the most studied cannabinoids and interact with endo-cannabinoid receptors in various human tissues. THC, D9-tetrahydro-cannabinol, is psychoactive, antiemetic, analgesic, causes muscle relaxation and increased appetite. CBD, Cannabidiol, is neuro-protective, analgesic, antipsychotic, and anti-epileptic. Their applications have a wide range. Their neuro-protective action is due to many mechanisms, the main ones being: 1. Their effect on cholinergic dysfunction, 2. Inhibition of beta amyloid accumulation and its toxicity and 3. Their antioxidant properties. Neuroimaging studies have shown that acute administration of CBD causes significant changes in brain activity and connectivity during resting and while performing mental activities in both healthy volunteers and psychiatric patients. We have experience of two clinical trials for short- and long-term treatment of behavioral disorders in patients with various neurodegenerative diseases. In both the 15-day short term study and the 6-month long term study the results were very positive in the behavioral disorders of patients with neurodegenerative diseases. Two years later the patients in the second study were examined and found that deaths in the control group of patients taking the current treatment for behavioral disorders were more than doubled compared to those taking CBD 3%. However, multi-center studies are needed to examine the effect of cannabinoids on both behavioral disorders and cognitive dysfunction.

### 2.59. Cerebral Amyloid Angiopathy and Application of Anti-Amyloid Antibodies in Alzheimer’s Disease

**Effrosyni Koutsouraki**

Aristotle University of Thessaloniki, 1st Department of Neurology, AHEPA University Hospital

**Abstract:** Alzheimer’s disease (AD) is characterized by intraparenchymal accumulation of β-amyloid (Aβ) and reduced perivascular clearance, resulting in increased Aβ in the vessel wall and smooth muscle fiber disorders. After initiation of anti-amyloid antibody (AAA) therapy, vessels with pre-existing amyloid angiopathy exhibit a local inflammatory reaction and become more susceptible to extravascular events, resulting in amyloid-related imaging abnormalities (ARIA). Individuals most affected by ARIA are those carrying apolipoprotein e-4 alleles. Although the approval of anti-amyloid antibodies is a significant progression in the treatment of AD, safety issues need to be appropriately evaluated. The high incidence of concurrent cerebral amyloid angiopathy (CAA) in the AD population leaves many patients vulnerable to ARIA-related morbidity. Careful evaluation for evidence of CAA will be crucial to limit the risks associated with immunotherapy. There is a great need for a better understanding of ARIAs and the interaction of cerebral amyloid angiopathy pathology and NA to improve existing therapies and design new therapies, perhaps combination therapies, to modify the disease more safely.

**Keywords:** cerebral amyloid angiopathy; anti-amyloid antibodies; ARIA; Alzheimer’s disease

### 2.60. Clinical and Imaging Diagnostic Criteria of Cerebral Amyloid Angiopathy

**Athanasia Athanasaki, Aikaterini Theodorou, Ioanna Tsantzali, Aikaterini Foska, Stella Fanouraki, George P. Paraskevas and Georgios Tsivgoulis**

Second Department of Neurology, “Attikon” University Hospital, School of Medicine, National and Kapodistrian University of Athens, Athens, Greece

**Abstract: Background**: Cerebral amyloid angiopathy (CAA) is suspected in patients over 50 years old who present with acute or chronic hemorrhagic manifestations and/or characteristic white matter lesions on brain MRI, in the absence of any other etiology. **Methods:** We performed a systematic review of the literature search of all original articles published around the topic of cerebral amyloid angiopathy criteria. **Results:** The establishment of the Boston criteria v.2, the revised version since 2010 (v.1.5), has contributed to increase the diagnostic accuracy, regarding especially probable CAA. This revision of the criteria included in the imaging criteria non-hemorrhagic lesions and more specifically the presence of either dilated perivascular spaces in centrum semiovale or oval white matter lesions with increased signal on the T2 sequence. The consistent clinical presentation encompasses patients with intracerebral hemorrhage, transient focal neurologic events or cognitive impairment. The identification of the above radiological findings as well as other reported like superficial cerebellar microbleeds and cortical microinfarcts, is significant and occasionally related to disease prognosis. **Conclusions:** It is considered necessary to perform a brain MRI, including the sequence of susceptibility weighted imaging (SWI), which demonstrates increased sensitivity in the detection of hemorrhagic manifestations, in suspected patients.

**Keywords:** revised Boston criteria v.2; white matter lesions; diagnostic accuracy

### 2.61. The Prevalence of CHIP in Patients with Dementia

**Vasiliki Gougoula ^1^, Georgia Kaiafa ^1^, Eleftheria Ztriva ^1^, Thomas Tegos ^2^, George Ntaios ^1^ and Christos Savopoulos ^1^**

^1^  1st Propaedeutic Department of Internal Medicine, University General Hospital of Thessaloniki, AHEPA
^2^  1st Neurology Department, University General Hospital of Thessaloniki, AHEPA

**Abstract: Background**: Clonal hematopoiesis of indeterminate potential (CHIP) is characterized by acquired mutations (VAF ≥ 2%) in hematopoietic stem cells without evidence of hematologic malignancy. It becomes more prevalent with aging, prior exposure to chemotherapy or radiation therapy, chronic inflammation, and environmental stressors. While CHIP is associated with an increased risk of hematologic cancers and cardiovascular disease, including a 19% higher risk of ischemic stroke—particularly hemorrhagic stroke—its potential impact on neurodegenerative disorders remains unclear. **Methods:** A literature search was conducted in PubMed and Scopus using the keywords “CHIP”, “dementia”, “cognitive decline”, and “neurodegeneration”. Articles with abstracts in English, published between January 2020 and January 2025, were included in the analysis. Duplicates and non-relevant records were excluded. **Results:** Seven relevant records were identified. These records suggested that CHIP may have a protective role against the development of Alzheimer’s disease, potentially influencing neuroinflammatory or immune-related pathways. However, the mechanisms underlying this association remain to be fully elucidated. **Conclusions:** The current literature linking CHIP to dementia is limited, and existing studies provide only preliminary evidence. Further research involving larger and more diverse populations is necessary to clarify the potential neuroprotective effects of CHIP and to explore its broader implications for age-related cognitive decline.

**Keywords:** CHIP; dementia; clonal hematopoiesis; genetic mutations

### 2.62. The Relationship Between Mild Behavioral Impairment and Genetic Predisposition Factors for Cognitive Impairment

**Efthalia Angelopoulou ^1^, Alexandros Hatzimanolis ^2^, Nikolaos Scarmeas ^1^ and Sokratis Papageorgiou ^1^**

^1^  1st Department of Neurology, Aiginition Hospital, National and Kapodistrian University of Athens
^2^  1st Department of Psychiatry, Aiginition Hospital, National and Kapodistrian University of Athens

**Abstract: Background**: Mild Behavioral Impairment (MBI) refers to the emergence of new, persistent neuropsychiatric symptoms (NPS) in older adults that are not attributable to a pre-existing psychiatric disorder and may serve as early indicators of cognitive decline. The aims of this study include the investigation of the prevalence, incidence, genetic background and underlying pathophysiology of MBI in Greece. **Methods:** This study is part of the population-based, prospective epidemiological study HELIAD, including approximately 2000 individuals aged 65 and older in Greece. The polygenic risk scores (PRSs) for amyloidopathy, tau proteinopathy, and vascular pathology will be analyzed and calculated for each participant. The association between each PRS (including and excluding Apolipoprotein E genotype) and MBI, each MBI domain (emotional dysregulation, decreased motivation, impulse dyscontrol disorders, social inappropriateness, and psychotic symptoms), and incident cognitive decline will be explored, using logistic regression models. **Results:** The results of this study are expected to aid in clarifying the prevalence, incidence and genetic basis and pathophysiology of MBI, at the earliest dementia stages. **Conclusions:** This study will provide valuable insights into the genetic susceptibility to early NPS in dementia, potentially contributing targeted screening, personalized prevention and early intervention strategies.

**Keywords:** mild behavioral impairment; dementia; Alzheimer’s disease; polygenic risk scores

### 2.63. The Results of Physiotherapy Intervention in End-Of-Life Dementia Patients

**Vasilis Stamos ^1^ and Magda Tsolaki ^2^**

Greek Association of Alzheimer’s Disease and Related Disorders (GAARD), Palliative Care Unit “Panagia Glykofilousa” Thessaloniki

^1^  Psychotherapist, GAARD, Palliative Care Unit “Panagia Glykofilousa” Thessaloniki, Nik. Plastira 65, 54250, Greece
^2^  Neurologist/Psychiatrist & Theologist, GAARD, Palliative Care Unit “Panagia Glykofilousa” Thessaloniki, Nik. Plastira 65, 54250, Greece

**Abstract: Background:** The Palliative Care Unit “Panagia Glykofilousa” hosts end of life dementia patients. Similar to other non-pharmacological interventions, Physiotherapy-Movement therapy brings positive effects, since it helps in disease management. This paper aims to point out how physiotherapy contributes to dementia treatment, through the utilization of current literature. **Methods:** Since the world literature lacks of a relevant scale, the scale MARIA was created. This scale evaluates patients upon admission to the palliative care unit and for the whole duration of their stay. This way, we remain informed of their motor condition. Furthermore, during their stay, we mobilize patients in groups (using their wheelchairs). They go to church or to the yard (weather permitted), watch a movie, listen to music, eat, meet their loved ones and many more. All these interventions aim for the quality of life and patients’ dignity to be maintained, as much as possible, given that they are in their late stages of life. **Results:** The results of the physiotherapy intervention were the following: In the six (6) months time frame there were 28 patients included. The data follows non-normal distribution, so the Wilcoxon signed-rank test was used. The overall score before the intervention was 20.28 and after the intervention was 21.10 (*p* < 0.5568). In the one (1) year time frame, 18 patients were included. The data follows normal distribution, so the *t*-test was used. The overall score before the intervention was 19.27 and after the intervention 20.05 (*p* < 0.3639). Therefore, although we noticed improvement of the patients who participated in this research, the difference before and after was not statistically significant. **Conclusions:** In conclusion, physiotherapy helps end-of-life dementia patients.

**Keywords:** physiotherapy-movement therapy; dementia; non-pharmacological interventions; scale MARIA; motor condition

### 2.64. The Effect of Pomegranate Seed Oil in Later Stage Dementia Patients

**Anna Koutoupa ^1^, Marianna Tsatali ^1^, Mahi Kozori ^1^, Vasiliki Lagouri ^2^ and Magda Tsolaki ^1,3,4^**

^1^  Greek Association of Alzheimer’s Disease and Related Disorders (Alzheimer Hellas), Plastira 65, P.C. 542 50, Thessaloniki, Greece
^2^  National Hellenic Research Foundation (N.H.R.F), 48 Vassileos Constantinou Avenue, P.C. 11635, Athens, Greece
^3^  School of Medicine, Aristotle University of Thessaloniki, University Campus, P.C. 54124, Thessaloniki, Greece
^4^  Center for Interdisciplinary Research and Innovation (CIRI), Aristotle University of Thessaloniki, Balkan Center, P.C. 57001, Thessaloniki, Greece

**Abstract: Background**: Pomegranate seed oil (PSO) contains high levels of fatty acids that have antioxidant and anti-inflammatory properties. The aim of this study was to evaluate the effect of PSO in later stage dementia, focusing on urinary tract infection, other infections, hospitalization and antipsychotic medication. **Methods:** Nine later stage dementia patients from the Alzheimer Hellas patient care unit were administered 5 drops of PSO on a daily basis for 6 months and another nine later stage dementia patients served as the control group. **Results:** According to Chi-Square analysis, PSO had a positive effect on urinary tract infections with only 22% of patients experienced reinfection, while in the control group the reinfection was 100% (*p* = 0.05). Reduction or discontinuation of antipsychotic medication was 43%, unlike the control group, where no changes occurred (*p* = 0.05). Hospitalization rate was lower (*p* = 0.052) and there was no significant difference in other infections. Although there were 3 deaths (33%) in the experimental group compared to 2 in the control group (*p* = 0.053), these were unrelated to PSO and due to the progression of the disease. **Conclusions:** PSO is a 100% natural product, that can be used safely in later stage dementia and could serve as an important part of holistic treatments for dementia.

**Keywords:** final stage dementia; pomegranate seed oil; infections; antipsychotics

### 2.65. Palliative Care Units for Patients with Moderate to End-Stage Dementia: A Modern Necessity and the Role of the Social Worker

**Aikaterini Boutikou ^1^, Christina Kadi ^2^ and Magdalini Tsolaki ^3^**

^1^  Social Worker, Greek Association of Alzheimer’s Disease and Related Disorders (GAARD), Palliative Care Unit “Panagia Glykofilousa” Thessaloniki, Greece
^2^  Social Worker, MSc, Company for the Development of Community Mental Health Services for Children and Adults “Panakeia”, Panagia Kyra, Rhodes, Greece
^3^  Neurologist/Psychiatrist & Theologist, Greek Association of Alzheimer’s Disease and Related Disorders (GAARD), Palliative Care Unit “Panagia Glykofilousa” Thessaloniki, Nik. Plastira 65, 54250, Greece

**Abstract: Background**: Dementia is becoming a global concern, representing a major public health issue. The progressive deterioration of the disease increases the level of dependency of patients on their caregivers. Especially those in terminal stages need continuous and intensive support. This responsibility often falls on their children, who are middle-aged and already burdened with numerous professional and family obligations. Given the significant psychological, physical, and financial strain on caregivers, the demand for patient accommodation in Palliative Care Units is rising. Specifically, in Thessaloniki we have had more than 480 requests from caregivers regarding patient admissions. Similarly, in Rhodes, there have been 100 requests, in Corfu 150, and in Megara, the number has reached 200. **Methods:** Literature review on PubMed and contact with colleagues in existing Palliative Care Units. **Results:** Social workers face the daily distress and challenges of caregivers, as well as their need for relief from continuous care responsibilities. Through their role- which includes assessing needs, preventing and addressing socio-economic challenges, securing insurance rights, and providing social and emotional support to families- they must highlight the urgent need for more state-run facilities. **Conclusions:** Expanding these services would significantly improve the quality of life for both patients and their caregivers.

**Keywords:** palliative care units; caregivers; care; social workers

### 2.66. The Importance of Maintaining a Healthy Body Weight in Elderly Patients with End-Stage Dementia: Effects on Nutrition and Survival

**Aimilia Vassilopoulou**

Clinical Nutritionist-Dietitian, Psychologist, Asst Prof Diet and Nutrition, International Hellenic University, Visiting Professor, University of Milan

**Abstract:** Maintaining a stable and healthy body weight in elderly individuals with end-stage dementia is a critical factor in improving their quality of life and survival. The nutritional status of these patients is closely linked to a reduced risk of health complications and protection against infections, which are common at this stage of the disease. This presentation focuses on the importance of adequate protein intake, as well as a diet rich in antioxidants and anti-inflammatory components. These dietary choices not only contribute to the preservation of muscle mass and physical function but also help reduce inflammatory processes that worsen the patients’ condition. The aim of this presentation is to highlight the relationship between nutritional status and survival, emphasizing the need for targeted nutritional interventions as part of the holistic care of elderly individuals with end-stage dementia.

### 2.67. The Role of Valinyl-Omotaurine in the Treatment of Cognitive Disorders: Recent Data

**Magda Tsolaki ^1,2,3^, Anna Koutoupa ^1,2,3^, Thanos Hadjikostopoulos ^1,2,3^, Anna Anastasiou ^2,3^, Eleni Tzekaki ^2,3^ and Anastasia Pantazaki ^2,3^**

^1^  Hellenic Alzheimer’s Disease Society
^2^  Laboratory of Neurodegenerative Diseases, KEDEC
^3^  Aristotle University of Thessaloniki

**Abstract:** In 2018 alone, 59 new drugs were approved by the FDA for various diseases, and the last symptomatic drug for AD in 2003 was MEMANTINE. However, many studies have been done with different goals without getting the results we wanted, because our goals have not been the ones that would lead to an effective treatment. On 6 January 2023, the FDA (US Food and Drug Administration) approved on 6 January 2023 and on 14 November 2024, Lecanemab, an intravenous monoclonal antibody targeting Amyloid β oligomers and protofibrils. It was approved because it met all primary and secondary targets: It reduced clinical worsening with CDR-SB by 27%, with ADAS-cog14 by 26%, with ADCOMS by 24%, and with ADCS-MCI-ADL by 37%. However, it has serious adverse effects, such as reactions where the intravenous administration is done in 26.4%, cerebral edema in 12.6% and cerebral hemorrhage in 17.3%. On the other hand, we have in our quiver, currently in the form of a dietary supplement, a preparation of seaweed, which is also linked to Aβ, is a pill, and has no serious side effects. We can use it directly at the moment in patients with APOE ε4, based on a study done in the past, and also a recent study, so we can delay amyloidosis and wait for the study to be approved by the FDA and EMA drug agencies, which was fast-tracked by the NIA, so that in the near future we can prescribe it without patient involvement. Currently we have results from a recent study showing 21% less RAVLT impairment than seen in the placebo and 25% less hippocampal atrophy than seen in the placebo group of ADNI. **Conclusions:** The study involves 1. patients with MCI and mild AD (MMSE > 22 and CDR 0.5–1) with positive biomarkers in CSF and carriers of AROE ε4. 2. Patients took ALZ-801 265 mg orally, twice daily for two weeks and then 1 time daily and found significant reduction in p-tau181, a marker of neurodegeneration from week 13s and persisted until week 104s. 3. Comparing hippocampal atrophy with an external control group showed a reduction in the rate of atrophy and memory tests showed stability and a statistically significant correlation with hippocampal atrophy scores. 4. No serious adverse events and angiogenic edema were observed, supporting the promising benefit/risk profile of ALZ-801 in carriers of APOE ε4, which comprise 2/3 of AD patients. Finally, a small study of our own found that it stabilizes the clinical picture of patients and statistically significantly reduces plasma phospo-tau 181 in the above patients (*p* < 0.05), which is confirmed by the above international study^1^

**Keywords:** valilttamiprosate; lecanemab; phospo-tau

### 2.68. The Effect of Cognitive Impairment Stage on Driving Cessation in Greek Patients: A Long-Term Follow-Up Study

**Ioanna Giannoula Katsouri ^1,2^ and Magda Tsolaki ^2,3,4^**

^1^  Department of Occupational Therapy, University of West Attica, 12241 Athens, Greece
^2^  Medical School, Faculty of Health Sciences, Aristotle University of Thessaloniki, 54124 Thessaloniki, Greece
^3^  Laboratory of Neurodegenerative Diseases, Center for Interdisciplinary Research and Innovation (CIRI-AUTH), Balkan Center, Aristotle University of Thessaloniki, 10th km Thessaloniki-Thermi, 54124 Thessaloniki, Greece
^4^  Department of Caregiver’s Support, Greek Association of Alzheimer’s Disease and Related Disorders (GAADRD), 54643 Thessaloniki, Greece

**Abstract: Background:** Determining the appropriate time for driving cessation in older adults with cognitive impairment is a complex, multifactorial process that significantly impacts their quality of life and autonomy. However, the decision to cease driving is often delayed, increasing the risk of traffic accidents. **Methods:** This review aimed to map the existing international literature on factors contributing to delayed driving cessation. A literature search was conducted in PubMed and ScienceDirect for articles published in the last decade, using keywords such as “driving cessation”, “dementia”, and “elderly”. **Results:** The findings highlighted key factors contributing to the delay in driving cessation among older adults with cognitive impairment. These include the subjective perception of driving ability, the lack of standardized assessment tools by healthcare professionals, reluctance from family members to intervene, and the absence of alternative transportation options, especially in remote areas. **Conclusions:** The study underscores the need for systematic clinical assessments and enhanced support for older adults with cognitive impairment to prevent delays in driving cessation and reduce associated risks.

**Keywords:** driving cessation; elderly; cognitive impairment

**Funding:** University of West Attica,12241 Athens, Greece.

### 2.69. Cultivating a Forgiving Attitude in Counseling Older Adults

**Dimitra Vasileiou ^1^, Christos Pezirkianidis ^2^ and Despina Moraitou ^1^**

^1^  Laboratory of Psychology, Department of Cognition, Brain and Behavior, School of Psychology, Aristotle University of Thessaloniki (AUTh), 54124 Thessaloniki, Greece
^2^  Laboratory of Positive Psychology, Panteion University of Social & Political Sciences, 17671 Athens, Greece

**Abstract: Background:** In the effort to integrate positive psychology constructs into the counseling process, these constructs are applied to various cases and age groups. One such group is older adults. The goal of positive psychology interventions (PPIs) is to enhance well-being by empowering people’s character strengths (CS). This presentation examines the effectiveness of a PPI focused on forgiveness, applied to an 81-year-old-woman with no cognitive decline, residing in a nursing home in Thessaloniki. The aim was to cultivate a forgiving attitude toward a relative who died, with the goal of increasing her well-being and life satisfaction. **Methods:** The intervention was based on the Worthington REACH model, which focuses on the process of forgiveness through 5 steps: (1) expressing negative emotions, (2) cultivating empathy for the “offender”, (3) recognizing forgiveness of oneself by others, (4) committing to forgiveness, and (5) maintaining forgiveness as an element of self. The process was completed over four sessions with the institution’s psychologist. **Results:** The results showed that forgiveness was achieved, leading to a transformation of the older woman’s negative feelings and thoughts and an enhancement of her well-being. **Conclusions:** The intervention demonstrated the potential of forgiveness to improve well-being and life-satisfaction in older adults.

**Keywords:** positive psychology; forgiveness; character strengths; counselling

### 2.70. Resilience in Mild Cognitive Impairment (MCI): Examining the Level and the Associations of Resilience with Subjective Wellbeing and Negative Affect in Older Adults

**Styliani Olympia Tsormpatzoudi ^1^, Despina Moraitou ^2^, Vasileios Papaliagkas ^3^, Christos Perzikianidis ^4^ and Magda Tsolaki ^5^**

^1^  Psychologist, MSc, Laboratory of Medical Physics and Digital Innovation, Metropolitan College of Thessaloniki
^2^  Professor of Cognitive Geriatric Psychology, Department of Psychology, Aristotle University of Thessaloniki, founding member of the Laboratory of Neurodegenerative Diseases at the Center for Interdisciplinary Research and Innovation of the Aristotle University of Thessaloniki
^3^  Neurologist MD, PhD, MSc, Associate Professor, Department of Biomedical Sciences, School of Health Sciences of the International Hellenic University, Head of the Memory and Dementia Clinic of Euromedica Kyanous Stavros
^4^  Assistant Professor, Positive Psychology, Department of Psychology, Panteion University of Social and Political Sciences
^5^  Emeritus Professor of Neurology, Aristotle University of Thessaloniki, President of the Hellenic Alzheimer’s Disease and Related Disorders Federation, Coordinator of the Neurodegenerative Diseases Laboratory (KEDEK), Founder and Professor of the Postgraduate Studies Program “Neurosciences and Neurodegenerative Diseases”, Founder and Scientific Director of the 1st Terminal Dementia Boarding School “Panagia Glykofilousa”

**Abstract: Background**: This study examines the relationship between the cognitive state of participants [healthy-early mild cognitive impairment (MCI)–late MCI], subjective wellbeing (positive emotions, engagement, positive relationships, meaning in life, accomplishment, and negative emotions), negative psychological outcomes (depression, anxiety, stress), and psychological resilience. We expected that people with advanced MCI would perceive increased negative psychological outcomes, poorer psychological resilience, and lower levels of subjective wellbeing in contrast to early MCI and healthy participants. **Methods:** The study involved 30 healthy, 31 early, and 28 late MCI individuals. A series of questionnaires have been applied to assess these constructs. To examine the hypotheses of the study, path analysis (EQS program) was applied. **Results:** Results showed that early MCI individuals maintain the same levels of positive emotions and feelings of accomplishment with healthy peers. Late-stage patients present those feelings in a diminished form, which adversely impacts psychological resilience. Individuals with early and late MCI exhibit negative emotions and stress that impact their resilience; however, those with early MCI experience greater stress, negative emotions, depression, and anxiety. **Conclusions:** These findings may be utilized to design psychological interventions for resilience enhancement and support brain health in elderly adults who are at risk of neurodegeneration.

**Keywords:** mild cognitive impairment; subjective wellbeing factors; negative psychological symptoms; psychological resilience

### 2.71. Application of a Combination of Positive Psychology Interventions in Individuals with Early-Stage Alzheimer’s Disease to Strengthen Character Strengths: Examining Their Impact on Cognitive Functions and Brain Functioning

**Dimitra Vasileiou ^1^, Despina Moraitou ^1^ and Christos Pezirkianidis ^2^**

^1^  Laboratory of Psychology, Division of Cognitive and Experimental Psychology, Department of Psychology, Aristotle University of Thessaloniki
^2^  Laboratory of Positive Psychology, Panteion University of Social and Political Science

**Abstract: Background:** As there is no cure for dementia, the goal is to delay its progression. Non-pharmacological interventions appear to be more effective, safer, and more cost-effective than pharmacological ones, especially in the early stages. The purpose of this study is to investigate whether a combination of Positive Psychology interventions (PPIs) can enhance cognitive and brain functioning in individuals with Subjective Cognitive Decline (SCD), amnestic Mild Cognitive Impairment (aMCI), and mild Alzheimer’s disease (AD), by strengthening their Character Strengths (CS). **Methods:** Participants will be recruited from the Day Centers of Alzheimer Hellas and divided into three experimental and three control groups based on their diagnosis (SCD, aMCI, and mild AD) in a randomized manner. The experimental groups will undergo a 24-week PPI, while the control groups in cognitive treatment as usual. All participants will be evaluated in 4 phases: baseline, mid-intervention, post-intervention, and 9 months later, assessing cognitive functions, CS, well-being, and brain functioning. **Results:** It is expected that experimental groups will show higher cognitive and brain functioning compared to the control groups. SCD and aMCI participants are anticipated to maintain benefits 9 months later, with SCD participants benefiting the most, followed by aMCI and mild AD groups. **Conclusions:** The ultimate goal is to develop a simple and cost-effective intervention for dementia prevention.

**Keywords:** positive psychology; character strengths; non-pharmacological interventions for dementia; Alzheimer’s disease

### 2.72. Cognitive Decline Prediction Using Neuropsychological Assessment Results and Machine Learning Techniques

**Efstathia Tsarouchi ^1^, Emmanouil Tsardoulias ^1^, Eleni Poptsi ^2,3,4^, Madga Tsolaki ^3,4^ and Andreas Symeonidis ^1^**

^1^  Department of Electrical and Computer Engineering, Engineering School, Aristotle University of Thessaloniki, Thessaloniki, Greece
^2^  Laboratory of Psychology, Department of Experimental and Cognitive Psychology, School of Philosophy, Aristotle University of Thessaloniki, Thessaloniki, Greece
^3^  Laboratory of Neurodegenerative Diseases (LND), Center of Interdisciplinary Research and Innovation (CIRI-AUTH), Thessaloniki, Greece
^4^  Greek Association of Alzheimer’s Disease and Related Disorders (GAADRD/Alzheimer Hellas), Thessaloniki, Greece

**Abstract: Background**: Neurodegenerative diseases are one of the most crucial global health issues that significantly challenge patients, their families, and society. Early prediction is one of the most important tools for tackling the progress of neurodegenerative diseases, as it allows the creation of a concrete therapeutic plan. The objective of this study is the early prediction of an individual’s cognitive decline based on the timeline of their neuropsychological assessments (NPA). **Methods:** An analysis and processing of a dataset of multiple NPAs was conducted. The dataset was provided by Alzheimer Hellas and was grouped into two categories: (a) “No conversion” (n = 3740): no recorded decline between two consequent NPAs of the same individual, and (b) “Conversion” (n = 294). After investigating various sampling techniques, hyperparameter selection, and dimensionality reduction methods, multiple ML algorithms were utilized to successfully identify the pairs of consecutive NPAs in which a cognitive decline had occurred. **Results:** The best accuracy rate (84%) was achieved for the SVM model utilizing the “GroupKFold” validation technique. **Conclusions:** NPAs such as executive functions and episodic memory tests, as well as daily function and emotional disorders, have been “marked” as the most significant predictors of cognitive decline.

**Keywords:** machine learning; neuropsychological assessment; conversion prediction; cognitive decline

### 2.73. Cognitive Decline Category Recognition Through Text Features from Speech Transcription

**Georgios Kalomitsinis ^1^, Emmanouil Tsardoulias ^1^, Dimitris Nastos ^1^, Nikolaos Malamas ^1^, Eleni Poptsi ^2,3,4^, Madga Tsolaki ^3,4^ and Andreas Symeonidis ^1^**

^1^  Department of Electrical and Computer Engineering, Engineering School, Aristotle University of Thessaloniki, Thessaloniki, Greece
^2^  Laboratory of Psychology, Department of Experimental and Cognitive Psychology, School of Philosophy, Aristotle University of Thessaloniki, Thessaloniki, Greece
^3^  Laboratory of Neurodegenerative Diseases (LND), Center of Interdisciplinary Research and Innovation (CIRI-AUTH), Thessaloniki, Greece
^4^  Greek Association of Alzheimer’s Disease and Related Disorders (GAADRD/Alzheimer Hellas), Thessaloniki, Greece

**Abstract: Background**: Detecting the phases of cognitive decline is a challenge for the early diagnosis and management of neurodegenerative diseases. This paper aims to categorize the cognitive decline phases through text features derived from speech transcripts using machine learning. **Methods:** Textual transcript data of interviews of 84 subjects we used: Healthy Older Adults (HoA), n = 18; Subjective Cognitive Impairment (SCI), n = 19; Early Phase Mild Cognitive Impairment (EMCI), n = 33; Late Phase Mild Cognitive Impairment (EMCI), n = 14. The data were classified into two and four classes based on patient category, per person, and per question. Transcripts were further processed to extract additional linguistic features, seven machine learning algorithms were applied (Naive Bayes, Logistic Regression, KNN, SVM, Decision Tree, Random Forest, XGBoost), and three text processing models (FT, TFIDF, W2V) were evaluated. The results were validated using StratifiedKFold, LOSO, and LOGO. **Results:** Optimal results were achieved for two classes (healthy versus pathological aging) via Naive Bayes and Random Forest algorithms, W2V, and StratifiedKFold validation, with an average accuracy of 80.99% ± 3.29%. **Conclusions:** Categorization using machine learning and text processing can be a useful, non-intrusive tool for the early detection of cognitive decline.

**Keywords:** machine learning; speech transcription; natural language processing; early detection

### 2.74. Cognitive Decline Category Recognition Using R4Alz Battery and Machine Learning Techniques

**Olympia Giagkouli ^1^, Eleni Poptsi ^2,3,4^, Emmanouil Tsardoulias ^1^, Madga Tsolaki ^3,4^ and Andreas Symeonidis ^1^**

^1^  Department of Electrical and Computer Engineering, Engineering School, Aristotle University of Thessaloniki, Thessaloniki, Greece
^2^  Laboratory of Psychology, Department of Experimental and Cognitive Psychology, School of Philosophy, Aristotle University of Thessaloniki, Thessaloniki, Greece
^3^  Laboratory of Neurodegenerative Diseases (LND), Center of Interdisciplinary Research and Innovation (CIRI-AUTH), Thessaloniki, Greece
^4^  Greek Association of Alzheimer’s Disease and Related Disorders (GAADRD/Alzheimer Hellas), Thessaloniki, Greece

**Abstract: Background**: R4Alz is an innovative tool for the early diagnosis of subtle cognitive changes at the pre-clinical level, in the phase of Subjective Cognitive Impairment (SCI), aiming at early intervention and progress delay of neurodegenerative disorders. The goal is to create machine learning models that will be able to accurately and reliably predict the cognitive phase of the participants. **Methods:** Data from 175 participants were used, categorised into five groups: (1) cognitively healthy young adults (HyA) (n = 42, 21–39 years), (2) cognitively healthy middle-aged adults (HmaA) (n = 33, 42–57 years), (3) cognitively healthy older adults (HoA) (n = 14, 60–76 years), (4) people with SCI (n = 34, 54–86 years), and (5) people with Mild Cognitive Impairment (MCI) (n = 52, 58–84 years). Healthy adults were recategorized into a new group named “Healthy” (HyA, HmaA, and HoA). Different pre-processing techniques and machine learning algorithms were implemented and tested. **Results:** 83% accuracy in categorization between Healthy, SCI, and MCI, 94% between Healthy-SCI, 72% between SCI-MCI, 98% between Healthy-MCI. **Conclusions:** R4Alz can be used in the early identification of subtle cognitive changes before the onset of clinical symptoms, with significant accuracy in categorization between the Healthy and all other groups.

**Keywords:** machine learning; neurodegeneration; diagnostic tool; R4Alz

### 2.75. Implementation of a System to Assess the Suitability of Interiors for People with Dementia

**Dimitrios Triantafillidis ^1^, Emmanouil Tsardoulias ^1^, Eleni Poptsi ^2,3,4^, Madga Tsolaki ^3,4^ and Andreas Symeonidis ^1^**

^1^  Department of Electrical and Computer Engineering, Engineering School, Aristotle University of Thessaloniki, Thessaloniki, Greece
^2^  Laboratory of Psychology, Department of Experimental and Cognitive Psychology, School of Philosophy, Aristotle University of Thessaloniki, Thessaloniki, Greece
^3^  Laboratory of Neurodegenerative Diseases (LND), Center of Interdisciplinary Research and Innovation (CIRI-AUTH), Thessaloniki, Greece
^4^  Greek Association of Alzheimer’s Disease and Related Disorders (GAADRD/Alzheimer Hellas), Thessaloniki, Greece

**Abstract: Background**: Dementia affects millions of people worldwide, causing significant difficulties in their daily lives. The need to create dementia-friendly spaces, specifically adapted to each patient, is crucial, as they can significantly improve the quality of life and functionality. The objective was the development of a platform for assessing indoor spaces and objects in terms of their suitability for individuals with dementia. **Methods:** The focus was placed on categorizing rugs given as input photos based on their suitability for individuals with dementia by implementing a detection algorithm (identification and classification of rugs) and a classification algorithm (categorization only). The detection algorithm was based on the YOLOv7 network, retrained using 2292 rug images. For classification, the ResNet101 network was used, retrained on 2592 rug images. **Results:** The detection algorithm demonstrated low performance, whereas the classification algorithm achieved better results. Specifically, the best prediction performance was obtained using the classification algorithm with KFold validation, achieving an accuracy rate of 90%. **Conclusions:** A web-based platform was developed, allowing users to upload images for semi-automatic assessment of space suitability for individuals with dementia. This can be further expanded to detect additional objects/states, making the evaluation more comprehensive.

**Keywords:** machine learning; image processing; friendly spaces for people with dementia

### 2.76. A Comparative Study of MCI Participants with Mood Disorders in the Language Training

**Alexandra Diamantidou ^1^ and Magda Tsolaki ^2^**

^1^  Psychologist, Greek Association of Alzheimer’s Disease and Related Disorders
^2^  Emeritus Professor of Neurology

**Abstract: Background:** Research has focused on identifying interventions that may slow the progression of the disease and on developing strategies in daily life that can delay the onset or reduce the risk of Alzheimer’s disease. For this reason, we need to know what functions each intervention performs and whether it helps to improve the mental state and mood of the participants. Cognitive training (CT), the most popular non-pharmacological intervention, appears to be the most appropriate method for people with Mild Cognitive Impairment (MCI). We know from previous research that the Language Training improves language functions, in terms of speech comprehension and production. With this study we want to focus on whether or not the mood of the participants in this particular intervention improved. The program is carried out in two ways: written and oral. **Methods:** Only 71 individuals completed the assessments and participated in the analyses, 22 men and 49 women. The program consists of three levels and each level has 24 sessions. Therefore, both people with a high educational level and people with a low educational level can participate.

**Keywords:** Alzheimer; speech; mood disorder

### 2.77. Cognitive Training Through Learning Ancient Greek, Results of a Pilot Study in Older Adults with Mild Cognitive Impairment (MCI)

**Ourania Chatziroumpi ^3^, Eleni Poptsi ^1,2,3^ and Magda Tsolaki ^2,3^**

^1^  Laboratory of Psychology, School of Cognition, Brain and Behavior, School of Psychology, Aristotle University of Thessaloniki (AUTh), 54124 Thessaloniki, Greece
^2^  Laboratory of Neurodegenerative Diseases, Center for Interdisciplinary Research and Innovation, Aristotle University of Thessaloniki (CIRI—AUTh), 54124 Thessaloniki, Greece
^3^  Greek Association of Alzheimer’s Disease and Related Disorders (GAADRD), Petrou Sindika 13 Str., 54643 Thessaloniki, Greece

**Abstract: Background**: Learning a language requires the use of extensive neural networks and this can be a powerful tool for neural reorganization. The present study aimed to determine whether learning Ancient Greek can improve cognitive outcomes of older adults with MCI. **Methods:** 20 individuals (n = 20) were classified in 2 groups: (a) experimental (n = 7) and (b) passive control (n = 13). Groups did not differ significantly in global cognitive function, global executive function, daily functioning and demographic characteristics. The experimental group completed 24 weekly sessions, lasting 6 months. The control group did not participate in any intervention. **Results**: Compared to the control group, the experimental group had better performance in episodic verbal memory (*p* = 0.030) and in learning ability (*p* = 0.046), while showing a trend of improvement in daily functioning (*p* = 0.056). At the end of intervention, the experimental group improved in working memory (*p* = 0.042), visual perception (*p* = 0.018) and episodic visual memory (*p* = 0.042). Control group showed deterioration in visual perception (*p* = 0.040). **Conclusions:** Cognitive training through learning Ancient Greek can lead to improvements in episodic memory, visual perception and executive function.

**Keywords:** mild cognitive impairment; language; ancient Greek; neuroplasticity

### 2.78. Personalised Intervention for People with Multiple Sclerosis: The Experience of Supportive Psychotherapy

**Ioanna Provata, Scientific Associate, Mental Health Counselor and Effrosyni Koutsouraki, Associate Professor of Neurology–Neuroimmunology**

First Department of Neurology, AHEPA Hospital, Aristotle University of Thessaloniki, Greece

**Abstract: Background**: Modern day literature has highlighted the importance of psychosocial interventions for people with Multiple Sclerosis. The aim of this paper is to outline a personalised intervention program of supportive psychotherapy for people with Multiple Sclerosis. It aims to explore the in-depth experience of participants in supportive psychotherapy sessions. Furthermore, it’s goal is to examine the impact of individual psychotherapy regarding the management of a chronic disease. **Methods:** Four semi-structured interviews were conducted with individuals participating in the program. The data sourced from the interviews were then analysed using the Interpretive Phenomenological Analysis method. **Results:** After completing the analysis, three subordinate themes were identified: (a) the therapeutic framework, (b) the journey of psychotherapy, and (c) shielding Multiple Sclerosis. Initially the results highlighted the most important factors in the therapeutic framework that need to be considered when developing these programs. Next, the stages of the therapeutic process and the correlation between the therapeutic relationship and the therapeutic outcome were presented. **Conclusions:** The findings suggest that psychotherapy can act as a protective factor against Multiple Sclerosis, as it can contribute significantly to managing the challenges of chronic illness and improving the quality of life. These findings are significant for both theoretical and clinical purposes.

**Keywords:** multiple sclerosis; psychotherapy; therapeutic relationship; psychosocial intervention

### 2.79. Pilot Therapeutic Program:Cognitive Exercise via Digital Pictures in People with Mild Dementia

**Chrysoula Papasozomenou ^1^**, Evaggelia Bakoglidou ^1^, Eleni Poptsi ^1,2,3^ and Magdalini Tsolaki ^1,3^****

^1^  Greek Association of Alzheimer’s Disease and Related Disorders (GAADRD), Petrou Sindika 13 Str., 54643 Thessaloniki, Greece
^2^  Laboratory of Psychology, School of Cognition, Brain and Behavior, School of Psychology, Aristotle University of Thessaloniki (AUTh), 54124 Thessaloniki, Greece
^3^  Laboratory of Neurodegenerative Diseases, Center for Interdisciplinary Research and Innovation, Aristotle University of Thessaloniki (CIRI—AUTh), 54124 Thessaloniki, Greece

**Abstract: Background:** Non-pharmacological interventions in dementia aim to maintain cognitive abilities and are likely to delay disease progression. **Objective:** Systematic oral and written practice of visual recognition, semantic and phonemic naming, selective visual attention, categorization, visuospatial abilities, aspects of executive function, and delayed verbal and visual recall of total tasks, through either in person or online administration. **Method:** One hundred people with mild dementia of any etiology will be randomly assigned in two groups. The experimental group (N = 50) will attend one-hour sessions, four times per week for six months. The active control group (N = 50), will be engaged in a non-structured conversation. Each session consists of structured cognitive tasks comprising ten digital images depicting a broader semantic or phonemic category. The first exercise comprises naming of these pictures and the participants record the ten words. Selective visual attention tasks are given, linked to the above words (such as identifying words that end in specific letters). Also, participants are asked to categorize these words semantically and then phonetically. A digital drawing related to the session’s theme topic follows. Participants copy and color it as instructed, to practice visual-spatial abilities. At the end of the session, they are asked to verbally recall the assigned tasks (with semantic assistance when deemed necessary). The particular low-cost and interactive intervention initially conducted in a one-hour session per week and combined with additional cognitive programs, as the primary goal was to monitor the administration of the tasks. **Expected Results:** The expected results concern the enhancement of multiple cognitive abilities and the maintenance, as far as possible, of daily functionality.

**Keywords:** cognitive exercise; dementia; pictures; interactivity

### 2.80. Intervention Using Creative Activities in Advanced Dementia

**Agoritsa Karatairi ^1^ and Magdalini Tsolaki ^1,2^**

^1^  Greek Association of Alzheimer’s Disease and Related Disorders (GAADRD), Petrou Sindika 13 Str., 54643 Thessaloniki, Greece
^2^  Laboratory of Neurodegenerative Diseases, Center for Interdisciplinary Research and Innovation, Aristotle University of Thessaloniki (CIRI—AUTh), 54124 Thessaloniki, Greece

**Abstract: Background**: Individuals with advanced dementia often struggle to communicate verbally and have a limited ability to perform even simple tasks. As the disease progresses, a tendency for a “return” to childhood is observed—a process known as regression or retrogenesis. According to Reisberg, the degenerative mechanisms of dementia reverse the natural development of a person. Consequently, cognitive decline in dementia appears to mirror Piaget’s developmental stages in reverse. **Methods:** The intervention aims to stimulate cognition by combining a series of enjoyable group activities. The goal is to alleviate behavioral symptoms, encourage social interactions and artistic expression and evoke positive emotions in the present moment. Therefore, it includes activities aligned with the cognitive age of elderly participants (retrogenetic model). The program includes individuals with an MMSE score of ≤10, corresponding to a cognitive age of under 4 years, with low functionality and behavioral symptoms (apathy, disinhibition, irritability, etc.). Participants utilize visual, tactile, and auditory stimuli—such as drawings, colors, modeling clay, puzzles, beads, baby dolls, musical instruments, and familiar songs—to perform specific cognitive tasks. **Conclusions:** The intervention’s usefulness lies in employing ecological tools and making productive use of time for the benefit of both the patient and their caregiver.

**Keywords:** retrogenesis; Reisberg; Piaget; developmental stages; creative engagement

### 2.81. Therapeutic Program: Cognitive Training Through Cinema

**Evangelia Bakoglidou ^1^, Themis Parastatidis ^1^, Eleni Poptsi ^1,2,3^ and Magdalini Tsolaki ^1,2^**

^1^  Greek Association of Alzheimer’s Disease and Related Disorders (GAADRD), Petrou Sindika 13 Str., 54643 Thessaloniki, Greece
^2^  Laboratory of Psychology, School of Cognition, Brain and Behavior, School of Psychology, Aristotle University of Thessaloniki (AUTh), 54124 Thessaloniki, Greece
^3^  Laboratory of Neurodegenerative Diseases, Center for Interdisciplinary Research and Innovation, Aristotle University of Thessaloniki (CIRI—AUTh), 54124 Thessaloniki, Greece

**Abstract: Background:** The term “cinema-therapy” refers to the use of movies to address psychological issues. Moreover, cinema can positively impact cognitive function by providing rich audiovisual stimuli. **Aim**: (a) to create an innovative cognitive intervention for individuals with Mild Cognitive Impairment (MCI) through movie-watching at the cinema, (b) to train holistic cognitive skills using structured cognitive tasks related to the movie-watching experience. **Method:** In this intervention, participants will initially be guided on how to watch either a movie or a TV series at home. Then, in collaboration with a therapist, they will practice five cognitive skills using memory strategies. In the first part, they answer comprehension and memory questions related to actors, facts, and the plot of the movie. The second one, refers to critical thinking by reflecting on the movie’s messages. In the third, they perform exercises related to attention and visual perception by analyzing picture frames from the movie. The fourth one is based on executive functions (theories of mind), focusing on the emotions expressed through the protagonists’ facial expressions and movements. Finally, they practice the memorization of dialogues. **Expected Outcomes:** This non-pharmaceutical intervention is expected to provide psychological and cognitive benefits for elderly individuals with MCI.

**Keywords:** cognitive training; cinema; mild cognitive Impairment

### 2.82. Training of Attention and Executive Functions Through the Use of Visual Stimuli

**Christina Tzalavara ^1^, Eleni Poptsi ^1,2,3^ and Magdalini Tsolaki ^1,3^**

^1^  Greek Association of Alzheimer’s Disease and Related Disorders (GAADRD), Petrou Sindika 13 Str., 54643 Thessaloniki, Greece
^2^  Laboratory of Psychology, School of Cognition, Brain and Behavior, School of Psychology, Aristotle University of Thessaloniki (AUTh), 54124 Thessaloniki, Greece
^3^  Laboratory of Neurodegenerative Diseases, Center for Interdisciplinary Research and Innovation, Aristotle University of Thessaloniki (CIRI—AUTh), 54124 Thessaloniki, Greece

**Abstract: Background:** Executive functions refer to a set of higher-order cognitive processes responsible for organization, planning, and problem-solving. Research has shown that tasks based on visual stimuli can enhance these functions, supporting the coordination and execution of complex activities. **Methods:** The aim of the proposed study is to design a cognitive training program intended to enhance visual perception, visual memory, selective visual attention, task/rule switching, updating, and working memory processing through the execution of specific visuospatial tasks. Participants will include individuals aged 60 and older, with more than 6 years of education, and diagnosed with Mild Cognitive Impairment (MCI) or mild dementia of any etiology. The intervention will be conducted on an individual basis, lasting 60 min, and will involve pencil-and-paper tasks. The program will consist of eight cognitive tasks, including (a) detecting differences between two seemingly identical images, (b) sequentially connecting numbers, (c) placing objects in specific locations within space, (d) arranging images in order, similar to a puzzle, among others. **Results:** The cognitive program is expected to enhance attention, improve aspects of executive function, and strengthen visuospatial skills. **Conclusions**: Visuospatial tasks can help with the executive functions of the elderly and better coordination of complex activities in daily life.

**Keywords:** executive function; attention; visual tasks; visuospatial skills

### 2.83. Therapeutic Program: Cognitive Training Through Karaoke

**Evangelia Bakoglidou ^1^ and Magdalini Tsolaki ^1,2^**

^1^  Greek Association of Alzheimer’s Disease and Related Disorders (GAADRD), Petrou Sindika 13 Str., 54643 Thessaloniki, Greece
^2^  Laboratory of Neurodegenerative Diseases, Center for Interdisciplinary Research and Innovation, Aristotle University of Thessaloniki (CIRI—AUTh), 54124 Thessaloniki, Greece

**Abstract: Background:** Karaoke (*meaning "empty orchestra"*) is a form of entertainment that, when used wisely within a structured intervention and under the guidance of a well-trained therapist, can become a valuable therapeutic tool. Research on karaoke-based interventions for older adults has shown that it can enhance psychological well-being and social engagement. Furthermore, karaoke may support cognitive functions in older adults with Mild Cognitive Impairment (MCI), as reading lyrics aloud has been shown to improve episodic memory, executive function, and information processing speed. **Aim:** To enhance visual and verbal memory, attention, and critical thinking related to speech. **Method:** Based on these findings, a non-pharmaceutical intervention has been developed for individuals with MCI. In this intervention, a group of older adults sings karaoke while also receiving information about the song’s creators, historical context, and lyrical meaning. Following this, they engage in structured cognitive exercises with a therapist. **Expected Outcomes:** This engaging and creative intervention is expected to contribute to participants’ well-being, strengthen social connections, promote emotional resilience, enhance cognitive skills, and lead to positive changes in quality of life for individuals with MCI.

**Keywords:** cognitive training; karaoke; mild cognitive Impairment

### 2.84. The Nutritional Assessment of Patients of the Greek Association of Alzheimer’s Disease and Related Disorders (Alzheimer Hellas)

**Konstantinos Koullias ^1,2^**

^1^  Aristotle University of Thessaloniki, Faculty of Agriculture, Forestry and Natural Environment, School of Agriculture, Department of Food Science & Technology
^2^  American College of Thessaloniki–Anatolia College, Division of Humanities and Social Sciences, Department of Psychology

**Abstract: Background:** The study evaluated the nutritional assessment of seniors predisposed to Alzheimer’s Disease (AD) or mild dementia, investigating their adherence to the Mediterranean diet and their nutritional status using dietary indices. **Methods:** Participants were provided with two nutritional assessment tools, the Mediterranean Diet Adherence Screener (MEDAS) and the Mini Nutritional Assessment (MNA). The study included elderly men and women with positive and negative A/T/N biomarkers, indicating either a high or a low likelihood of AD, all diagnosed with Mild Cognitive Impairment (MCI). **Results:** Out of 74 individuals, 32 were men (43.24%) and 42 were women (56.76%). Among the men, 9 (12.16%) had positive biomarkers (A+T+N+) and 23 (31.08%) had negative biomarkers (A-T-N-). Similarly, among the women, 17 (22.97%) had positive biomarkers and 25 (33.78%) had negative biomarkers. The mean age was 75.16 years, and the average body mass index (BMI) was 27.854 kg/m^2^. MEDAS scores indicated moderate adherence to the Mediterranean diet (AVG = 9.59), while MNA suggested normal nutrition (AVG = 22.41). **Conclusions:** Women appeared more vulnerable to nutritional deficiencies highlighting the need for further development in the nutritional education of the elderly. No significant differences were found in Mediterranean diet adherence or nutritional status across biomarker groups.

**Keywords:** nutritional assessment; seniors; Alzheimer’s disease; Mediterranean diet

### 2.85. The Effect of Combination of Mediterranean Diet and Oleocanthal in Patients with Mild Cognitive Impairment

Panagiotis-Marios Sotiriadis ^1,2,3,^*, Thomas Tegos ^1^, Prokopios Magiatis ^4^, Emilia Vassilopoulou ^5^, Konstantinos Gerasimidis ^6^ and Magdalini Tsolaki ^1,2,3^

^1^  1st Department of Neurology, “AHEPA” General University Hospital of Thessaloniki, School of Medicine, Faculty of Health Sciences, Aristotle University of Thessaloniki, 54636 Thessaloniki, Makedonia, Greece
^2^  Greek Association of Alzheimer’s Disease and Related Disorders, 54643 Thessaloniki, Makedonia, Greece
^3^  Laboratory of Neurodegenerative Diseases, Center for Interdisciplinary Research and Innovation, Aristotle University of Thessaloniki, 570 01 Thessaloniki, Makedonia, Greece
^4^  Department of Pharmacognosy and Natural Products Chemistry, Faculty of Pharmacy, National and Kapodistrian University of Athens, Panepistimiopolis Zografou, 157 71 Athens, Greece
^5^  Department of Nutritional Sciences and Dietetics, School of Health Sciences, International Hellenic University, 57400 Thessaloniki, Makedonia, Greece
^6^  Human Nutrition, School of Medicine, College of Medical, Veterinary and Life Sciences, University of Glasgow, New Lister Building, Glasgow Royal Infirmary, Glasgow G31 2ER, United Kingdom

**Abstract: background:** Mediterranean diet (MeDi) displays beneficial effects on cognitive function of both healthy individuals and cognitive impaired patients. High phenolic early harvest Extra Virgin Olive Oil (EVOO) is a natural product that contains high concentrations of Oleocanthal, exerting beneficial properties on the cognitive function. **Aim:** To evaluate the combined effect of MeDi and a dietary supplement containing olive oil polyphenols with the main ingredient being oleocanthal (SUPOL) in mild cognitive impairment (MCI) patients. **Methods:** Randomized 4-arm parallel controlled clinical trial, single-blinded for the dietary pattern and double-blinded for the dietary supplement intervention. The intervention duration will be 12 months and the neuropsychological and laboratory evaluation will take place at baseline and after 1-year of intervention. Outcomes include cognitive function and activities of daily living assessment, as well as pathological protein, gut microbiome composition and metabolite production measurement. **Conclusions:** Given the potential of EVOO polyphenols, the goal of the study is to evaluate the efficacy of the combined approach of MeDi and oral SUPOL supplementation compared to MeDi and placebo, the efficacy of MeDi compared to the Western diet and the efficacy of oral SUPOL supplementation compared to placebo on the change in cognition and function in subjects with MCI.

**Keywords:** mediterranean diet; western type diet; oleocanthal; cognition; mild cognitive impairment Alzheimer’s disease

### 2.86. Genetic Counselling Intervention for Relatives of People with Alzheimer’s Disease and Other Dementias in Greece

**Marina Makri ^1,2^, Liana Fidani ^3^, Thomas Tegos ^1^ and Magdalini Tsolaki ^1,2^**

^1^  Department of Neurology, School of Medicine, Faculty of Health Sciences, Aristotle University of Thessaloniki, Thessaloniki, Greece
^2^  Greek Association of Alzheimer’s Disease and Related Disorders, Thessaloniki, Greece
^3^  Department of Medical Biology-Genetics, School of Medicine, Faculty of Health Sciences, Aristotle University of Thessaloniki, Thessaloniki, Greece

**Abstract: Background:** With the increasing demand for clinical genetic services and the lack of clinical geneticists, tele-genetics is utilized in clinical genetics to improve cost efficiency and equitable access to health services in remote areas or for service users experiencing mobility difficulties. However, is tele-genetic counselling (GC) for disclosing APOE genotype for risk of Alzheimer’s disease (AD) equally effective as the onsite interventions? This study examines behavioral and psychological responses following the disclosure of genetic results of AD risk to first-degree relatives of people with AD (PwAD) in Greece. The primary aim was to evaluate the impact of GC on participants’ empowerment, psychological well-being, behavioral adaptations, and risk recall over time. **Methods**: Participants (n = 93) were randomly assigned to one of two GC interventions. Additional grouping variables included the cognitive status (healthy or had mild cognitive impairment-MCI) and the genetic test result (ε4 positive or not). Throughout the three time points (baseline/before GC, T1/3 months post-disclosure, and T2/6 months post-disclosure), participants completed the 21-item Beck Depression Inventory, the Short Anxiety Screening Test, the Impact of Event Scale, the Genetic Counselling Outcome Scale, risk recall, and behavioral questions. Repeated-measures ANOVA, Mann-Whitney U tests, logistic regression, Friedman test, and chi-square tests were used to examine changes in different scores. **Results:** 93 adults (mean age 64.77, range 37 to 83 years, 61% female, mean years of education 13.72) were randomly assigned to online (46 participants) and onsite (47 participants) groups. There were no statistically significant differences between the groups in GCOS-24, SAST, BDI, and IES-R scores regardless of the time as well as between healthy and MCI participants. However, people with MCI have substantially lower odds of recalling risk after six months compared to healthy participants. People with ε4 were much more likely to endorse behavior change and indicated higher scores in IES-R. **Conclusions:** Tele-genetic counselling can serve as an effective intervention for APOE disclosure for healthy relatives of PwAD and people with MCI.

**Keywords:** APOE; Alzheimer disease; genetic testing; psychological reaction; health-behavior modification

### 2.87. Non-Pharmacological Approaches to Managing Behavioral and Psychological Symptoms of Dementia (BPSD)—Challenges and Limitations

**Georgia Batsila and Ourania Chatziroumpi**

Greek Association of Alzheimer’s Disease and Related Disorders (GAADRD), Petrou Sindika 13 Str., 54643 Thessaloniki, Greece

**Abstract: Background**: Dementia is an acquired loss of cognition in multiple cognitive domains. As the disease progresses, in addition to cognitive difficulties, the patient may experience behavioral and emotional changes. The term “Behavioral and Psychological Symptoms of Dementia” (BPSD) include but are not limited to apathy, depression, anxiety, psychosis, agitation, aggression, sleep disturbances, and other behaviors such as wandering, sexually inappropriate behaviors, and care refusal. These symptoms may be an early sign of impending dementia or may manifest in the course of the disease. Available treatments: a) non-pharmacological interventions (sensory stimulation, sensory input and stress reduction, reminiscence therapy, physical exercise, animal-assisted therapy, puppet therapy, etc.), which should be first-line when the primary causes are due to environmental factors and b) pharmacological interventions (neuroleptics, SSRIs, benzodiazepines, etc.), which are the first choice treatment when the primary causes are related to brain changes. The management of BPSD is a major challenge due to their inherent complexity, it must be individualized and holistic, as it significantly affects the quality of life of both patients and their caregivers.

**Keywords:** non-pharmacological; dementia; behavioral disorders

### 2.88. The Effect of Doll Therapy on Patients with Moderate-Stage Dementia: Theoretical Framework and Applications at the "Agia Eleni" Day Center

**Agoritsa Karatairi ^1^, Georgia Batsila ^1^ and Magdalini Tsolaki ^1,2^**

^1^  Greek Association of Alzheimer’s Disease and Related Disorders (GAADRD), Petrou Sindika 13 Str., 54643 Thessaloniki, Greece
^2^  Laboratory of Neurodegenerative Diseases, Center for Interdisciplinary Research and Innovation, Aristotle University of Thessaloniki (CIRI—AUTh), 54124 Thessaloniki, Greece

**Abstract: Background**: Cognitive deficits in dementia are often accompanied with changes in personality, behavior, and social engagement. Doll Therapy is a non-pharmacological intervention that utilizes baby dolls as therapeutic tools. According to research literature, it aims to minimize the psychological and behavioral symptoms of individuals with dementia while improving their emotional well-being. **Methods:** The program includes individuals with an MMSE score of ≤10, corresponding to a cognitive age of under 4 years, who exhibit behavioral disturbances, most commonly apathy, depression, and aimless wandering. Each participant is provided with a doll and, under the psychologist’s guidance, is encouraged to take care of it. This can be expressed through non- verbal communication (smiling), movement (hugging), the use of objects (baby bottle), or engagement in specific activities (putting the doll to sleep, dancing). **Conclusions:** Participants appear to benefit from the evocation of pleasant emotions and the reduction of disruptive behaviors, while caregivers experience relief from the constant demands of caring for their loved ones.

**Keywords:** doll therapy; behavioral symptoms; emotional well-being; caregiving

### 2.89. The Effect of Doll Therapy on Patients with End-Stage Dementia: Results from the Implementation of the Intervention at the "Panagia i Glykophilousa" Palliative Care Unit

**Aikaterini Boutikou ^1^ and Magdalini Tsolaki ^2^**

^1^  Social Worker, GAARD, Palliative Care Unit “Panagia Glykofilousa” Thessaloniki, Nik. Plastira 65, 54250, Greece
^2^  Neurologist/Psychiatrist & Theologist, GAARD, Palliative Care Unit “Panagia Glykofilousa” Thessaloniki, Nik. Plastira 65, 54250, Greece

**Abstract: Background:** Patients with moderate to severe dementia develop at least one behavioral or psychological symptom, such as apathy, anxiety or agitation, which significantly impacts their quality of life. Among the non-pharmacological strategies for managing these symptoms is Doll Therapy (DT). This is a person-centered therapy that involves the patient’s engagement in activities such as speaking to, feeding, or hugging a doll. Bowlby’s attachment theory has been the central principle used to explain the potential benefits of this intervention, as dementia patients experience a continuous loss of control and security, seeking reassurance and protection from those around them. Following this theory, we applied DT, hypothesizing that it could contribute to creating a sense of security and alleviate some of the symptoms. **Methods:** After selecting the patients eligible for the intervention, individualized and structured sessions (using a questionnaire) were designed for each participant. The primary tool used was professional observation. **Results:** Expression of emotions and improvement in communication skills were observed, along with an increased display of pleasure and joy, provision of emotional support, increased engagement and attention, reduction of apathy, and a calming effect. **Conclusions:** DT can be an effective intervention and should be applied to patients with dementia.

**Keywords:** doll therapy; attachment theory; behavioral symptoms; security

### 2.90. Operation of a Cognitive Strengthening Workshop in a Primary Health Care Facility

**Eleni Vorizanou ^1^, Melpomeni Mousafiropoulou ^2^, Despina Aroni ^3^, Panagiota Papadimitriou ^4^ and Agoritsa Koulouri ^5^**

^1^  Nurse, RN, MCS, 1st Health Center of Salamina
^2^  RN, MSC(c), Nurse, 1st Health Center of Salamina
^3^  PhD, M.Phil, M.A, B.A, Social Worker (Psychologist), 1st Health Center of Salamina
^4^  Social Worker, 1st Health Center of Salamina
^5^  PhD, MSc, MHSc, RMHN, RN, Education Coordinator, Nursing Specialty, 2nd DYPE of Piraeus & Aegean, Head Nurse 1st Health Center of Salamina

**Abstract: Introduction:** The number of people exhibiting cognitive impairment is continuously increasing, resulting in significant expenditures from available health resources. Primary Health Care (PHC) is required to address the ever-growing needs regarding dementia care. Cognitive strengthening is a non-pharmacological intervention by healthcare professionals, involving a structured series of therapeutic exercises and functional activities designed for the re-education of cognitive abilities. At the 1st Health Center of Salamina, a cognitive strengthening workshop operates, involving 27 patients with mild cognitive impairment, mild, and moderate dementia. A sample of 9 patients, 7 women and 2 men, was selected for evaluation of the intervention. The tools used were GDS, MMSE, and MoCA tests. **Results:** 89% of individuals showed stability, and 11% showed improvement on the GDS scale. In dementia evaluation, 7 patients showed stability, 1 showed improvement, 1 had a lower score on the MoCA, and 100% of the beneficiaries maintained the same score on the MMSE. **Conclusions:** The results from the operation of the workshop were positive, and the suggested non-pharmacological intervention can contribute to stabilizing or even slowing the progression of the disease.

**Keywords:** dementia; cognitive strengthening; cognitive impairment detection tools; health center

### 2.91. Dementia Management in Primary Health Care—Psychosocial Interventions of the Social Service Office at the 1st Health Care Center of Salamina

**Despoina Aroni ^1^, Panagiota Papadimitriou ^2^ and Agoritsa Koulouri ^3^**

^1^  Social Worker, Psychologist B.A., M.A., M.PhiL., PhD
^2^  Social Worker B.A.
^3^  Head of Nursing Department and Health Professionals PhD, MSc, MHSc, BMHN, RN

**Abstract: Background:** The rapid increase in the proportion of elderly people in the general population, along with the effects of socio-economic, cultural, environmental, and other factors, significantly impact the rise in individuals who will suffer from dementia. As a result, the provision of public health and community care services is deemed necessary, making social work a key pillar of support. The purpose of this study is to present the interventions conducted by the Social Services Office of the 1st Health Care Center of Salamina for the prevention, detection, and management of dementia. **Methodology**: Psychosocial support interventions were carried out at the micro, mezzo, and macro levels for early diagnosis, easy and equitable access, and the linking of individuals and families with health structures, services, and resources. Informational talks and preventive screenings were organized in the local community. Psychometric tests were conducted, and the lived experiences of the individuals, along with the needs and capabilities of their families, were recorded. Their “voice” was strengthened in advocating for their rights, and a Cognitive Empowerment Workshop was organized for the first time within a Primary Health Care structure. **Conclusions:** Social work in Primary Health Care, within the framework of a holistic interdisciplinary approach, plays a crucial role in providing psychosocial interventions for dementia management, raising awareness in the general population, eliminating stigma, and promoting active aging.

**Keywords:** dementia; social work and dementia; active aging; vulnerable groups in the health center

### 2.92. Translation, Cross-Cultural Adaptation, Reliability and Validity of the Greek Version of the Disability Assessment for Dementia Scale (DAD-Gr)

**Georgios Marios Kyriakatis ^1^, Prokopia Mirka Lykou ^1^, Christos Christodoulou ^2^, Zacharias Dimitriadis ^3^ and Thomas Besios ^4^**

^1^  Physiotherapist, Department of Physiotherapy, Human Performance and Rehabilitation Laboratory, School of Health Sciences, University of Thessaly
^2^  Physiotherapist, Department of Physiotherapy, School of Health Sciences, University of Thessaly
^3^  Associate Professor, Department of Physiotherapy, School of Health Sciences, University of Thessaly
^4^  Associate Professor, Department of Physiotherapy, Human Performance and Rehabilitation Laboratory, School of Health Sciences, University of Thessaly

**Abstract: Background**: The Disability Assessment for Dementia (DAD) scale consists of a total of 40 items and assesses both basic and instrumental activities of daily living. **Methods:** The scale was given to caregivers of 30 patients (24 female and 6 male patients with any type of dementia, mean age 82.26 ± 6.80). The assessment of validity performed using the Katz and Lawton scales, while a total of 2 measures were used to check reliability. Statistical analyses were performed to test validity and reliability using Spearman’s correlation coefficient and the Intraclass Correlation Coefficient, respectively. **Results:** The test-retest reliability of the total DAD scores was found excellent (ICC = 0.99). The subscale for the basic and instrumental DAD scale scores was significantly correlated with Katz (r_s_ = 0.79, *p* < 0.001) and Lawton (r_s_ = 0.90, *p* < 0.001) scale scores, respectively. The total DAD scale score was significantly correlated with both Katz (r_s_ = 0.76, *p* < 0.001) and Lawton (r_s_ = 0.92, *p* < 0.001) scale scores. **Conclusions:** The DAD-Gr scale was found to be reliable and valid in the Greek population for the assessment of functional independence in patients with dementia.

**Keywords:** dementia; activities of daily living; functionality; assessment

### 2.93. Capturing Cognitive Decline in Aging Since Its Beginnings: Findings from the Use of the R4Alz-R Battery

**Eleni Poptsi ^1,2,3^, Despina Moraitou ^1,2^, Emmanouil Tsardoulias ^4^, Andreas L. Symeonidis ^4^ and Magda Tsolaki ^2,3^**

^1^  Laboratory of Psychology, School of Cognition, Brain and Behavior, School of Psychology, Aristotle University of Thessaloniki (AUTh), 54124 Thessaloniki, Greece
^2^  Laboratory of Neurodegenerative Diseases, Center for Interdisciplinary Research and Innovation, Aristotle University of Thessaloniki (CIRI—AUTh), 54124 Thessaloniki, Greece
^3^  Greek Association of Alzheimer’s Disease and Related Disorders (GAADRD), Petrou Sindika 13 Str., 54643 Thessaloniki, Greece
^4^  School of Electrical and Computer Engineering, Faculty of Engineering, Aristotle University of Thessaloniki (AUTh), 54124 Thessaloniki, Greece

**Abstract: Background:** Early diagnosis of aging-related neurocognitive disorders, even in asymptomatic phases, is a key goal in neuroscience. The R4Alz-R is a cognitive tool designed to detect subtle cognitive changes associated with aging, from healthy aging to Subjective Cognitive Impairment (SCI) and Mild Cognitive Impairment (MCI), prior to dementia. This study aimed to assess R4Alz-R’s ability to detect these early changes. **Methods:** The study included 184 participants: cognitively healthy young adults (HCya), healthy older adults (HCoa), SCI, and MCI. The R4Alz-R assesses memory storage, information processing, working memory updating, attention, executive functioning (set-shifting, inhibitory control), and episodic memory. **Results:** A two-factor model identified Fluid Intelligence and Episodic Memory (α = 0.78). The Flexibility and Attention scores effectively distinguished HCya from SCI (sensitivity 87.1%, specificity 88.1%) and MCI (sensitivity 85.3%, specificity 97.5%). The Executive Functioning score distinguished HCoa from SCI (sensitivity 91.3%, specificity 87.5%), while Fluid Intelligence discriminated HCoa from MCI (sensitivity 85.7%, specificity 100%). **Conclusions:** Cognitive decline in aging may begin in areas related to cognitive control. Healthy older adults can maintain cognitive levels similar to younger adults, aligning with current aging models. **Conclusions:** The R4Alz-R is a reliable, valid tool for diagnosing early cognitive decline, potentially reversible if detected early.

**Keywords:** aging-related cognitive impairment; cognitive control; neuropsychological tool; very early diagnosis

### 2.94. The Use and Presentation of a Special Lexical Test for the Assessment of Mental Lexicon in Subjects with Subjective Cognitive Decline

**E. Neofytidou ^1,2,3,4^ and M. Tsolaki ^2,3,4^**

^1^  Aristotle University
^2^  1st Department of Neurology, Aristotle University of Thessaloniki
^3^  Alzheimer Hellas
^4^  Laboratory of Neurodegenerative Diseases, Center for Interdisciplinary Research and Innovation (CIRI-AUTH), Balkan Center, Aristotle University of Thessaloniki

**Abstract: Background:** A primary objective of researchers in international and Greek literature is to investigate the role of language, particularly the mental lexicon, in dementia syndromes. The detection and assessment of the mental lexicon are achieved through lexical recognition and decision tasks. **Methods:** This ongoing study aims to evaluate the mental lexicon in elderly individuals and to determine the correlation of its decline, primarily with subjective cognitive decline (SCD), using a lexical test with vocabulary derived from digital corpora of written and spoken language of highly educated elderly individuals (comprising 28 real and 28 non-real words). This specific test aims to examine qualitatively and quantitatively lexical recognition, phonological, and morphological memory in a sample of subjects with pathological findings (reduced levels of β-amyloid in CSF-A+) associated with Alzheimer’s disease, individuals with SCD, and healthy individuals without pathological findings (no reduced β-amyloid-A-). **Results:** The descriptive analysis of a small sample in this research phase [(N = 29, 27 women, mean age 68 years, SD = 7.52, mean education 14.17 years, SD = 3.33, and diagnosis: 11 (37.9%) cognitively healthy, 17 (58.6%) with SCD] showed that all participants correctly identified real words, but not non-real words. 10 (34.5%) individuals with SCD had low performance, while 8 (27.6%) cognitively healthy individuals had high performance (Cronbach’s = 0.788). A small positive correlation (r = 0.276) was found between diagnosis type and subject performance. Analysis of variance indicated that CSF β-amyloid measurement significantly affected subject performance on the mental lexicon test. Conclusions: International literature has established a positive correlation between the mental lexicon and cognitive decline, which this study also supports. The ultimate goal is to investigate a larger subject sample to obtain statistically significant data on the validity and reliability of the lexical test for assessing the relationship between the mental lexicon and dementia syndromes.

**Keywords:** mental lexicon; lexical test; subjective cognitive decline

### 2.95. Towards an Automated Early Detection: Evaluation of LANGaware’s Language and Speech Biomarkers for Neurocognitive and Emotional Disorders

**Vassiliki Rentoumi ^1^, Evangelos Vasiliou ^2^, George Paliouras ^3^, Dimitra Sali ^1^ and Admir Demiraj ^1^**

^1^  LANGaware
^2^  University of the Aegean
^3^  SKEL AI lab, Demokritos

**Abstract: Background:** Recent developments in language analysis using machine learning (ML) methods highlight the effectiveness of digital biomarkers in detecting changes in cognitive status. Although the distinction between Alzheimer’s disease (AD) and control groups has been researched, the classification of mild cognitive impairment (MCI) remains difficult due to the multiple factors involved. **Methods:** We assessed the LANGaware biomarkers through experimental design for: (a) distinguishing dementia from control, (b) distinguishing dementia, MCI, and control, and (c) distinguishing depression/anxiety from control. We used patient recordings and transcribed texts, extracting linguistic and acoustic features analyzed with a neural network. **Results:** The data were split into a training set (70%) and a test set (30%). The model achieved: (a) 89% accuracy and 85% F1 score for dementia versus control, (b) 70% accuracy and 71% F1 score for dementia, MCI, and control, (c) 71% accuracy and 71% F1 score for distinguishing depression/anxiety from control. **Conclusions:** Our approach successfully distinguishes individuals with MCI from those with dementia or healthy controls (control group), contributing to automated assessment and early diagnosis, with application also in detecting emotional disorders such as depression and anxiety.

**Keywords:** artificial intelligence; non-invasive detection; early detection; digital biomarkers

### 2.96. Digital Dementia: Forms and Progression

**Anna Dimotaki**

Institute for Research and Education for Psychiatric and Dementia Patients, Chania, Greece

**Abstract:** The term “digital dementia” was introduced by the German neuroscientist Manfred Spitzer and refers to the long-term consequences of the improper use of new technologies and social media, primarily at a cognitive level. Digital dementia refers to alarming symptoms that may appear due to excessive use of new technologies/the virtual world, which are related to memory loss, attention and thinking disorders, low motivation levels, lack of problem-solving skills, emotional difficulties, and communication problems. The death of nerve cells begins before the individual becomes aware of the aforementioned symptoms of the “disease”. Its progression rate depends on the cognitive development level of the brain before cognitive decline begins. The greater the individual’s cognitive reserve, the later they will recognize their cognitive decline. Prolonged screen use during the brain’s development phase increases the risk of developing Alzheimer’s disease or other forms of dementia in adulthood. Phenomena related to digital dementia include problematic use of social media, the need for constant checking of the internet, continuous use of mobile devices during social interactions, and even while walking on the street. Nowadays, the goal is rather the rational use of new technologies than their avoidance.

**Keywords:** digital dementia; new technologies; social media; mobile devices

### 2.97. Dementia and the Importance of Religiosity in the Greek Christian Orthodox Context Social and Ethical Dimensions

**Eftychia Lazarou ^1^, Christos Tsironis ^2^, Vasileios Kalliakmanis ^2^ and Magda Tsolaki ^3^**

^1^  MSc, Philologist, GAARD, Palliative Care Unit “Panagia Glykofilousa” Thessaloniki, Nik. Plastira 65, Greece
^2^  PhD, School of Theology, Department of Ethics and Sociology, Aristotle University of Thessaloniki, Greece
^3^  MD, PhD, Neurologist/Psychiatrist & Theologist, GAARD, Palliative Care Unit “Panagia Glykofilousa” Thessaloniki, Nik. Plastira 65, Greece, President of the Panhellenic Federation of Alzheimer’s disease, Coordinator of the Laboratory of Neurodegenerative Diseases (KEDEK)

**Abstract: Background:** It has been repeatedly found that the participation of elderly people in religious activities that stimulate brain functions helps them to strengthen and develop these functions, as well as to protect them from further worsening either due to age or neurodegenerative diseases. The purpose of this study is to explore the possible correlation of religiosity, in Patients with Mild Cognitive Impairment (MCI), Alzheimer’s Disease and Dementia. Furthermore, it will analyze the social and ethical implications of the issue. A review of the literature indicates that the religiosity has positive effects on the prevention and treatment of dementia. It is well known that for many patients with chronic and life-threatening illnesses such as dementia, it is important to meet both spiritual and religious needs, as well as biological and physical needs. **Methods:** The aim of this study is to evaluate the positive effect of religiosity on 300 patients with Alzheimer Disease and dementia, Mild Cognitive Impairment (MCI) compared with the appropriate questionnaire. **Results:** This is ongoing research and therefore the final results are expected within the next year. **Conclusions:** It is understood that meeting the religious needs of people with chronic and life-threatening illnesses, such as incurable diseases, needs to be given immediate priority in the holistic approach to care and to be taken into account in the design and implementation of health care.

**Keywords:** dementia; religiosity; Alzheimer’s disease; prevention

### 2.98. The Role of Psychotherapy in the Emotional State of Patients with Mild Cognitive Impairment

**Sofia Angeliki Michopoulou ^1,2^, Glykeria Tsentidou ^2^ and Magda Tsolaki ^3^**

^1^  Cognitive Neuropsychology, University of East London, E16 2RD, United Kingdom
^2^  Day Center for Dementia “Panagia i Vimatarissa”, 601 00 Katerini, Greece
^3^  Greek Association of Alzheimer’s Disease and Related Disorders, 546 43 Thessaloniki, Greece

**Abstract: Background**: Mild Cognitive Impairment (MCI), in addition to the expected cognitive decline, is often accompanied by psychological symptoms such as depression and anxiety, which exacerbate patients’ difficulties. The aim of this longitudinal study was to compare the emotional state of MCI patients who participated in psychotherapy as part of an intervention program with those who did not. **Methods:** The study followed an experimental design and included a sample of 47 individuals, with a mean age of 70.2 years. The analysis was conducted using repeated measures at two time points, while the BDI (Beck Depression Inventory) and SAST (Short Anxiety Screening Test) scales were used for evaluation. **Results:** (i) patients who underwent psychotherapy were, on average, younger and better educated, (ii) a tendency for improvement in anxiety was observed in the psychotherapy group, but without a statistically significant difference, (iii) high anxiety during the first neuropsychological assessment appeared to be associated with reduced effectiveness of psychotherapy. **Conclusions:** The above data highlight the role of demographic factors and initial anxiety levels in the patients’ response to psychotherapy, emphasizing the importance of tailored interventions for more substantial improvement.

**Keywords:** mild cognitive impairment; anxiety; depression; psychotherapy; intervention

### 2.99. Needs Assessment Protocol for Dementia Patients and Caregivers—Developing a Therapeutic Plan: Athens Alzheimer Association

**Eirini Vamvakari**

MSc, Social Worker, Scientific Supervisor, Mobile Dementia Unit of Piraeus, Athens Alzheimer Association, Greece

**Abstract:** The irreversible demographic aging that is taking place worldwide will lead to a significant increase in dementia patients in the next years with the expected impact. Dementia is a neurodegenerative disease that affects mental functions, memory, thinking, speech, visual-spatial perception and behavior, progressively leading the patient to the loss of personal autonomy with a major impact on the quality of life both to patient and people involved in his/her care. Given the fact that there is still no effective etiological treatment for the disease, the goal of care remains to ensure a “good quality of life” for people with dementia and their caregivers. At the Athens Alzheimer Association, with a view to early diagnosis of dementia and its effective management, we have developed a needs assessment protocol which is completed in every patients’ visit by members of the interdisciplinary team with the ultimate goal of developing an individualized therapeutic plan. The data for completing the patient needs recording and the final determination of the treatment plan are acquired through the tests & information collected during the medical examination, neuropsychological tests, social work intake, speech therapy evaluation, information on physical health - functionality and general physical condition.

**Keywords:** needs assessment; dementia patients; caregivers; therapeutic plan

### 2.100. The Role of Narratives in People with Dementia in a Closed Care Unit

**Maria Manta, Psychologist and Eleni Kampoura-Nifli, RN, MA, Nursing and president of the E.E.N.A.L.**

Hellenic Society for Alzheimer’s Disease and Related Disorders of the Prefecture of Larissa

**Abstract: Background:** to improve memory, attention, speech, perception, mood and quality of life. **Methods:** 31 participants, 21 women and 10 men, aged 55–85, of varying educational level participated. Subjects were assessed with the following cognitive tools, M.M.S.E., G.D.S., BAI, and Quality in life in AD. In addition, the intervention plan, the narrative material and the procedure, to which the beneficiaries agreed, were presented. The storytelling, took place in the form of two-hour workshops weekly for 6 months. Every other week the narrative material was repeated with parallel discussion in order to consolidate the content of the narrative and at the same time to give the beneficiaries the opportunity to make parallel narratives. The narrative material included short stories, poems and a fairy tale. After the intervention was completed, the same tools were used to re-evaluate and assess the observational material. **Results:** Overall, improvements in memory, language, attention, perception, mood and communication were demonstrated. **Conclusions:** Storytelling is a useful tool, for empowerment of people living in closed care units, deprived of communication and experiencing loneliness.

**Keywords:** dementia; narratives; closed unit

### 2.101. Choreomotor Intervention in People with Dementia

**Maria-Magdalini Koutoula, Choreographer, Ioanna Manta, Business Administration, Maria Manta, Psychologist and Eleni Kampoura-Nifli, RN, MA, Nursing, president of the E.E.N.A.L.**

Hellenic Society for Alzheimer’s Disease and Related Disorders of the Prefecture of Larissa

**Abstract: Background:** The purpose of applying choreokinetics was to stimulate the mind, harmonize mind and body, improve mood, increase self-confidence and extroversion. **Methods:** Fifty-two women, of 5 groups, aged 60–82, of varying educational levels, participated. They were briefed by the choreographer on the process, the choice of musical sounds and the use of auxiliary tools, with which they agreed. In addition, four-point balance, cognitive and emotional state tests, with Moca, M.M.S.E, FRSSD, IADL, G.D.S and BAI, were administered before and after. This was followed by the six-month training, with two weekly workshops. The aim of the workshops was to transform musical rhythm into choreography. **Results:** At the end of the intervention and the evaluation of all the material, the following emerged: All groups improved memory, speech, coordination and mood. They expressed feelings of joy, self-confidence and showed social interaction 4 of the 5 groups performed their choreographies of choice at a very good level, while 1 of the 5, at a excellent level. The difference in performance was due to the multiculturalism of the group and the competitiveness. **Conclusions:** Choreokinesis is inspiring and beneficial when individuals’ wants are identified with exciting musical sounds.

**Keywords:** dance kinetics intervention dementia; dementia; Individuals

### 2.102. Neurofeedback as a Tool in Stroke Rehabilitation

**Sotirios Mpanias Drosos ^1^ and Dimitra Stavridou ^2^**

^1^  Neuropsychologist, Psychologist, Athens’ Neurofeedback Center
^2^  Psychologist, Graduate of the Neuropsychology Department, SCG, Athens’ Neurofeedback Center

**Abstract: Background:** The disruption of cerebral blood flow can lead to a stroke. The resulting deficits from a stroke depend on the affected brain region deprived of blood supply. Significant impairments after a stroke are observed in the motor system. The aim of this abstract is to investigate the role of neurofeedback in stroke rehabilitation. **Methods:** This abstract includes three quantitative studies that utilized neurofeedback as a method to improve stroke patients’ conditions. In a study, two participants (a 63-year-old man and a 77-year-old woman) with chronic ischemic stroke underwent 15 neurofeedback sessions over two months. In another study by the same researchers in 2019, a 61-year-old patient with chronic stroke and right hemiparesis participated in eight consecutive neurofeedback sessions over eight days. Lastly, in a third study, three stroke patients received neurofeedback sessions every two days for four weeks. **Results:** In the first study, results showed a reduction in anxiety and depression in both patients. Specifically, the man exhibited improved speech and verbal fluency, while the woman showed enhanced gait ability. In the second study, the patient who used neurofeedback as a rehabilitation method demonstrated improvements in motor function. The last study, revealed that neurofeedback helped improve upper limb mobility in stroke patients. **Conclusions:** Overall, neurofeedback appears to contribute to the rehabilitation of motor function and the improvement of the emotional state of stroke patients. Future research is recommended to further explore the effectiveness of neurofeedback as a rehabilitation tool in other neurological conditions.

**Keywords:** Νeurofeedback; Stroke; Stroke rehabilitation; EEG

### 2.103. Intergenerational Connection: Non-Pharmacological Interventions for Psychosocial and Cognitive Enhancement

**Eleni Antoniadou ^1^, Marianna Tsatali ^2,3^, Vasilis Psaltis ^2^, Dimitris Sarris ^1^ and Magda Tsolaki ^2,4,5^**

^1^  Pedagogical Department of Early Childhood Education, University of Ioannina, Epirus, Greece
^2^  Alzheimer Hellas, Thessaloniki, Makedonia, Greece
^3^  Laboratory of Psychology, Department of Cognition, Brain and Behavior, School of Psychology, Aristotle University of Thessaloniki (AUTh), 54124 Thessaloniki, Greece
^4^  Laboratory of Neurodegenerative Diseases, Center for Interdisciplinary Research and Innovation, Aristotle University of Thessaloniki (CIRI-AUTh), 54124 Thessaloniki, Greece
^5^  1st Department of Neurology, Medical School, Faculty of Health Sciences, Aristotle University of Thessaloniki

**Abstract: Background**: This symposium explored intergenerational connections based on developmental benefits for both children and older adults. It examined age-related stereotypes and the overall effectiveness of intergenerational interventions. Additionally, an intergenerational educational program was presented. **Methods:** The study included 27 former teachers, equally divided into three groups: (a) an experimental group participating in the intergenerational intervention, (b) a control group attending cognitive enhancement programs, and (c) a control group receiving no intervention. All participants were beneficiaries of the Greek Association of Alzheimer’s Disease and Related Disorders. Additionally, 18 second-grade students were assigned to either (a) an experimental group participating in the intergenerational program or (b) a control group. The intervention lasted three months, involving three weekly sessions covering language and mathematics. Neuropsychological assessments were conducted for both older adults and children, and children participated in two focus groups as a qualitative measure. **Results:** Quantitative analysis revealed significant improvements in reading abilities among children in the experimental group post-intervention. However, no statistically significant differences were found among educators. Qualitative analysis indicated that children’s attitudes toward older adults became more positive. **Conclusions:** The results highlight the effectiveness of intergenerational interventions in enhancing children’s academic and social attitudes, while older adults benefited indirectly from the positive shift in students’ perceptions.

**Keywords:** intergenerational connection; cognitive enhancement; ageing; development; ageing stereotypes

**Funding:** Funding was received from John S. Latsis Public Benefit Foundation.

### 2.104. Elder Abuse with Dementia: Risk Factors-Prevention and Confrontation

**Vasiliki Pattakou-Parasyri**

Social Worker, Department of Social Work Hellenic Mediterranean University, Alzheimer Hellas

**Abstract: Background:** Since elderly population increases worldwide, dementia rates also increase. Older people with dementia are at higher risk of abuse, although further research on this topic has been neglected. **Methods:** This literature review’s purpose is to identify risk, prevention and handling factors of elder abuse with dementia. **Results:** Although opposing risk factors of abuse are often described, mainly due to cultural differences, most studies on this area resulted in similar findings. The abuse rates are extremely high; up to 62.3% or 78.3% depending on where and when the survey was conducted. During the COVID-19 pandemic period, the elder abuse rates were remarkably high, compared to the elderly without dementia. The primary risk factor was the level of cognitive ability and the stage of dementia along with functionality. Another risk factor involves behavioural problems and psychological/psychiatric issues. A further risk factor is the nature of the patient-caregiver relationship before and after dementia. Finally, for the caregivers, a risk factor is psychological and psychiatric problems and the degree of burden and duration of care. **Conclusions:** Interdisciplinary cooperation of professionals and institutions is proposed, after appropriate training and development of effective protocols in order to protect human rights and ensure a good quality of life.

**Keywords:** elder abuse; people with dementia; interventions; prevention

### 2.105. An Innovative Educational Program for Genetic Counseling in Neurodegenerative Diseases Developed by Greece, Germany, Belgium, Spain and Turkey

**Marina Makri ^1,2^, Ioanna Antigoni Angelidou ^3^, Sebastiaan Engelborghs ^4^, Magdalini Tsolaki ^1,2^, Görsev Yener ^5^, Joke Temmerman ^4^, Birgit Teichmann ^3^, Akyllina Despoti ^6^, Andrea Miguel ^7^ and Victoria Fernández ^7^**

^1^  Department of Neurology, School of Medicine, Faculty of Health Sciences, Aristotle University of Thessaloniki, Thessaloniki, Greece
^2^  Greek Association of Alzheimer Disease and Related Disorders, Thessaloniki, Greece
^3^  Network Aging Research (NAR), Heidelberg University, Heidelberg, Germany
^4^  Department of Neurology and Center for Neurosciences, UZ Brussel and Vrije Universiteit Brussel, Brussels, Belgium
^5^  Faculty of Medicine, Izmir University of Economics, Izmir, Turkey
^6^  Clinical Ergospirometry, Exercise and Rehabilitation Lab, School of Medicine, National and Kapodistrian, University of Athens, Athens, Greece
^7^  Research Center and Memory Clinic, Fundació ACE, Institut Català de Neurociències Aplicades, Universitat Internacional de Catalunya, Barcelona, Spain.

**Abstract: Background:** Nowadays, with the rising prevalence of neurodegenerative diseases (ND) and the accessibility of genetic testing, it is expected that there would be a greater need for high-quality genetic information on ND, most likely provided in the form of genetic counseling (GC) from qualified healthcare professionals (HCP). **Objectives:** The GECONEU project developed an online Course focusing on GC on ND for University students and healthcare professionals. This project’s main goals and central impact were to support people, caregivers, and society to better understand the aims of genetic testing and the usefulness of GC. **Results**: The three primary outcomes of this initiative are as follows: 1. “Transnational Implementation Guidelines for GC in ND” focused on GC intervention steps. This result combined different GC protocols as well as a quantitative survey on families of people with ND (PwND), and qualitative research on experts in the field; 2. “An innovative online course on GC in ND” that can be part of the curriculum of Higher Education Institutions (HEI); 3. “A digital Guideline handbook” that helps HEIs and other care organizations to implement the training, including also proposals and directions on how to extend the knowledge on GC. This result was based on extensive testing of the teaching material and the course in pilots in 5 different countries. **Conclusions**: Genetic testing for ND has become more significant in personalized treatment and disease risk reduction during the past 20 years, particularly for healthy people with a family history of ND. By including the families of PwND in the development of the educational course, this project offered the chance to consider all the components of a suitable training program for society, thereby increasing their visibility and raising their genetics knowledge.

**Keywords:** genetics; genetic counseling; neurodegenerative disorders; University course

**Funding:** The project is co-funded by Erasmus+ Programme of the European Union under grant agreement No. 2021-1-EL01-KA220-HED-000032173.

### 2.106. Mapping New Paths: Exploring the Profile of the Participants of the Memory Clinic of the Association “Frodizo”

**Dimitris Theodoropoulos ^1^, Maria Frounta ^1,2^, Ioanna Tselepi ^1^, Angeliki Skoura ^1^, Panagiotis Alexopoulos ^2^ and Elli Markaki ^1^**

^1^  Frodizo-Corporation for Succor & Care of Elderly & Disabled, Patras, Greece
^2^  Medical School, University of Patras, Greece

**Abstract: Background**: Effective prevention and treatment of Alzheimer’s disease and other dementia types require structured and specialized interventions. This study aims to describe the demographic and clinical profile of users attending the memory clinic operated by the “Frodizo” Association, examining socio-demographic characteristics, dementia diagnosis, depression prevalence, and utilization of targeted intervention services provided by the clinic and the affiliated Day Centre for dementia care. **Methods**: Data were analyzed using Microsoft Excel for descriptive statistical analysis. **Results**: Between February 2020 and December 2024, the Memory Clinic served 1157 individuals, of whom 70% were women and 30% men. The majority (60%) were aged between 50–70 years, with 88% residing in the greater Patras area. Alzheimer’s disease was the most frequent dementia diagnosis. Among the subset of 251 individuals assessed in detail at the clinic, 81% utilized additional intervention services, including the Day Centre, home support programs, and online cognitive enhancement. **Conclusions**: Profiling users of the Memory Clinic highlights the importance of expanding existing services and establishing a mobile unit to serve remote communities in Western Greece. Such initiatives could improve access to specialized dementia care, addressing geographical disparities and promoting comprehensive dementia management.

**Keywords:** memory clinic; dementia; Alzheimer’s disease; user profile; intervention

### 2.107. The Quality of Life of People with Dementia and Their Family Carers: Two Case Studies on the Impact of Home Intervention Programmes

**Zoi Asimakopoulou ^1^, Georgiana Giannakopoulou ^1^, Aggeliki Karali ^1^, Maria Frounta ^1,2^ and Ioanna Tselepi ^1^**

^1^  Frodizo-Corporation for Succor & Care of Elderly & Disabled, Patras, Greece
^2^  Medical School, University of Patras, Greece

**Abstract: Background**: Effective dementia care requires a personalized approach that addresses the needs of both individuals with dementia and their caregivers. Home-based interventions provide tailored support, enhancing quality of life and reducing caregiver burden. This presentation examines two case studies from the Home-based Dementia Intervention Program by Frodizo Association, highlighting key challenges and strategies in dementia caregiving. **Methods**: The first case explores a family caregiver providing full-time care for her mother with advanced dementia, emphasizing the psychological and emotional toll of caregiving and the importance of self-care. The second case involves a woman with vascular dementia cared for by her daughter-in-law, who has rheumatoid arthritis, illustrating the physical strain of caregiving and the challenge of managing a chronic condition while providing care. **Results**: Both cases highlight the dual focus of dementia care: managing disease progression and supporting caregivers. Implementing individualized, home-based interventions incorporating non-pharmacological and psychosocial strategies improved both the caregivers’ and care recipients’ well-being. **Conclusions:** Tailored, home-based dementia interventions are essential to addressing caregivers’ emotional, psychological, and physical challenges. Personalized strategies can enhance overall quality of life and caregiving sustainability.

**Keywords:** dementia; caregivers; home-based interventions; emotional resilience

### 2.108. Sensory Processing Disorders Affecting Memory Storage Mechanisms in Dementia

**Myrto Patagia Bakaraki ^1^, José Joaquín García Arenas ^2^ and Francisco J. Moya y Faz ^3^**

^1^  Faculty of Occupational Therapy Department, University of West Attica
^2^  Faculty of Medicine. Degree in Occupational Therapy. Catholic University of Murcia
^3^  Faculty of Medicine. Degree in Psychology. Catholic University of Murcia

**Abstract: Background:** This study investigates how sensory processing disorders affect memory consolidation and personal identity in individuals with Alzheimer’s disease (AD). Sensory impairments impact cognitive function, memory storage, and daily occupational engagement. Notably, 50% of patients reported fluctuations in self-perception due to sensory stimuli. The ADL assessment tool, with self-reported and caregiver-reported versions, showed that 65% of caregivers observed discrepancies in functional participation. These findings highlight the role of sensory processing in identity perception in AD. **Methods:** A mixed-methods approach combined standardized sensory and functional assessments with qualitative interviews. Participants included individuals with AD and their caregivers. Occupational therapy evaluations measured sensory integration challenges and their effects on memory and self-identity. Statistical analysis explored correlations between sensory deficits, memory impairments, and occupational engagement. **Results:** Sensory processing difficulties were linked to altered self-perception and reduced engagement in meaningful activities. Half of the patients experienced identity fluctuations related to sensory input, while caregiver-reported ADL scores showed functional discrepancies. These findings emphasize the role of sensory integration in cognitive health. **Conclusions:** Sensory processing deficits in AD impact memory and identity, affecting daily occupations. Occupational therapy interventions targeting sensory integration may enhance cognitive function and identity perception. Future research should explore sensory-based strategies to support meaningful engagement.

**Keywords:** dementia; ADL; sensory processing; occupational therapy; personal identity

### 2.109. Cognitive Impairment in Systemic Lupus Erythematosus and Rheumatoid Arthritis: A Cross-Sectional and Longitudinal Study

**Emmanouil Papastefanakis ^1^, Georgia Dimitraki ^1^, Georgia Ktistaki ^2^, Antonis Fanouriakis ^3,4^, Christina Adamichou ^5^, George Bertsias ^5,6^, Prodromos Sidiropoulos ^5,6^, Evangelos Karademas ^1^ and Panagiotis Simos ^2,7^**

^1^  Department of Psychology, University of Crete, Rethimno, Greece
^2^  Department of Psychiatry, School of Medicine, University of Crete, Heraklion, Greece
^3^  Department of Rheumatology, National and Kapodistrian University of Athens, Greece
^4^  Department of Rheumatology, Attikon University Hospital, Athens, Greece
^5^  Department of Rheumatology, Clinical Immunology and Allergy, University of Crete, Heraklion, Greece
^6^  Institute of Molecular Biology and Biotechnology, Foundation of Research and Technology Hellas, Heraklion, Greece
^7^  Institute of Computer Science, Foundation of Research and Technology Hellas, Heraklion, Greece

**Abstract: Background:** Systemic lupus erythematosus (SLE) often has neuropsychiatric manifestations (NPSLE), including cognitive impairment (CI). Only a few studies have examined CI in Rheumatoid Arthritis (RA). The aim of this study was to assess the prevalence of CI in three clinical groups and their differences in several CI indices. An additional aim was to examine the CI change over a year period. **Methods:** Eight two non-NPSLE, 36 NPSLE & 99 RA patients were examined with neuropsychological testing at baseline while 64, 26, 61, respectively, returned at follow-up. Chi square, McNemar tests as well as one-way and repeated measures ANOVAs were applied. **Results:** CI prevalence rates were comparable among groups (31.7–48.6%). However, NPSLE patients demonstrated poorer performance in the majority of indices compared to non-NPSLE and RA patients. Several group effects remained significant after controlling for depression, anxiety and pain. Although CI rates remained stable over time, a longitudinal improvement was observed across all three patient groups in the majority of the indices. **Conclusions:** CI mostly affects NPSLE patients, but it also manifests at significant and stable rates in the other groups. Systematic evaluation of CI in clinical practice is required, along with the development of corresponding clear intervention protocols.

**Keywords:** systemic lupus erythematosus; rheumatoid arthritis; cognitive impairment; longitudinal study

**Funding:** This study was funded in part by a grant entitled ‘Cognitive, psychosocial, and physiological aspects of patient adaptation and wellbeing in autoimmune chronic diseases: A longitudinal study of multiple sclerosis and rheumatoid arthritis”, implemented under the ‘ARISTEIA’ Action of the ‘OPERATIONAL PROGRAMME EDUCATION AND LIFELONG LEARNING’ and supported by the European Social Fund (ESF) and National Resources (Grant number 212).

### 2.110. Correlation of Amyotrophic Lateral Sclerosis with Frontotemporal Dementia and Alzheimer’s Disease—Speech and Cognitive Disorders

**Sofia G. Erkotidou ^1,2^**

^1^  University of Ioannina-Speech and Language Pathology Sector
^2^  Private Speech Therapy Centre

**Abstract:** The term dementia is a general term used to describe conditions characterized by a decline in mental functions to the point where the ability to live independently is affected. There are many causes of dementia, the most common being Alzheimer’s disease, a common feature of which is the degeneration of areas of the brain associated with cognitive functions. Frontotemporal dementia is a form of dementia caused by a family of brain disorders known as “frontotemporal lobe degeneration.” This dementia is associated with severe loss of thinking ability and interferes with a person’s ability to cope with everyday activities, such as working, driving, and preparing food. There are other brain disorders that can cause dementia, including Alzheimer’s disease and stroke. Scientists estimate that frontotemporal lobe degeneration may cause up to 10% of all cases of dementia and may be as common as Alzheimer’s disease in people up to age 65. Amyotrophic lateral sclerosis (ALS) is a fatal neurodegenerative disease that causes paralysis throughout the body. For many years, it was believed that higher nervous functions, such as cognition, were not affected in ALS. However, recent studies have shown that ALS is a multisystem disease that does not only affect motor neurons. A percentage of 10–15% of patients with ALS also experience dementia, while the majority of the remaining patients experience mild cognitive impairments involving executive functions, memory, language, and emotional processing. Amyotrophic lateral sclerosis (ALS) and frontotemporal dementia have a strong clinical, genetic, and pathological basis. The connection between them is noticeable and very important. However, the association of ALS with Alzheimer’s disease (AD) is rarely documented. Patients present with cognitive deterioration with episodic memory impairment, fulfilling the criteria for AD.

**Keywords:** amyotrophic lateral sclerosis; frontotemporal dementia; Alzheimer’s disease; cognitive disorders

### 2.111. Neoplastic Diseases–Cognitive Disorders, MCI and Speech Disorders

**Sofia G. Erkotidou ^1,2^**

^1^  University of Ioannina-Speech and Language Pathology Sector
^2^  Private Speech Therapy Centre

**Abstract:** Malignant Neoplastic Diseases are the second most common cause of death in recent years after cardiovascular diseases. In the US, approximately 1/3 of Americans develop a neoplasm during their lifetime. The most common symptom of brain tumors is a gradually worsening neurological deficit, such as weakness of a limb, difficulty understanding or pronouncing speech (dysphasia), as well as worsening headache, with a reduced level of consciousness and possible seizures. The symptoms that a brain tumor (benign or malignant) can cause depend on its location, size, and the pressure-occlusive effects it may cause. However, cognitive dysfunction is not always related to the neoplasm itself. It may be a result of chemotherapy, radiation to the brain, or co-occurring depression. The term “Mild Cognitive Impairment” (MCI) refers to a condition characterized by a decline in mental functions to a degree greater than expected for age. Sometimes, the predominant symptom of mild cognitive impairment is not related to episodic (autobiographical) memory. It may be a speech disorder or a disturbance in spatial orientation. However, amnesic-type mild cognitive impairment is the most common form. In this study, we will examine the data on cognitive and linguistic declines experienced by a patient with neoplasia, after radiotherapy, chemotherapy and immunotherapy.

**Keywords:** neoplastic diseases; cognitive disorders; speech disorders; MCI

### 2.112. Motor Neurone Disease-Dementia and Speech Therapy

**Anastasia T. Papadopoulou ^2^ and Sofia G. Erkotidou ^1,2^**

^1^  University of Ioannina-Speech and Language Pathology Sector
^2^  Private Speech Therapy Centre

**Abstract:** Motor neuron disease affects the motor nerve cells of the brain and spinal cord, leading to weakness, muscle wasting, and paralysis. The progression of this disease is relatively rapid, with the patient usually dying within 3–5 years of onset. Respiratory complications are the major cause of morbidity and mortality. The most common form of the disease is called sporadic ALS, because it seems to strike anyone at random. Familial ALS is an inherited form of the disease that affects 5–10% of cases. Scientifically, there is a traditional view that higher nervous functions, such as cognition, are not affected in ALS. However, there are reports of cognitive, behavioral, and psychiatric disorders in ALS, which have appeared since the late 19th century. Subsequent neuropsychological, neuroimaging, and neuropathological studies have shown that patients with ALS exhibit a range of cognitive impairments. It has been estimated that 3% to 10% of patients with ALS meet diagnostic criteria for dementia or aphasia. Beyond this subgroup of patients, the majority of ALS patients experience mild cognitive impairments that mainly concern executive functions, memory, language and social behavior. Speech therapy plays an important role for patients and their families. Breathing exercises and strengthening of the orofacial muscles provide security and psychological stimulation to the patient. Patients with ALS will experience difficulties in swallowing and speaking due to muscle weakness. These difficulties need to be addressed with the guidance of an experienced speech therapist, who in full collaboration with the interdisciplinary team will make the right decisions for the patient’s course.

**Keywords:** motor neuron disease–dementia and speech therapy

### 2.113. Occupational Therapy Intervention Through Reminiscence in Older Adults with Cognitive Disorders

**Ioanna Giannoula Katsouri ^1^, Nikolina Stamou ^2^, Maria-Eleni Kalamata ^3^, Georgia Tsakni ^1^, Anna Tsiakiri ^4^ and Pinelopi Vlotinou ^1^**

^1^  Department of Occupational Therapy, University of West Attica, Greece
^2^  Occupational Therapist, Day Center for Dementia Kypseli, Short-Term Dementia Care Unit and 1st Psychogeriatric Boarding House, Psychogeriatric Association “Nestor”, Greece
^3^  Occupational Therapist, Boarding House “Hippocrates II”, Social Cooperative Activities for Vulnerable Groups-K.S.D.E.O. “EDRA”, Greece
^4^  Postdoctoral Researcher, Democritus University of Thrace, Greece

**Abstract: Background:** Reminiscence-based occupational therapy intervention has been widely studied as a method of supporting older adults with cognitive impairments. Our study aims to map the existing literature, focusing on the effectiveness, methodologies, and characteristics of occupational therapy interventions incorporating reminiscence approaches. **Methods:** A comprehensive search was conducted in databases such as PubMed, PsycINFO, and CINAHL, including studies published in the last decade that examine these interventions in older adults with cognitive impairments. **Results:** The findings indicate that reminiscence therapy is frequently utilized by occupational therapists to enhance cognitive function, emotional well-being, and quality of life in this population. The effectiveness of these interventions varies, with some studies highlighting positive effects while others underscore methodological limitations. **Conclusions:** The use of reminiscence approaches in occupational therapy presents potential benefits for improving outcomes in older adults with cognitive impairments. Further research and standardized protocols are needed to comprehensively understand and measure its effectiveness. Future studies should focus on refining intervention strategies and exploring the long-term effects of reminiscence-based occupational therapy for this population.

**Keywords:** reminiscence; occupational therapy; cognitive impairments

**Funding:** University of West Attica,12241 Athens, Greece.

### 2.114. Guiding and Training: Occupational Therapy Interventions for Caregivers of Individuals with Dementia

**Pinelopi Vlotinou ^1^, Georgia Tsakni ^1^, Filomeni Armakola ^2^, Anna Tsiakiri ^3^, Konstantinos Sioumpouras ^2^, Fotini Papageorgiou ^2^ and Ioanna Giannoula Katsouri ^1^**

^1^  Assistant Professor, Department of Occupational Therapy, University of West Attica, Athens, Greece
^2^  PhD Candidate, Department of Occupational Therapy, University of West Attica, Athens, Greece
^3^  Postdoctoral Researcher, Neuropsychologist, Democritus University of Thrace, Alexandroupolis, Evros

**Abstract: Background**: Caregivers of individuals with dementia (PwD) frequently experience a heightened caregiving burden. Occupational therapy has the potential to enhance their quality of life and health through a range of therapeutic interventions. The objective of this Systematic Review was to document occupational therapy interventions aimed at caregivers of individuals with dementia. **Methods**: A comprehensive search was conducted in the databases PubMed, Scopus, EMBASE, and Web of Science, as well as in occupational therapy journals indexed in the Journal Citation Reports. The search terms included: dementia, Alzheimer’s, Parkinson’s, caregivers, and occupational therapy. Eligible articles were those with an experimental design, written in English, published in the last decade, and with full text available. **Results**: A total of 2121 articles were identified, of which 31 were included in the review. Among these, 22 articles focused on home-based occupational therapy interventions, such as the Tailored Activity Program (TAP) (n = 5), the Environment Skill-Building Program (ESP) (n = 4), and the Caregiver Training Program (ACT) (n = 3). The remaining articles described interventions conducted in other settings. **Conclusions**: Occupational therapy interventions for caregivers of individuals with dementia are predominantly delivered in home environments. The TAP was the most frequently implemented intervention, primarily aimed at reducing caregiving burden, depression, and anxiety among caregivers of individuals with Alzheimer’s disease.

**Keywords:** Alzheimer’s; dementia; occupational therapy; caregivers

### 2.115. The Economic Dimension of Home-Based Supportive Interventions in Dementia

**Georgia Tsakni ^1^, Anna Tsiakiri ^2^, Ioanna Giannoula Katsouri ^1^, Christina Ouzouni ^3^, Foteini Papageorgiou ^4^, Georgios Bablekos ^1^ and Pinelopi Vlotinou ^1^**

^1^  Assistant Professor, Department of Occupational Therapy, University of West Attica, Athens, Greece
^2^  Postdoctoral Researcher, Neuropsychologist, Democritus University of Thrace, Alexandroupolis, Evros
^3^  Professor, Department of Occupational Therapy, University of West Attica, Athens, Greece
^4^  PhD Candidate, Department of Occupational Therapy, University of West Attica, Athens, Greece

**Abstract: Background:** Dementia represents a major challenge for public health and care systems, with cases continually rising. The need for cost-effective home-based interventions to support individuals with dementia and their caregivers is urgent. However, evidence on the cost-effectiveness of these interventions remains limited. The objective of this study was to systematically evaluate the cost-effectiveness of home support interventions for dementia to inform future research and practice. **Methods:** A systematic review of economic studies was conducted, including full and partial economic evaluations of interventions, with an analysis of cost-effectiveness and quality of life outcomes. Out of the 151 articles identified, 14 met the inclusion criteria. **Results:** Interventions involving occupational therapy, home-based exercise, and psychological support were found to be the most cost-effective. Most studies indicated increased costs, but also significant benefits in quality of life. **Conclusions**: This review highlights the need for further, more comprehensive research on the cost-effectiveness of interventions at different stages of dementia, particularly in the early and late stages. Despite the diversity in outcomes, these specific interventions demonstrate a favorable cost-effectiveness ratio and can guide the development of policies for dementia care at home.

**Keywords:** dementia; cost - effectiveness; home support intervention; occupational therapy

**Funding:** The project for the conference was funded by the University of West Attica.

### 2.116. Framing Multiple Sclerosis with Non-Pharmacological Interventions: The Role of Occupational Therapy

**K. Sioumpouras** **^1^, F. Papageorgiou** **^1^, G. Tsakni** **^1^, I. Katsouri** **^1^, C. Ouzouni** **^1^, A** **. Tsiakiri** **^2^ and P. Vlotinou** **^1^**

^1^  University of West Attika
^2^  Democritus University of Thrace

**Abstract: Background:** Multiple Sclerosis (MS) is a chronic neurodegenerative disease affecting more than 2.8 million people worldwide. Non-pharmacological interventions (NPIs) have emerged as promising methods for improving the quality of life (QoL) of MS patients, although their overall impact remains unclear. This study presents a systematic review of NPIs implemented in occupational therapy practice, aiming to enhance the QoL of adults with MS. **Methods:** A comprehensive literature review was conducted in databases such as Scopus and Web of Science, focusing on randomized controlled trials evaluating NPIs in MS patients. Studies using MSQOL-54, SF-36, or MSQLI to measure QoL at multiple time points were included. **Results:** A total of 30 studies were included in the review, demonstrating a significant improvement in both physical (SMD 0.44, 95% CI 0.26–0.61) and mental (SMD 0.42, 95% CI 0.24–0.60) QoL. Physical activity interventions, particularly those involving balance exercises, showed the greatest benefits (SMD 1.71, 95% CI 1.22–2.20). **Conclusions:** NPIs, especially those incorporating physical activity, are effective in enhancing the QoL of MS patients and constitute an essential component of occupational therapy management.

**Keywords:** multiple sclerosis; MS; non-pharmacological interventions; NPIs; occupational therapy; OT; Qol

### 2.117. Parkinson’s Disease and Exercise Prescription

**Mari S. Bardopoulou**

Adapted Physical Educator, Department of Physiology, Medical School, National and Kapodistrian University of Athens, Athens, Greece

**Abstract:** Numerous studies indicate that regular physical exercise can reduce the severity of Parkinson’s disease and slow the progression of its symptoms. The aim of this presentation is to highlight specific therapeutic exercise protocols based on current guidelines for individuals with Parkinson’s. Therapeutic exercise should be a key component in the lives of those with Parkinson’s, as it plays a crucial role in maintaining balance, joint mobility, and overall improvement in daily activities. Systematic exercise and physical activity can enhance many symptoms of the disease, with research showing that people with Parkinson’s who exercise at least 3 to 4 times a week experience a slower decline in their quality of life compared to those who do not engage in physical activity. As for the type of exercise, there is no one-size-fits-all approach, as the exercise regimen depends on the individual’s symptoms and level of functional ability.

### 2.118. The Measure of Behavioral Self-Regulation “Head-Toes-Knees-Shoulders”: Preliminary Test of Its Psychometric Properties in Populations of Greek Children and Older Adults

**Dimitra Magkavila ^1,2^, Olga Fragkomichelaki ^1,2^, Magda Dinou ^1,2^, Maria Sofologi ^1,2,3^, Harilaos Zaragas ^4^, Despina Moraitou ^5,6^ and Georgia Papantoniou ^1,2,6^**

^1^  Laboratory of Psychology, Department of Early Childhood Education, School of Education, University of Ioannina, Greece
^2^  Institute of Humanities and Social Sciences, University Research Centre of Ioannina (U.R.C.I.), Greece
^3^  Department of Psychology, School of Health Sciences, Neapolis University Pafos, Paphos 8042, Cyprus
^4^  Pedagogy and Teaching Methodology Laboratory, Department of Early Childhood Education, School of Education, University of Ioannina, Greece
^5^  Laboratory of Psychology, Section of Experimental and Cognitive Psychology, School of Psychology, Aristotle University of Thessaloniki, Greece
^6^  Laboratory of Neurodegenerative Diseases, Center for Interdisciplinary Research and Innovation (CIRI - AUTH) Balkan Center, Buildings A & B, Aristotle University of Thessaloniki, Thessaloniki 57001, Greece

**Abstract:** This study aimed to examine the structural validity, internal consistency reliability, and convergent validity of the Head-Toes-Knees-Shoulders behavioral self-regulation measurement tool in a Greek population of children and older adults. **Methods:** The HTKS was administered to children alongside Raven’s Educational CPM/CVS and the Mini-Mental State Examination. Parents completed the Childhood Executive Functioning Inventory. For older adults, the HTKS was administered alongside the MMSE, CPM, the Number Test by Gustafsson, the Positive and Negative Affect Schedule, the Barratt Impulsiveness Scale, and the UPPS-P Impulsive Behavior Scale. The two samples consisted of 87 children (ages 4 to 8) and 88 older adults, aged over 65. **Results:** The findings confirmed the unidimensional structure of the Greek version of the HTKS and demonstrated good internal consistency reliability in both samples. In the children’s sample, good convergent validity was found with the MMSE and the CPM/CVS, while in the older adults’ sample, good convergent validity was observed with the CPM, the MMSE, and the Gustafsson’s Number Test.

**Keywords:** affect; behavioral self-regulation; HTKS; executive functions; impulsiveness; intelligence

### 2.119. Promoting Cognitive Health in Individuals with Down Syndrome Through Prevention and Management of Cardiovascular Risk Factors

**Ioannis Ventoulis**

Assistant Professor, Department of Occupational Therapy, University of Western Macedonia, Keptse Area, 50200 Ptolemaida, Greece

**Abstract:** Down syndrome (DS) constitutes a major cause of intellectual disability and is associated with an increased risk of developing Alzheimer’s disease (AD) at an early age. Cardiovascular disorders are being increasingly recognized as common comorbidities in DS, thereby hastening the age of dementia onset and resulting in rapid progression to AD. With the advancement of age, individuals with DS develop a profile of increased cardiometabolic risk, owing to age-related comorbidities, including obesity, diabetes mellitus, insulin resistance, dyslipidemia, atherosclerosis, hypertension, obstructive sleep apnea, and hyperuricemia. Therefore, management of modifiable cardiovascular risk factors is critical for the cognitive health of individuals with DS. In this regard, efforts should focus on the primary prevention of adverse cardiovascular events, such as stroke, especially in cases of increased cardioembolic risk due to underlying congenital heart disease. Likewise, screening asymptomatic adults with DS for diabetes or impaired glucose homeostasis should also be performed. In order to prevent cognitive decline in DS, a multidomain approach should be implemented according to the FINGER model, which gives emphasis on a bundle of simultaneous lifestyle measures in 5 domains, namely healthy diet, physical activity, mental stimulation, engaging in social activities, as well as monitoring and managing cardiovascular risk factors.

**Keywords:** cardiovascular risk factors; prevention; Down syndrome; Alzheimer

### 2.120. The Role of Nutrition in the Cognitive Health of Individuals with Down Syndrome

**Ioannis Ventoulis**

Assistant Professor, Department of Occupational Therapy, University of Western Macedonia, Keptse Area, 50200 Ptolemaida, Greece

**Abstract:** Individuals with Down syndrome (DS) run an increased risk of developing cardiometabolic disorders, mainly owing to the fact that they generally lead a lifestyle characterized by limited physical activity and unhealthy dietary habits. Considering that the unfavourable cardiometabolic profile of individuals with DS exerts untoward effects on their mental health, it is of utmost importance to implement healthy lifestyle measures in this vulnerable population that would promote their cognitive health and quality of life. Such lifestyle measures include adopting a healthy diet, monitoring weight and caloric intake closely, and performing regular exercise. In overweight and obese individuals with DS, behaviour-based weight loss interventions should be pursued in order to prevent obesity-related morbidity and mortality. To this end, adherence to a Mediterranean diet has been associated with a reduced risk of cognitive decline and progression to Alzheimer’s disease. A fiber-rich diet based on abundant consumption of fruits, vegetables, whole grains and legumes, combined with frequent intake of fish rich in omega-3 content and restricted consumption of saturated fatty acids, has been shown to promote the health of both the nervous and cardiovascular systems. Basically, a diet that exerts favourable effects on the heart is also beneficial for the brain.

**Keywords:** nutrition; cognitive health; Down syndrome; Alzheimer

### 2.121. Cognitive Training and Social Integration as Methods for Preventing Cognitive Decline in Individuals with Down Syndrome

**Raphaella Paradisi ^1,2,4^, Eleni Baldimtsi ^,2,4,5^, Panagiotis Ntailakis ^2,4^, Georgios Ntritsos ^5,6^, Georgia Papantoniou ^3,4^ and Magdalini Tsolaki ^2,4^**

^1^  Neurosciences and Neurodegenerative Diseases Postgraduate Course, Medical School, Faculty of Health Sciences, Aristotle University of Thessaloniki, 54124 Thessaloniki, Greece;
^2^  Department of Caregiver’s Support, Greek Association of Alzheimer’s Disease and Related Disorders (GAADRD), 54643 Thessaloniki, Greece;
^3^  Laboratory of Psychology, Department of Early Childhood Education, School of Education, University of Ioannina, 45110 Ioannina;
^4^  Laboratory of Neurodegenerative Diseases, Center for Interdisciplinary Research and Innovation (CIRI-AUTH), Balkan Center, Aristotle University of Thessaloniki, 10th km Thessaloniki-Thermi, 54124 Thessaloniki, Greece;
^5^  1st Department of Neurology, Medical School, Faculty of Health Sciences, Aristotle University of Thessaloniki, Greece;
^6^  Department of Economics, School of Economics and Management Sciences, University of Ioannina, Ioannina, Greece
^7^  Department of Informatics and Telecommunications, School of Informatics & Telecommunications, University of Ioannina, Arta, Greece;

**Abstract:** Down syndrome is considered the genetic version of early-onset Alzheimer’s disease. A significant percentage of adults with Down syndrome will develop the disease due to the trisomy in chromosome 21, which carries an extra copy of the gene responsible for the production of the amyloid precursor protein. Research studies indicate that in the early stages of the disease, there is a decline in abilities such as visuospatial working memory, hand-eye coordination, and linguistic semantic fluency. Behavioral and personality changes, including reduced interest in social interactions and daily activities, increased irritability, and difficulties with cooperation, are also early signs of the disease. For this reason, psychosocial interventions that aim to strengthen both the cognitive and social profiles of individuals with Down syndrome are crucial, as these factors have been shown to contribute in the prevention of the disease in the general population. This presentation will highlight the importance of cognitive training and social integration in preventing Alzheimer’s disease in adults with Down syndrome. It will also discuss the efforts of researchers from Horizon 21 to develop and implement psychosocial interventions tailored to meet the needs of this specific population, in line with the Finnish Geriatric Intervention Study model (Finger-DS).

**Keywords:** down syndrome; Alzheimer’s disease; prevention; FINGER-DS

### 2.122. Life Expectancy and the Ultimate Cause of Death in Late-Stage Dementia Patients of the Home Care Group of the Hellenic Association of Alzheimer’s Disease and Related Disorders in Thessaloniki

**Magdalini Ouzouni, Psychologist**

Aristotle University of Thessaloniki

**Abstract:** The life expectancy of late-stage dementia patients depends on various factors, such as the patient’s age, general health, and the presence of a supportive environment. In the final stage of dementia, patients typically lose the ability for self-care and independence and face severe complications, such as infections or cardiovascular problems. Life expectancy in the final stage ranges between 1 and 3 years (M = 2.1), with some cases reaching or exceeding 5 years (Max = 27), depending on the patient’s condition, also confirmed by the present descriptive study of 1042 deceased dementia patients. The increased vulnerability to infections and the inability of the body to meet basic needs make individuals with late-stage dementia susceptible to severe complications, which finally lead to death. The ultimate cause of death is usually related to complications such as pneumonia, respiratory failure, or cardiovascular diseases. In any case, family support and proper medical supervision are crucial for improving the quality of life of patients and managing the complications of late-stage dementia at home.

**Keywords:** late-stage dementia; cause of death; life expectancy

### 2.123. Modified Recall Test for the Diagnosis of Alzheimer’s Disease Dementia in the Greek Sample of Adults with Down Syndrome: A Validation Study

**Raphaella Paradisi ^1,2,4^, Eleni Baldimtsi ^2,4,5^, Panagiotis Ntailakis ^2,4^, Georgios Ntritsos ^5,6^, Georgia Papantoniou ^3,4^ and Magdalini Tsolaki ^2,4^**

^1^  Neurosciences and Neurodegenerative Diseases Postgraduate Course, Medical School, Faculty of Health Sciences, Aristotle University of Thessaloniki, 54124 Thessaloniki, Greece
^2^  Department of Caregiver’s Support, Greek Association of Alzheimer’s Disease and Related Disorders (GAADRD), 54643 Thessaloniki, Greece;
^3^  Laboratory of Psychology, Department of Early Childhood Education, School of Education, University of Ioannina, 45110 Ioannina, Greece;
^4^  Laboratory of Neurodegenerative Diseases, Center for Interdisciplinary Research and Innovation (CIRI-AUTH), Balkan Center, Aristotle University of Thessaloniki, 10th km Thessaloniki-Thermi, 54124 Thessaloniki, Greece;
^5^  1st Department of Neurology, Medical School, Faculty of Health Sciences, Aristotle University of Thessaloniki, Greece
^6^  Department of Economics, School of Economics and Management Sciences, University of Ioannina, Ioannina, Greece
^7^  Department of Informatics and Telecommunications, School of Informatics & Telecommunications, University of Ioannina, Arta, Greece

**Abstract: Introduction:** Alzheimer’s disease (AD) is significantly prevalent among adults with Down syndrome (DS). Diagnosing the disease in this population is challenging due to pre-existing intellectual disabilities. The aim of this study is to assess the ability of the modified recall test (mCRT) to identify individuals exhibiting symptoms of AD. **Method:** We conducted a cross-sectional multicenter study with 65 participants aged 18 to 65 years. Detailed medical history, demographic data, and structured interviews with caregivers were obtained to create a comprehensive cognitive profile for each participant. **Results:** Statistically significant negative correlations were found between age and performance on all mCRT main scoring scales. Significant differences were also identified between the groups of mild and moderate intellectual disability on all key scoring scales of the mCRT, and between the groups of cognitively healthy and cognitively impaired participants regarding their performance on immediate and delayed recall (*p* < 0.001). The ROC analysis showed that the tool demonstrates high sensitivity in detecting AD, indicating that participants with a score below 26.5 (on a 0–36 scale) are highly likely to exhibit AD symptoms.

**Keywords:** diagnostic tool; Alzheimer disease; down syndrome; modified cued recall test

### 2.124. Daily Functioning and Cognitive Decline in Down Syndrome, Using the Dementia Questionnaire for People with Learning Disabilities (DLD)

**Panagiotis Ntailakis ^1,5^, Raphaella Paradisi ^1,2,5^, Eleni Baldimitsi ^1,3,5^, Georgia Papantoniou ^4,5^ and Magdalini Tsolaki ^1,5^**

^1^  Greek Association of Alzheimer’s Disease and Related Disorders (GAADRD), Thessaloniki, Greece
^2^  Neurosciences and Neurodegenerative Diseases, Postgraduate Course, Medical School, Faculty of Health Sciences, Aristotle University of Thessaloniki, Greece
^3^  1st Department of Neurology, Medical School, Faculty of Health Sciences, Aristotle University of Thessaloniki, Greece
^4^  Laboratory of Psychology, Department of Early Childhood Education, School of Education, University of Ioannina, Greece
^5^  Laboratory of Neurodegenerative Diseases, Center for Interdisciplinary Research and Innovation (CIRI-AUTH), Balkan Center, Aristotle University of Thessaloniki, 10th km Thessaloniki-Thermi, Greece

**Abstract: Background:** According to recent scientific studies and the project Horizon 21, individuals with Down Syndrome have an increased risk of cognitive decline. Therefore, an early diagnosis is mandatory in order to ensure the well-being of the patients with the use of effective screening tools that assist the healthcare professional. **Methods:** The Dementia Questionnaire for People with Learning Disabilities (DLD) is a validated tool that assesses cognitive and behavioral domains of patients. The DLD is completed manually by caregivers who are well-acquainted with the individual’s daily functioning and behaviour. Early detection of cognitive decline is based on observations of the caregiver over the last two months and the responses are coded using a three-point scale, with the final assessment performed by a trained healthcare professional. **Results:** The application of the DLD has proven to be an effective tool for the early identification of dementia in individuals with Down Syndrome. By providing quantifiable data, the questionnaire aids in the systematic evaluation of cognitive decline, supporting the diagnostic procedure. **Conclusions:** Early detection of cognitive decline and dementia in individuals with Down Syndrome is crucial for improving patient outcomes and enhancing their quality of life. The DLD serves as a reliable and practical assessment tool which assists the diagnosis.

**Keywords:** down syndrome; cognitive decline; dementia; learning disabilities; DLD; Horizon 21

### 2.125. Investigation of Musculoskeletal Disorders in Caregivers of People with Dementia

**Marianna Paparnaki ^1,2^**

^1^  Aristotle University of Thessaloniki
^2^  Physiotherapist, Greek Association of Alzheimer Disease

**Abstract: Background:** Caregivers of people with dementia often experience musculoskeletal pain and discomfort. However there is limited data regarding the association of such problems to the caregiving demands. The aim of this study is to investigate musculoskeletal disorders in carers of people with dementia. **Methods:** The study participants come from home-care programmes and completed a questionnaire providing information on (a) sociodemographic and caregiving characteristics (b) musculoskeletal symptoms (via the Nordic Musculoskeletal Questionnaire) and (c) the main causes of these disorders. **Results:** The results showed that only 8% of caregivers did not experience any musculoskeletal complaints in the past year. The main cause appeared to be physical strain from caregiving (58.4%), which was associated with back discomfort (*p* < 0.001), followed by emotional stress and anxiety (34, 7%) which was associated with neck (*p* < 0.001), shoulder (*p* = 0.011) and back (*p* = 0.030) discomfort and age (33%) which appeared to be associated with neck (*p* = 0.043), back (*p* < 0.001) and arm (*p* = 0.007) discomfort. **Conclusions:** This study showed that caregivers are at risk of musculoskeletal disorders due to the demands of care, therefore training and guidance from qualified health professionals is necessary to prevent or reduce the occurrence of musculoskeletal disorders.

**Keywords:** musculoskeletal disorders; caregivers; home care; dementia

### 2.126. The Effect of THERA–Trainer Rehabilitation Equipment on Patients with Multiple Sclerosis

**Vasiliki Garopoulou ^1^, Christos Mouzakidis ^2^, Evangelos Portelanos ^3^, Odysseas Kapousizis ^4^ and Magdalini Tsolaki ^5^**

^1^  Medical school, Aristotle University of Thessaloniki, A.U.Th. Greece Panhellenic Institute of Neurodegenerative Diseases Thessaloniki, Greece Hellenic Association of Alzheimer’s Disease and Related Disorders (Alzheimer Hellas)
^2^  Hellenic Association of Alzheimer’s Disease and Related Disorders (Alzheimer Hellas)
^3^  “Neuroscience and Neurodegeneration” M.Sc. Medical School A.U.Th. Greece
^4^  Panhellenic Institute of Neurodegenerative Diseases Thessaloniki School of Chemistry, A.U.Th.
^5^  Medical school, Aristotle University of Thessaloniki, Thessaloniki, Greece Department of Classics Neuropsychiatrist, Lab of Neurodegenerative Diseases, CIRI, A.U.Th.

**Abstract:** Multiple Sclerosis (MS) is a chronic autoimmune inflammatory demyelinating disease of unknown etiology that causes generalized degeneration of the CNS, leading to severe neurological deficits. **Background**: Most affected individuals are young 20–40 years of age with an incidence in women being three times higher than in men (3.5/1). Because of its unpredictable course and the continuing trend of progressive disability, a patient’s needs change over time with long-term effects on their quality of life. The aim of this study was to investigate the effects of an intervention combining aerobic and anaerobic exercise with resistance training and use of THERA-Trainer rehabilitation equipment in people with MS. **Methods:** 14 participants with MS (n = 14), mean age 59.1 years, were included in this experimental study. A therapeutic exercise protocol of 60 min, 2f/week for 20 weeks was applied. Participants were assessed before and after the intervention using the following tools: BMI, BSA, MMSE, Senior Fitness Test, ABC, T25-FW, BBS, FSS, EDSS, EQ-5D, SF-12, HADS-7, MSQOL-54. **Results:** Our study showed that exercise is an important strategy to mitigate and/or improve functional status decline, quality of life and promote cognitive improvement in patients with PD. **Conclusions:** The results motivate further scientific investigation.

**Keywords:** multiple sclerosis; Exercise; Rehabilitation; Quality of life

### 2.127. Home-Based Occupational Therapy Intervention for the Improvement of Quality of Life in Individuals with Moderate to Severe Dementia and Their Caregivers

**Theodosis Georgiadis ^1^, Ioanna Giannoula Katsouri ^2^, Charalampos Tsormpatzoudis ^3^ and Magdalini Tsolaki ^4^**

^1^  MSc, Occupational Therapist, Thessaloniki, Greece
^2^  School of Health and Care Sciences, Chair of the Department of Occupational Therapy, University of West Attica (UNIWA); Postdoctoral Researcher, Department of Medicine, Aristotle University of Thessaloniki (AUTh)
^3^  Professor of Sports Psychology, Director of the Laboratory of Humanities Research, Department of Physical Education and Sport Science, Aristotle University of Thessaloniki (TEFAA AUTh)
^4^  Emeritus Professor of Neurology, Aristotle University of Thessaloniki (AUTh)

**Abstract:** The Quality of Life (QoL) of individuals with dementia is influenced by their ability to perform Activities in Daily Living (ADLs), engagement in social activities, and balanced emotional experiences. The aim of the present study is to investigate whether home-based Occupational Therapy intervention affects QoL parameters of people with dementia, reduces the burden, depression, and anxiety experienced by caregivers, and improves the execution of Basic ADLs. The study included 26 participants in the intervention group (Mean Age = 83.5, TA = 2.63) and 23 participants in the control group (Mean Age = 80.0, TA = 2.63). All participants were diagnosed with moderate to severe dementia. The results of the study showed a positive effect of home-based Occupational Therapy intervention on QoL parameters (interpersonal relationships, environmental factors, functionality, physical and psychological well-being) (*p* = 0.008), as well as on emotional factors (*p* = 0.009). Additionally, the results showed a positive effect on caregiver anxiety (*p* < 0.001) and depression (*p* < 0.001). However, the intervention did not improve the use of basic ADLs (*p* = 0.857) or reduce the caregiver burden (*p* = 0.773). These results allowed that both patients and caregivers benefit from home-based standardized Occupational Therapy interventions.

**Keywords:** moderate and severe dementia; activities of daily living; home-based standardized occupational therapy; quality of life

### 2.128. Quality of Life and Cognitive Impairment in Schizophrenia: The Role of Occupational Therapy Interventions

**Elpida Stratou ^1^, Georgios Pierrakos ^2^, Christina Athanasopoulou ^1^ and Ioanna Giannoula Katsouri ^1^**

^1^  Department of Occupational Therapy, School of Health and Care Sciences, University of West Attica
^2^  Department of Business Administration, School of Administrative, Economics and Social Sciences, University of West Attica

**Abstract: Background:** Schizophrenia is a severe psychiatric disorder characterized by significant cognitive impairment, affecting attention, memory, and executive functions. These deficits have a profound impact on quality of life, social participation, and daily functioning. This review examines the role of occupational therapy in enhancing cognitive and functional abilities and improving quality of life in individuals with schizophrenia. **Methods:** A systematic literature review was conducted using PubMed, CINAHL, and Cochrane Library databases. The search included studies published between 2014 and 2024, using keywords such as ‘schizophrenia’, ‘cognitive impairment’, ‘quality of life’, and ‘occupational therapy’. Studies were selected based on their focus on cognitive deficits in schizophrenia, its impact on quality of life, and the effectiveness of occupational therapy interventions. **Results:** Occupational therapy interventions, including cognitive rehabilitation, task-oriented training, metacognitive strategies, and functional skill development, have demonstrated significant improvements in cognitive performance, adaptive daily functioning, social reintegration, and overall quality of life in schizophrenia. **Conclusions**: Occupational therapy plays a key role in improving quality of life in individuals with schizophrenia. Future research should explore the long-term effects of interventions and the integration of occupational therapy within multidisciplinary care models to optimize patient outcomes.

**Keywords:** schizophrenia; cognitive impairment; quality of life; occupational therapy

**Funding:** Τhe project for the conference was funded by the University of West Attica.

### 2.129. Mobile Dementia Units—The Burden of Caregivers

**Eleni-Kyriaki Tsoula**

Alzheimer Athens, Mobile Unit for People with Dementia in Arta

**Abstract: Background:** Dementia imposes a significant burden on caregivers, affecting them physically, psychologically, emotionally, socially, and financially. The emotional strain experienced by caregivers is influenced by multiple factors, including the nature and severity of the patient’s symptoms, their pre-morbid personality traits, and the availability of resources and support systems both prior to and throughout the progression of the disease. Mobile Dementia Units, staffed by specialized healthcare professionals, play a critical role in mitigating the caregiving burden. The challenges faced by caregivers necessitate a dual-level intervention framework: (a) addressing caregiving-related responsibilities, such as financial, social, medical, and practical concerns, and (b) attending to caregivers’ emotional needs, including anxiety, depression, exhaustion, and feelings of helplessness. In response to these challenges, key interventions have been developed, including psychosocial support and caregiver education, provision of information on social and welfare policies, guidance on legal rights, and referral to appropriate healthcare and social care services. The delivery of both emotional and practical empowerment, coupled with structured guidance for caregivers of individuals with dementia, constitutes a fundamental objective of the specialized services offered by Mobile Dementia Units. **Conclusions:** Through a multidisciplinary approach, these units contribute to enhancing the well-being of caregivers while promoting sustainable and effective dementia care strategies.

**Keywords:** dementia; burden; caregivers; Mobile Units

### 2.130. Psychological Support Groups for Caregivers of People with Dementia: Conclusions and Reflections

**Nikolaos Bilanakis**

Alzheimer Society of Athens, Day Center for People with Dementia in Arta

**Abstract: Background:** Caregivers of people with dementia are a group of people who are particularly burdened. Although may differ from each other depending on their characteristics, those caregivers who receive services from a rural Day Center for people with dementia (such as ours, in Arta) can be considered to be additionally characterized by a lack of genuine interest in receiving psychological support, ambivalent feelings about their revealing participation in a psychotherapeutic group in a such a small community, insufficient knowledge about dementia, etc. **Methods:** In order to create effective psychological support groups for caregivers, we decided to create “closed” groups, for children and for spouses of patients separately, which will meet one hour per week for ten weeks, with a specific topic per session (e.g., my relation with my parents/spouse, dementia symptoms, stigma and dementia, caregiver burden), with a 15-min presentation on each topic by the group leader. The presentation will be followed by a discussion among the group members focused on the specific topic of the meeting. During the session, the Yalom’s therapeutic factors of Group Therapy will be developed (instillation of hope, interpersonal learning, universality, etc.) and the participants will take the opportunity to increase self-understanding, to change their misbehaviors and strengthen their mental health. **Results-Conclusions:** This specific Group model seems to have satisfactory results.

**Keywords:** Psychological Support Groups; Carers of people with dementia; Yalom’s therapeutic factors

### 2.131. Investigating the Effectiveness of Physiotherapy in Patients with Advanced Dementia

**Konstantina Giasirani ^1^, Vasiliki Garopoulou ^2^, Charalambos Tsorbatzoudis ^3^ and Magdalini Tsolaki ^4^**

^1^  Physiotherapist, Dementia End-Stage Care Facility
^2^  Medical school, Aristotle University of Thessaloniki, A.U.Th. Greece Panhellenic Institute of Neurodegenerative Diseases Thessaloniki, Greece Hellenic Association of Alzheimer’s Disease and Related Disorders (Alzheimer Hellas)
^3^  Professor, Faculty of Physical Education and Sport Sciences, Aristotle University of Thessaloniki
^4^  Medical school, Aristotle University of Thessaloniki, Thessaloniki, Greece Department of Classics Neuropsychiatrist, Lab of Neurodegenerative Diseases, CIRI, A.U.Th.

**Abstract:** Dementia is a clinical syndrome characterized by memory impairment and cognitive dysfunction. The increase in the elderly population with cognitive decline is a global challenge and concern. Despite scientific efforts, the eradication and immediate treatment of dementia remains unattainable. **Background**: In recent years, there has been growing interest in exercise as a therapeutic strategy for addressing cognitive dysfunction. Physical inactivity is one of the seven main modifiable risk factors for Alzheimer’s disease. **Methods:** The aim of this study is to assess the motor status of 23 patients with dementia in a palliative care unit using the MARIA scale to determine any progress or maintenance of their motor skills following individual physiotherapy interventions. Measurements were taken three times per patient, considering their functional level. The data were statistically analyzed using *t*-tests and ANOVA to determine if there was significant improvement or maintenance of functionality during the interventions. Additionally, factors such as age and education level were examined, as they may influence the effectiveness of the treatment. **Results:** The findings of this study demonstrate a significant positive impact of physiotherapy on patients’ motor function, with a statistically significant improvement in functionality, particularly between the first and third assessments. The observed mean difference of 565 points between these two time points provides strong evidence supporting the efficacy of the intervention. Furthermore, the progressive nature of the improvement is reflected in the absence of statistically significant differences between the first and second assessments. These results highlight the critical role of physiotherapy as a therapeutic approach for supporting patients with dementia, particularly within a healthcare framework where standard care primarily focuses on disease management rather than actively enhancing functional capacity. **Conclusions:** Although it is often suggested that social or demographic factors, such as gender or educational background, may influence treatment outcomes, the findings of this study indicate that physiotherapy exerted a comparable effect regardless of these variables in patients with end-stage dementia. This suggests that physiotherapy is a highly adaptable intervention capable of delivering therapeutic benefits across diverse patient populations, irrespective of individual characteristics.

**Keywords:** physiotherapy; end-stage dementia; motor status; quality of life

### 2.132. Programme “Symparastasi”: Psychoeducation and Physical Exercise in Caregivers of Patients with Mild Dementia

**Tatiana Dimitriou ^1^ and Evangelia Zacharia ^2^**

^1^  Medical School of Aristotle University of Thessaloniki
^2^  Department of Physical education and sport science

**Abstract:** Online programme “Symparastasi” was created during the COVID-19 quarantine, in order to help people who needed professional help. Via website that was created and via video-lessons from experts it aims to educate the caregivers of patients with mild dementia in non-pharmacological interventions and in physical exercise. Several psychometric tests were used in order to evaluate cognitive decline and physical skills. 426 patients were randomly assigned into 3 groups of 142 participants each and the results were the following: group A received only the multicomponent exercise, group B received only the psychoeducation and group C received both interventions. Group C had the most significant results in 3 domains: (a) they sustained the cognitive skills of their patients, (b) the behavioural and psychological problems in dementia (BPSD) were decreased and (c) the general quality of both patients and caregivers was better. However, the good results did not remain after 3 months by the end of the interventions. On the other hand, a combination of non-pharmacological interventions was found, that the caregivers could apply to their patients, with safety, in order to have better mental and cognitive health results.

**Keywords:** apathy; mild cognitive impairment; apathy evaluation scale; Alzheimer’s disease

### 2.133. The Positive Impact of Music on Alzheimer’s Disease: Research Evidence, Neurological Mechanisms, and Future Perspectives

**Dimosthenis Spanoudakis Adjunct Lecturer**

School of Music Studies, Aristotle University of Thessaloniki

**Abstract: Background**: In recent years, research has increasingly focused on the impact of music on individuals with dementia, particularly those diagnosed with Alzheimer’s disease. Music-based interventions have been associated with improvements in cognitive function, emotional regulation, and social engagement. However, the underlying mechanisms remain only partially understood. This study aims to systematically analyze the role of music in Alzheimer’s prevention and treatment, integrating insights from cognitive musicology, music psychology, and neuroscience. **Methods:** A systematic review of observational and experimental studies published in the past three decades was conducted. The analysis included statistical data from international research focusing on music’s effects on neuroplasticity, memory retention, anxiety reduction, and mood enhancement in individuals with Alzheimer’s disease. **Results:** Findings suggest that structured musical engagement contributes to cognitive resilience, enhances emotional stability, and promotes social interaction. Additionally, evidence supports the role of music in neuroprotection and synaptic plasticity, potentially delaying disease progression. **Conclusions:** The study highlights the need for interdisciplinary research combining musicology, psychology, and neuroscience to develop evidence-based, personalized interventions. Future studies should further investigate how specific musical parameters (e.g., rhythm, melody, harmonic complexity) influence cognitive and emotional outcomes in Alzheimer’s patients.

**Keywords:** music impact; Alzheimer’s disease; neuroplasticity; non-pharmaceutical interventions

### 2.134. Serum and Cerebrospinal Fluid Reactive Oxidative Species Levels in Mild Cognitive Impairment Patients

**Stavroula Ioannidou ^1^, Argyrios Ginoudis ^2^, Kali Makedou ^3^, Magda Tsolaki ^4,5^ and Evgenia Lymperaki ^1^**

^1^  Department of Biomedical Sciences, International Hellenic University
^2^  School of Veterinary Medicine, Aristotle University of Thessaloniki
^3^  Laboratory of Biochemistry, AHEPA University Hospital, School of Medicine, Aristotle University of Thessaloniki
^4^  Greek Association of Alzheimer’s Disease and Related Disorders (GAADRD)
^5^  Laboratory of Neurodegenerative Diseases, Center for Interdisciplinary Research and Innovation (CIRI), Aristotle University of Thessaloniki

**Abstract: Background:** Reactive oxygen species (ROS) are implicated in the pathogenesis of Alzheimer’s Disease (AD). This study aimed to investigate serum and CSF ROS levels in Mild Cognitive Impairment (MCI) patients and their correlation with β-amyloid (Aβ40, Aβ42 and Aβ42/Aβ40 ratio) and phosphorylated Tau protein (P-Tau). **Methods:** This study included 90 adults, 48 females and 42 males, 55–90 years old, divided in three groups: 30 MCI (A+) patients with abnormal levels of CSF amyloid (Group A), 30 MCI (A-) patients with normal levels of CSF amyloid (Group B) and 30 (A-) healthy individuals with normal levels of CSF amyloid (Group C). Aβ40, Aβ42 and P-Tau were measured immunochemically and ROS levels fluorometrically. **Results:** There were no significant differences in ROS levels by age, gender and education level between the study groups. Serum ROS levels were significantly higher in Group A than in Group B (10.10 mM vs. 8.21 mM, *p* = 0.05) and presented positive correlation with P-Tau, and negative correlation with Aβ42 and Aβ42/Aβ40 ratio. **Conclusions:** These findings suggest that serum ROS might be further investigated as a biomarker in the early stages of AD.

**Keywords:** biomarkers; reactive oxidative species; cerebrospinal fluid; amyloid; mild cognitive impairment

### 2.135. Correlation of Brain Regions with Gait Variability in Individuals with Mild Cognitive Impairment and Dementia

**Pinelopi Vlotinou ^1^, Georgia Tsakni ^1^, Ioanna Giannoula Katsouri ^1^, Konstantinos Sioumpouras ^2^, Filomeni Armakola ^2^, Christina Ouzouni ^3^ and Anna Tsiakiri ^4^**

^1^  Assistant Professor, Department of Occupational Therapy, University of West Attica, Athens, Greece
^2^  PhD Candidate, Department of Occupational Therapy, University of West Attica, Athens, Greece
^3^  Professor, Department of Occupational Therapy, University of West Attica, Athens, Greece
^4^  Postdoctoral Researcher, Neuropsychologist, Democritus University of Thrace, Alexandroupolis, Evros

**Abstract: Background**: Gait variability is a dynamic indicator that can reflect neurological changes associated with either normal aging or mild cognitive impairment (MCI). However, the specific brain structures that are affected and linked to gait remain poorly understood. **Methods**: This review aimed to systematically assess findings from 17 neuroimaging studies sourced from the Medline database. Most studies employed a cross-sectional design, while a few focused on the simultaneous evaluation of multiple brain regions. **Results**: In older adults, temporal gait variability was associated with structural alterations in areas that regulate motor coordination and balance, such as the basal ganglia, thalamus, and primary sensorimotor cortex. Additionally, spatiotemporal characteristics of gait appeared to correlate with functional differences in the hippocampus and connective pathways. A critical role in neuroimaging assessments is played by MRI sequences SWI (Susceptibility Weighted Imaging) and T2WI (T2-Weighted Imaging), which provide detailed imaging of lesions in the substantia nigra and basal ganglia, directly associated with gait disturbances and instability. In individuals with MCI and dementia, neuroimaging correlations extend to other brain structures as well. Equally important is the use of brain volumetry with MRI, which is considered the method of choice for the early detection and monitoring of MCI. This technique allows for the quantification of degenerative changes, highlighting the progression of cognitive dysfunction and its connection to sensorimotor organization and coordination. **Conclusions:** The correlation of gait with specific brain regions emphasizes the interdisciplinary role of neuroimaging in understanding and monitoring the pathophysiological mechanisms that affect gait in individuals with MCI and dementia.

**Keywords:** dementia; gait; neuroimaging; MCI

### 2.136. Cognitive Disorders and Their Management in Primary Health Care

**Eleni Vorizanou ^1^, Nikiforos Stampabas ^2^ and Dimitrios Manaras ^3^**

^1^  Nurse, MSc, 1st Health Center of Salamina
^2^  Third-year student of the Department of Physics, University of Crete
^3^  General Practitioner, MSc, Deputy Director of the 1st Health Center of Salamina

**Abstract: Introduction:** The management of patients with mental disorders is a daily challenge for workers in primary health care. The evolution of science is rapid and pharmaceutical preparations have been developed that help patients. However, there are non-pharmacological interventions that can be applied to these patients, which cost less and have positive results, worthy of mention. Mental empowerment is the most important and most effective. **Material:** Bibliographic review Google scholar and PubMed. **Results:** Mental empowerment is a structured series of therapeutic exercises and functional activities, designed to retrain mental abilities. Articles were found that support that non-pharmacological interventions help to stop mental disorders or to stabilize the progression of the disease. Mental empowerment is based on the theory of Brain Plasticity and Mental Reserve. Synapses that are activated survive and those that are not used are limited. In some areas of the brain, new synapses can be regenerated. Neuronal plasticity is not a privilege only of healthy brains. Even in old age, new cells are produced in the hippocampus. The hippocampus is where plasticity develops and is known as NEUROGENESIS. **Conclusions:** Cognitive Enhancement interventions can halt cognitive decline and improve cognitive performance in elderly people with NID and in patients with mild dementia. In patients with moderate and severe dementia, the main goal is to delay the progression of the disease and improve the quality of life of patients and their families. The development of cognitive enhancement teams in primary care organizations by trained health professionals can become a powerful weapon in the hands of the health system. This will provide the best possible care to patients with memory problems, at the least cost.

**Keywords:** dementia; differential diagnosis; non-pharmacological intervention; cognitive enhancement

### 2.137. The Impact of Activities of Daily Living on the Quality of Life of Institutionalized People with Dementia

**Athanasia Kapralou ^1^, Thomas Chatzintounas ^2^, Maria Michalopoulou ^1^ and Evangelos Bebetsos ^1^**

^1^  Democritus University of Thrace, Department of Physical Education Sport Science & Occupational Therapy, Komotini
^2^  Democritus University of Thrace, Department of Medicine, Alexandroupoli

**Abstract:** Activities of daily living (ADLs) is a term used to describe the skills required for independent self-care. They are used as an indicator of a person’s functional status, and the inability to perform them results in non-independence. Scientific studies suggest that people with dementia who live in residential care facilities have a reduced functional status, which is associated with a significant reduction in their quality of life. The aim of this study was to conduct a systematic review of research to determine the impact of ADLs on the quality of life of people with dementia living in residential care. A systematic search of PubMed, Medline, PEDro and Scopus online databases was conducted from 2014 to 2024. The keywords were used: “activities of daily living”, “quality of life”, “nursing home”, “institutionalization” and “dementia”. The literature review identified the need for strategies to increase safety and independence, such as participation in exercise, physiotherapy and occupational therapy programmes, which can improve the ability to perform ADLs. The findings suggest that participation and successful performance of ADLs by people with dementia is an important indicator of health and has a positive impact on their quality of life.

**Keywords:** activities of daily living; quality of life; dementia

### 2.138. The Contribution of Behavioural Regulation Types to Activities of Daily Living in People with Dementia

**Athanasia Kapralou ^1^, Symeon P. Vlachopoulos ^2^, Maria Michalopoulou ^1^ and Evangelos Bebetsos ^1^**

^1^  Democritus University of Thrace, Department of Physical Education Sport Science & Occupational Therapy, Komotini
^2^  Aristotle University of Thessaloniki, Department of Physical Education and Sport Science at Serres, Laboratory of Social Research on Physical Activity, Serres

**Abstract:** The aim of the present study was to examine whether types of behavioural regulation in exercise can predict activities of daily living (ADLs) in people with dementia who exercise. **Methods**: The research sample consisted of 81 individuals with dementia, aged 64–95 years, residing in residential care facilities for the elderly in the region of Thrace. Data were collected using: (a) the Barthel Index scale and (b) the Behavioural Regulation in Exercise Questionnaire-2, validated among Greek adults with good psychometric properties and among older adults with five factors: Amotivation, External Regulation, Introjected Regulation, Identified Regulation and Intrinsic Motivation. **Results**: Results supported internal consistency across all factors (Cronbach’s *a* from 0.70 to 0.89). Linear regression analysis revealed that the types of behavioural regulations contributed significantly to the prediction of ADL in the expected direction in line with self-determination theory. **Conclusions**: In conclusion, the results of the study showed that motivation significantly predicted participation in appropriately structured programmes to improve ADL performance in people with dementia.

**Keywords:** activities of daily living; self-determination theory; exercise motivation; dementia

### 2.139. The Relationship Between Physical Exercise and Cognitive Function in Individuals over 55 Years Old

**Maria Pesli and Eleni Oka**

Aristotle University of Thessaloniki, Department of Psychology

**Abstract: Background:** As time passes, individuals experience physiological changes that affect their physical and cognitive functions to varying degrees. In older adulthood, physical activity significantly declines, while cognitive abilities also tend to deteriorate in many elderly individuals as well as some middle-aged adults. Based on these observations and relevant literature, this study examined the relationship between physical activity and cognitive function in individuals aged 55 and over. **Methods:** The study analyzed the correlation between physical activity and cognitive function through statistical methods. Data were collected from participants aged 55 and above, assessing their cognitive performance and level of physical activity. Statistical tests were conducted to determine any significant relationships. **Results:** The study’s findings did not reveal a statistically significant correlation between these two variables. Consequently, the hypothesis that physical activity contributes to the improvement of cognitive function in this age group was not confirmed. **Conclusions:** Possible explanations for this finding may be related to the influence of demographic characteristics and other factors that could mediate the relationship between physical activity and cognitive performance.

**Keywords:** cognitive function; physical activity; middle-aged adults; elderly

## References

[1] Janacsek K., Shattuck K.F., Tagarelli K.M., Lum J.A., Turkeltaub P.E., Ullman M.T. (2020). Sequence learning in the human brain: A functional neuroanatomical meta-analysis of serial reaction time studies. NeuroImage.

